# Biology and genome of a newly discovered sibling species of *Caenorhabditis elegans*

**DOI:** 10.1038/s41467-018-05712-5

**Published:** 2018-08-10

**Authors:** Natsumi Kanzaki, Isheng J. Tsai, Ryusei Tanaka, Vicky L. Hunt, Dang Liu, Kenji Tsuyama, Yasunobu Maeda, Satoshi Namai, Ryohei Kumagai, Alan Tracey, Nancy Holroyd, Stephen R. Doyle, Gavin C. Woodruff, Kazunori Murase, Hiromi Kitazume, Cynthia Chai, Allison Akagi, Oishika Panda, Huei-Mien Ke, Frank C. Schroeder, John Wang, Matthew Berriman, Paul W. Sternberg, Asako Sugimoto, Taisei Kikuchi

**Affiliations:** 1 Forestry and Forest Products Research Institute, Tsukuba, 305-8687 Japan; 2 Biodiversity Research Center, Academia Sinica, Taipei city, 11529 Taiwan; 3 Faculty of Medicine, University of Miyazaki, Miyazaki, 889-1692 Japan; 4 Laboratory of Developmental Dynamics, Graduate School of Life Sciences, Tohoku University, Sendai, 980-8577 Japan; 5 Wellcome Trust Sanger Institute, Wellcome Genome Campus, Hinxton, CB10 1SA UK; 6 HHMI and Division of Biology and Biological Engineering, Caltech, Pasadena, CA 91125 USA; 7 Boyce Thompson Institute, and Department of Chemistry and Chemical Biology, Cornell University, Ithaca, NY 14853 USA; 8 Present Address: Kansai Research Center, Forestry and Forest Products Research Institute, 68 Nagaikyutaroh, Momoyama, Fushimi, Kyoto, 612-0855 Japan; 9 Present Address: Department of Biology, Institute of Ecology and Evolution, University of Oregon, Eugene, OR 97403-5289 USA

## Abstract

A ‘sibling’ species of the model organism *Caenorhabditis elegans* has long been sought for use in comparative analyses that would enable deep evolutionary interpretations of biological phenomena. Here, we describe the first sibling species of *C*. *elegans*, *C. inopinata* n. sp., isolated from fig syconia in Okinawa, Japan. We investigate the morphology, developmental processes and behaviour of *C. inopinata*, which differ significantly from those of *C. elegans*. The 123-Mb *C. inopinata* genome was sequenced and assembled into six nuclear chromosomes, allowing delineation of *Caenorhabditis* genome evolution and revealing unique characteristics, such as highly expanded transposable elements that might have contributed to the genome evolution of *C. inopinata*. In addition, *C. inopinata* exhibits massive gene losses in chemoreceptor gene families, which could be correlated with its limited habitat area. We have developed genetic and molecular techniques for *C. inopinata*; thus *C. inopinata* provides an exciting new platform for comparative evolutionary studies.

## Abstract (web summary)

*Caenorhabditis* nematodes are important model organisms. Here, the authors report the biology and genome of *Caenorhabditis inopinata*, a first sibling species of *C. elegans*, and develop genetic and molecular techniques for *C. inopinata*.

## Introduction

C*aenorhabditis elegans*, a free-living nematode inhabiting organic matter-rich environments like rotting fruits and stems in many parts of the world, is one of the most intensively used model organisms in biological studies. Key features of this animal include it’s short and prolific life cycle, small body size, genetic manipulability, fully described developmental programme, and well-characterised genome. Commonalities of the genetic pathways in *C. elegans* with other organisms including humans led to seminal discoveries in a wide variety of research fields, including neuroscience, development, signal transduction, cell death, ageing, and small RNAs^1^. One of the few missing pieces of *C. elegans* to be an exemplary model organism has been its sibling species, which would further enhance *C. elegans’* role as a model organism by allowing evolutionary interpretations of biological phenomena through deep comparisons between species that have more recently diverged. *C. briggsae* is currently the most widely used satellite model, but at the level of DNA sequence, this species is as divergent from *C. elegans* as humans are from mice^2^. Other species, including *C. brenneri* and *C. remanei*, compose a clade with *C. briggsae* in the Elegans group but *C. elegans* remains phylogenetically distant^2^. Here, we report a discovery of the long-sought sibling species of *C. elegans*, *Caenorhabditis inopinata* n. sp. in Ishigaki Island, Okinawa, Japan (“*inopinata*” meaning surprising or unexpected in Latin as the biological and phylogenetic characters of this new species were a big surprise for the authors). Although known habitats of most other *Caenorhabditis* species are soil, rotting organic materials, and leaf-litter environments^3^, *C. inopinata* was isolated from fresh syconia of the fig *Ficus septica* and is likely to have a close phoretic association with the fig wasp *Ceratosolen bisulcatus*.

## Results and discussion

### Taxonomic description

Order Rhabditida Chitwood, 1933

Family Rhabditidae Örley, 1880

Genus *Caenorhabditis* Osche, 1952

*Caenorhabditis inopinata* n. sp.

More detailed taxonomic description of the new species is provided as the Supplementary note 1.

*Etymology*: The species was named as “*inopinata*” meaning surprising or unexpected in Latin, because of its surprising biological and phylogenetic characters.

*Materials examined*: Holotype male, four paratype males, and five paratype females were deposited in the USDA Nematode Collection, Beltsville, MD, USA, and five paratype males and five paratype females are deposited in the Forest Pathology Laboratory Collection of Forestry and Forest Products Research Institute (FFPRI), Tsukuba, Japan.

The wild type strain NK74SC is available on request from Miyazaki University (T. Kikuchi) or FFPRI (N. Kanzaki).

*Locality*: The wild type strain was collected from the figs of a single tree of *Ficus septica* Burm.f. obtained from Ishigaki Island, Okinawa, Japan (GPS: 24°24′38.06″N, 124°11′06.81″E, 71 m a.s.l.) on 6 June, 2013.

**Fig. 1:**
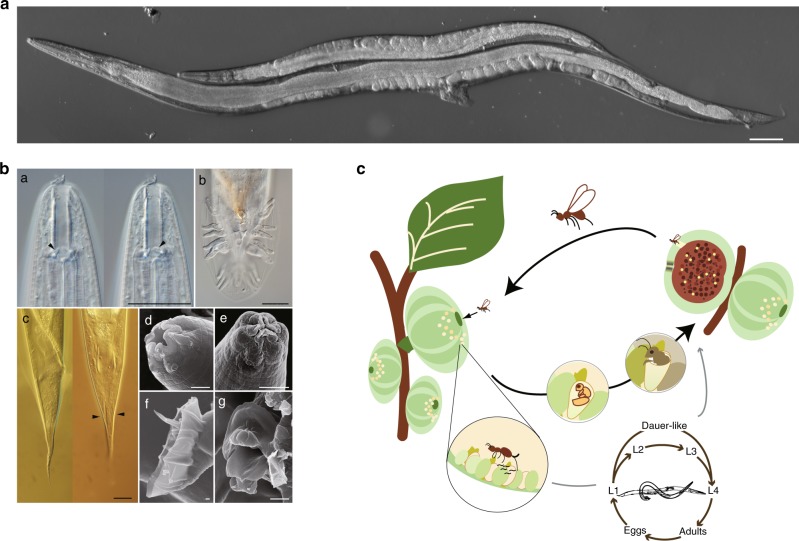
*C. inopinata* biology compared with the sibling species *C. elegans*. **a** DIC microscopic views of *C. inopinata* (adult female; bottom) with *C. elegans* (hermaphrodite; top). Scale bar: 100 μm. **b** Key morphological characters of *C. inopinata*. a: right lateral view of stomatal part in different focal planes showing dorsal tooth (left) and right subventral tooth (right) with arrow heads. b: ventral view of male tail. c: female tail in right lateral (left) and ventral (right) view showing phasmids in arrowheads. d: face view of stomatal part of adult. e: face view of stomatal part of dauer-like juvenile. f: left lateral view of male tail. g**:** ventral view of male cloacal opening. Scale bars: 20 μm for a–c, 2 μm for d–g. **c** Life cycle of *C. inopinata*. The nematodes multiply in fresh syconia of *Ficus septica*, which has a mutualistic association with the pollinating wasp *Ceratosolen* sp. When the syconia mature nematodes in the dauer form disperse to new young syconia using the wasps as a vector

*Diagnosis*: New species is characterised by the presence of short stomatal flaps, slender body, short female tail, and rounded appendage on anterior cloacal lip and tongue-like postcloacal appendage of males (Fig. 1, Supplementary Fig. 1). The combination of these characters has not been reported in the other species in the genus. Molecularly, the new species is readily distinguished from its closest relative, *C. elegans* by its molecular barcode sequences, and clear phylogenetic separation (Supplementary Fig. 2a and 2b).

### Description

*Adult*: Typological characters are and the morphometric values are provided in Fig. 1, supplementary Fig. 1 and supplementary Table 1. Large and slender species; ca. 1.5–2.5 mm in length, and individuals may reach up 3.0 mm under optimal culturing conditions. Cuticle is moderate to thick with four-lined lateral field. Deirids on the lateral field, at the level slightly behind the secretory–excretory pore. Lip separated into six sectors, not clearly offset. Six labial sensilla and four cephalic sensilla present. The anterior end of each lip sector very slightly elongated and forming six stomatal flaps. Amphid small, oval pore-like, at the level of the margin of cheilo and gymnostom. Tube-like stoma separated into three parts; short tube-like cheilostom; simple tube-like gymnostom, which is weakly separated into two subsections; and tube-like stegostom covered by pharyngeal sleeve, which is separated into four subsections, prostegostom, mesostegostom, metastegostom, and telostegostom. Metastegostomatal three teeth flap-like. Pharynx separated into four sections; procorpus forming muscular tube, well-developed metacorpus (median bulb); glandular and narrow isthmus; and basal bulb with double haustrulum as the glottoid apparatus. Pharyngo-intestinal valve (cardia) prominent. Nerve ring around the middle of isthmus. Excretory pore located around the margin of isthmus and basal bulb.

*Female*: Gonadal system didelphic, amphidelphic. Anterior and posterior gonadal system are basically symmetric with each other, arranged as ovary, oviduct, spermatheca, spermathecal-uterus junction tissue, uterus and vulva/vagina from distal. Sometimes more than 20 developing eggs are deposited. Tail conical or forming slightly elongated conus with pointed tip. Anus and rectum clearly visible; three (two subventral and one dorsal) rectal glands present. Phasmid forming small pore at ca. 60% of total tail length from anus.

*Male*: Testis single, anteriorly reflexed rightwardly. *Vas deferens* occupying ca. 1/5 of total gonadal length. Tail enveloped by a closed bursa, supported by nine pairs of bursal rays. Anterior cloacal lip with a rounded and sclerotized appendage and bulge-like appendage between rounded appendage and cloacal opening; a small sensilla-like papilla on the bulge-like appendage. Posterior cloacal lip with tongue-like appendage with two cloacal sensilla. Spicules paired, separate, long and slender with evenly slightly ventrally curved blade and simply pointed tip. Gubernaculum slender, ventrally arcuate with small squared appendage at the distal end in lateral view. Bursa heart-shaped in ventral view, anteriorly closed with serrated edge; serratae obvious in anterior half and vague in posterior half; terminal notch present but unclear. The nine pairs of genital papillae or bursal rays supporting the bursal velum with an arranged (2/1 + 1 + 2 + 3).

### Morphological and ecological characteristics

*C. inopinata* has typological characters in common with the Elegans group of the *Caenorhabditis* genus, e.g., the heart-shaped male bursa which is anteriorly closed and with serrated edge^3,4^. Phylogenetic analyses of *C. inopinata* with 26 other *Caenorhabditis* species using small subunit (SSU) and large subunit (LSU) ribosomal RNA genes or 11 conserved genes (genes coding SSU and LSU rRNA, orthologues of *C. elegans ama-1*, *lin-44*, *par-6*, *pkc-3*, ZK686.3, W02B12.9, ZK795.3, Y97E10AL.2, and Y45G12B.2a)^3^, both support that *C. inopinata* is the most closely related to *C. elegans* among known species in the *Caenorhabditis* genus (Supplementary Fig. 2a, b). Regardless of its phylogenetic closeness to *C. elegans*, *C. inopinata* has substantial morphological differences from the other Elegans group species (Supplementary note 1). For example*, C. inopinata* has a long and slender body, reaching up to nearly 3 mm in length, more than twice the body length of *C. elegans* adults (Fig. 1a, Supplementary Table 1). Also, *C. inopinata* has a modification of the lip region (presence of very short flap-like extension of lip sector) and the female has a shortened, conical tail compared to *C. elegans* (Fig. 1b). These morphological differences, particularly the relatively large body, have been found in other fig-associated nematodes (Supplementary note 1) and may be adaptations to *C. inopinata*’s habitat environment.

Figs and fig wasps have a mutualistic relationship essential to their survival^5^. *C. inopinata* is likely to have a close association with the fig *F. septica* and its pollinating *Ceratosolen* wasps, because the nematode was only observed either inside the fig syconium or as dauer stage larvae around the abdomen area of the adult pollinating wasps, after emerged from the syconia (Supplementary Table 7). Also, a higher frequency of *C. inopinata* was found in figs containing wasps than figs without wasps (Supplementary Table 6). Despite our extensive sampling in Okinawa islands, no *C. inopinata* or *Caenorhabditis* worms were detected in other *Ficus* species (e.g., *F. variegata*, *F. benguetensis* and *F. superba*) sharing the habitat with *F. septica*. These observations suggest a natural history where *C. inopinata* proliferates in *F. septica* syconia and disperses from matured to new syconia using the fig wasps (Fig. 1c).

Like *C. elegans*, *C. inopinata* is well suited to rearing under laboratory conditions, and can be cultivated using the standard *C. elegans* culture method (NGM + *E. coli* OP50). When *C. inopinata* were raised on *E. coli* HT115, gonadal elongation was enhanced (Supplementary Table 3) and brood size of *C. inopinata* became larger than those on OP50. OP50 and HT115 are known to have different nutritional qualities, and affect longevity and development of *C. elegans* of certain genetic backgrounds^6–8^. Because the effect of these bacteria on *C. inopinata* were different from that of *C. elegans*, it is likely that the two nematode species have distinct nutritional responsiveness. *C. inopinata* has mouthparts characteristic of bacterivorous nematodes (Fig. 1b, Supplementary note 1) and the intestine of freshly collected wild *C. inopinata* adults contained a wide variety of bacterial species (Supplementary Table 4). Nematode population growth was similar or elevated when *C. inopinata* were raised on the bacteria isolated and cultured from the *C. inopinata* intestine, compared to growth on *E. coli* (Supplementary Fig. 3a). Together, these results suggest the main nutrient source of the nematodes is bacteria associated with the fig syconia. Metabolic pathway analysis and CAzyme analyses based on genomic data (see Genome structure and contents section below, and Supplementary note 3) revealed a similar pathway structure and genes involved in energy metabolism for *C. inopinata* and *C. elegans*, also supporting *C. inopinata* bacterivory.

The optimal culture temperature for *C. inopinata* is 25–29 °C, significantly higher than that of *C. elegans* (20 °C) (Supplementary Fig. 3b), possibly reflecting the subtropical habitats of *C. inopinata* versus Bristol, England the source of the standard N2 *C. elegans* laboratory strain.

### Genome structure and content

Like *C. elegans* and other *Caenorhabditis* species^9^, *C. inopinata* has six pairs of nuclear chromosomes (2*n* = 12) (Supplementary Fig. 4a). The *C. inopinata* genome assembly was produced by combining multiple sequencing technologies and manually finished into six chromosomes and one mitochondrial genome (Table 1) (Supplementary note 3). This 123 megabase (Mb) assembly is the second nematode species after *C. elegans*^10^ whose genome sequence information comprise solely chromosomes. By resequencing the males and females of *C. inopinata* (Supplementary Fig. 4b), we have shown that *C. inopinata* possesses an XO sexual system. Mating experiments show a slightly female-biased progeny sex ratio (Supplementary note 2). Female-biased sex ratios have also been observed in *C. briggsae* crosses^11^, while 1:1 sex ratios are observed in *C. elegans* crosses. Female-biased sex ratios are selected for under local mate competition, which *C. inopinata* is likely to be experiencing because colonisation in a fig is mostly by relatives (Supplementary note 2 and ref. ^12^). Alternatively, it could be caused by other mechanisms, including sperm competition^11^.

**Table 1:** Genome statistics

	*C. inopinata*	*C. elegans*	*C. briggsae*
Assembly size (Mb)	123.0	100.3	108.4
Number of scaffolds	6 + 1	6 + 1	367
Average (kb)	17,573	14,327	295
Largest scaff (kb)	23,638	20,924	21,541
N50 (kb)	20,595	17,494	17,485
L50 (n)	3	3	3
Gaps (bp)	413,509	0	2,967,626
GC (%)	38.47	35.44	37.35
Num. coding genes	21,608	20,247	21,814
Coding gene size (median; bp)	1992	1972	1964
Total coding genes (Mb)	66.0	63.3	64.7
Protein similarity (vs. *C. elegans*)	81.3%	NA	80.4%
Synteny coverage (vs. *C. elegans*)	76.3%	NA	68.7%
Complete BUSCOs (protein)	98.1%	99.6%	98.4%

**Fig. 2:**
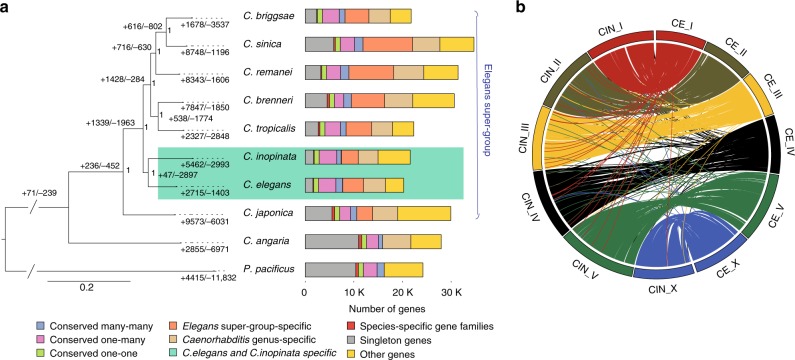
Genome of *C. inopinata* compared with *C. elegans* and other relatives. **a** Phylogeny and gene family evolution of *Caenorhabditis* species. Numbers labelled on each branch (or lineage) are the specific gain/loss of that branch (or lineage). Genes are categorised in stack bar, and the length of stack bar is proportional to number of genes. **b** Single copy orthologues linking *C. inopinata* and *C. elegans* chromosomes

A total of 21,609 gene models were predicted in *C. inopinata* aided by transcriptome sequencing of various life stages and extensive expert curation (Table 1). Of these, >95% gene models were orthologous to *C. elegans* and *C. briggsae* genes involved in well-studied biological pathways, including insulin/insulin-like growth factor signalling, sex determination, dauer formation, small RNA pathways, and nuclear receptors (Supplementary Table 15–19), indicating essential genes among the three species (*C. elegans*, *C. briggsae* and *C. inopinata*) are well conserved, and supporting a high quality of our gene predictions in the *C. inopinata* genome. Orthologue clustering was inferred between *C. inopinata* and nine other *Caenorhabditis* species, confirming its closest relationship to *C. elegans* (Fig. 2a). Divergence time estimation placed the separation of the two species 142.73 million generations ago. Assuming average generation time of 30 days, *C. inopinata* diverged from *C. elegans* about 10.5 mya (Supplementary Fig. 4c, Supplementary note 3).

Although the vast majority of *C. elegans* genes were conserved, a small number of important *C. elegans* genes were missing in *C. inopinata*. For example, among the sex determination genes, the orthologue of *C. elegans her-1* was apparently disrupted by LTR insertion (Supplementary Table 17). Additionally, although *C. inopinata* has FTR (*fog 2*-related) gene family to which *fog-2* belongs, the orthologue of *fog-2*, an essential gene for hermaphroditism in *C. elegans*, was absent in *C. inopinata* (Supplementary Table 17), as observed in *C. briggsae*^13^.

### Experimental tractability

**Fig. 3:**
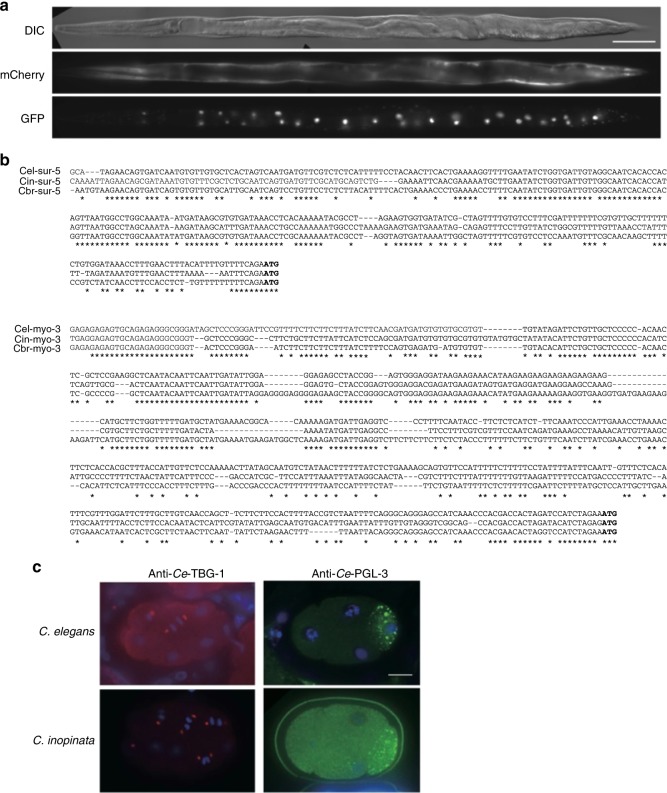
Conservation of regulatory sequences and amino acid sequences between *C. elegans* and *C. inopinata*. **a** Transgenesis in *C. inopinata* using *C. elegans*-derived transgene. Ce-MYO-3::mCherry (body wall muscle: bottom) and Ce-SUR-5::GFP (somatic nuclei: bottom) signals in an adult female animal are shown. Scale bar: 100 μm. **b** Comparison of promoter regions of the *sur-5* and *myo-3* gene. **c** Immunofluorescence using antibodies against *C. elegans* proteins. Anti-Ce-TBG-1 (red) stains centrosomes, and anti-Ce-PGL-3 (green) stains P granules (germ granules) in both *C. elegans* and *C. inopinata* embryos. Blue: DAPI. Scale bar: 10 μm

The high level of genetic similarity between *C. inopinata* and *C. elegans* allows many molecular techniques and resources used for *C. elegans* to be directly applied to *C. inopinata* studies. Stable transgenic lines of *C. inopinata* were successfully obtained by germline microinjection of marker plasmids commonly used in *C. elegans*, including *rol-6(d)* (dominant Roller phenotype), SUR-5::NLS::GFP (universal nuclear marker), *Podr-1*::GFP (AWC neuron marker), *Punc-122*::GFP/RFP (coelomocyte marker), and *Pmyo-3*::mCherry (body wall muscle marker). All *C. inopinata* transgenic lines showed similar phenotypes (Rol) or expression patterns to those found for *C. elegans* (Fig. 3a), suggesting that transcriptional regulation by promoters and post-transcriptional regulation by untranslated regions (UTRs) are highly conserved and compatible (Fig. 3b). In addition, a panel of antibodies against *C. elegans* proteins successfully recognised analogous structures in *C. inopinata* by immunofluorescence. For example, antibodies against *C. elegans* TBG-1 (γ-tubulin)^14^ recognised centrosomes in early *C. inopinata* embryos (Fig. 3c) as in *C. elegans* embryos, suggesting that the antibodies recognised the *C. inopinata* TBG-1 ortholog (amino acid identity to *C. elegans* TBG-1: 97.5%). Antibodies against *C. elegans* PGL-3^15^, a core component of P granules (germ granules) and one of the rapidly evolving proteins, also recognised P granule-like structures in *C. inopinata* embryos (amino acid identity between *C. elegans* PGL-3 and its *C. inopinata* ortholog: 46.8%) (Fig. 3c). Considering the high conservation of transcriptional and post-transcriptional regulatory sequences and amino acid sequences between *C. elegans* and *C. inopinata*, rich resources developed for *C. elegans*, including vector plasmids used for transgenesis and antibodies are likely to be directly usable in *C. inopinata*, which makes this organism a suitable comparative model to *C. elegans* at genetic and molecular levels.

### Chemoreceptors in *C. inopinata*

**Fig. 4:**
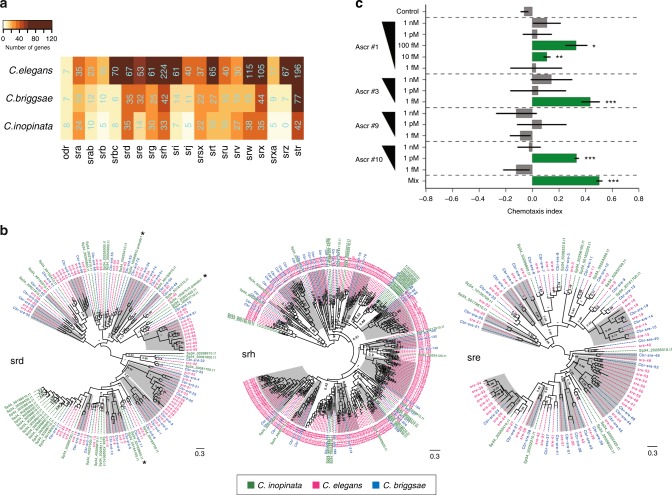
Diversity of 7TM GPCR gene families in *C. inopinata* and chemosensory response. **a** A heat map showing gene numbers of 7TM GPCR (serpentine) gene families in *C. inopinata*, *C. elegans*, and *C. briggsae*. **b** Maximum likelihood trees of *srd*, *sre*, and *srh* serpentine families of the three *Caenorhabditis* species showing gene losses, as well as local gene expansions in *C. inopinata*. Clusters with gene loss in the *C. inopinata* genome were shaded in grey. **c** Response of *C. inopinata* males to synthetic ascarosides produced by mixed stage cultures. CI chemotaxis index. Ascr#, refer to distinct synthetic ascarosides that are made by *C. inopinata*. The bars represent the mean CI and the whiskers are the SEM. The green-coloured bars indicate ascaroside concentrations that were significantly different from the control

Gene family and protein domain analyses (see Supplementary note 3) suggested that there are considerable contractions in seven-transmembrane G protein-coupled receptor (7TM-GPCR) (also called serpentine) gene families in *C. inopinata*. By intensive gene curation (Supplementary note 3), we identified a complete gene list of *C. inopinata* serpentine receptors comprising 382 genes and 48 pseudo or partial genes (Fig. 4a, Supplementary Table 20), a number significantly smaller than that of *C. elegans* (1329 genes) (*p* < 2.2e−16, Chi-squared test) and slightly smaller than *C. briggsae* (452 genes) (Fig. 4a). It has been reported that the gene number differences between *C. elegans* and other *Caenorhabditis* species were the result of gene expansion in *C. elegans* rather than gene losses in the other species^16^. Our phylogenetic analyses, however, suggested that massive gene losses clearly occurred in *C. inopinata*, such as *srd*, *sre*, *srh*, and *srt* serpentine families (Fig. 4b, Supplementary Fig. 6). These analyses also indicate that local gene expansions have occurred in specific subgroups in *C. inopinata* serpentine families, overall, retaining the total serpentine gene number to ~400 (Fig. 4b, Supplementary Fig. 6). These results suggest that *C. inopinata* requires a narrower range of receptors, compared to the ‘soil/rotting fruits’ nematodes (*C.* *elegans* and *C. briggsae*). A contraction of GPCR families could be related to its limited habitat area and close association with the fig and vector insect, in which fine detection of only a small variation of chemical stimuli may be required (Fig. 1b).

To probe the chemosensory response of *C. inopinata* to ethologically relevant molecules, we examined ascaroside signalling. *C. elegans* and other nematodes produce a wide range of ascarosides (secondary metabolites that are glycosides of the dideoxy sugar ascarylose); many behaviours are affected by particular combinations of ascarosides and many GPCRs are implicated in the ascaroside response^17^. We found that mixed stage *C. inopinata* cultures produce many of the key ascarosides found in *C. elegans* (Supplementary Fig. 3c) and *C. inopinata* males respond to the prominent ascarosides (Fig. 4c). *C. inopinata* males are more sensitive to the tested ascarosides than are *C. elegans* males, for example, responding to a lower amount of the ascr#3 (Fig. 4c). We conclude that ascaroside-mediated mate recognition is conserved between these sibling species but there are evolutionary differences of the details of that signalling, potentially reflected in their GPCR repertoire.

### Expansion of transposable elements in *C. inopinata*

**Fig. 5:**
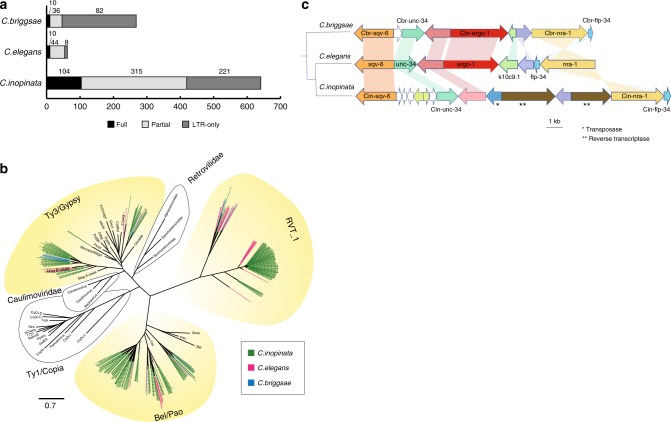
Transposon expansion and *ergo-1* gene loss in *C. inopinata*. **a** Number of LTR retro-transposable elements in *C. inopinata*, *C. elegans*, and *C. briggsae*. LTR elements were grouped into ‘Full’ with full LTR retrotransposon domain sets (reverse transcriptase; RT, protease, integrase, RNase H), ‘Partial’ with at least one of the domains but not full, and ‘LTR-only’ having only LTR regions with no protein domains. **b** Phylogenetic relationships of LTR retrotransposons in the three *Caenorhabditis* species. RT domains extracted from LTR retro-transposable elements of the three species were aligned by Mafft v7.221^52^ with reference sequences obtained from GyDB (Gypsy DataBase)^75^ and a maximum-likelihood tree was constructed using RAxML v7.2.8 using the best-fitting empirical model of amino acid substitution with 1000 bootstrap resampling replicates with the percentage support shown on the nodes. Green, red, and blue lines on the branches represent *C. inopinata*, *C. elegans*, and *C. briggsae* sequences, respectively. The scale bar shows the number of amino acid substitutions per site. **c** Gene synteny in *C. elegans*, *C. briggsae*, and *C. inopinata* for regions of the genomes corresponding to *ergo-1 C. elegans* gene. *ergo-1* is highlighted in red and orthologous genes are grouped by colour. Genes with no orthology to other genes in the region shown are white

The *C. inopinata* nuclear genome is 23 Mb larger than its sibling *C. elegans* (Table 1, Supplementary Table 9) possibly attributable to genome shrinkage associated with the evolution of selfing. Previous comparative genome studies of selfing and male/female species suggested genome degradation occurs in selfing species because of adaptive gene loss and relaxed selection on genes for the maintenance of sexual traits^18,19^. Recently, direct comparison of *C. briggsae* and her sister male/female species *C. nigoni*, confirmed extensive gene loss (up to 16%) in *C. briggsae*^20^. While gene loss may have contributed to the genome size differences, the magnitude is likely low because ~95% of *C. inopinata* genes have *C. elegans* orthologues. Another possibility is skew, a non-adaptive process of biased chromosome transmission, whereby fathers transmit the shorter allele of an autosomal chromosome pair preferentially to their daughters^21^. Skew is predicted to passively drive genome reduction in selfing nematodes and likely occurs in all *Caenorhabditis* species^22^. Consistent with this model is that intergenic distances on *C. elegans* autosomes are shorter than on their X chromosomes and shorter than on *C. inopinata* autosomes. While genome reduction has likely occurred in *C. elegans*, a third explanation is *C. inopinata* genome expansion. Indeed, the proliferation of transposons and other repetitive elements, in particular LTR retrotransposons, long interspersed nuclear elements (LINES) and Tc1/mariner transposons, in the intergenic regions are highly expanded in the *C. inopinata* genome compared to *C. elegans* and *C. briggsae* and may be the main driver for the genome size differences between these species (Supplementary Table 10, Extended Figure 7b). The *C. inopinata* genome contains 641 LTR retrotransposon elements, 104 of which contain full protein domains (intact LTRs) In comparison, *C. elegans* and *C. briggsae* have 62 and 128 LTR retrotransposon elements, respectively, with 10 intact LTRs found in each (Fig. 5a). Phylogenetic analyses of reverse transcriptase (RT) domains of LTR retrotransposon elements from *C. inopinata, C. elegans*, and *C. briggsae*, showed that three families of LTR elements have expanded in *C. inopinata* (Ty3/Gypsy, Bel/Pao LTR families and RVT_1) (Fig. 5b). All three phylogenetic clades contain between 3 and 20 sequences from both *C. elegans* and *C. briggsae* showing close relationships to *C. inopinata* RTs (Fig. 5b). This suggests that the expansion of LTR elements in *C. inopinata* is unlikely a result of new acquisition of LTR elements from an external source but mainly due to replication of elements that existed in a common ancestor.

The most common type of repetitive sequence in the *C. inopinata* genome is the TcMar transposase Tc1 family which has expanded in *C. inopinata*, comprising 8.85% of the genome, compared with 1.31% and 3.04% of the *C. elegans* and *C. briggsae* genomes, respectively (Supplementary Table 10). Phylogenetic analyses show that the *C. inopinata* TcMar transposases form five main clusters, which are related to *C. elegans* Tc1 and Tc3 transposons, *C. briggsae* Tcb1 and Tcb2 transposons, Mos1 transposons from *Drosophila* and HsMar transposons from humans (Supplementary Fig. 5). Together, these results suggest that there is a high level of transposable element activity in the *C. inopinata* genome, compared with other *Caenorhabditis* species, and this could be a driving force of the evolution of *C. inopinata*.

Orthologues of three *C. elegans* genes (*ergo-1*, *eri-6/7*, and *eri-9*) involved in the ERGO-1 26G small-interfering(si)RNA pathway are missing in *C. inopinata*, although other siRNA/microRNA related pathways are highly conserved in the three *Caenorhabditis* species (Fig. 5c, Supplementary Table 19). The absence of those genes were confirmed from several aspects of evidence including BLAST search against the gene models, genome, and transcriptome data. In *C. elegans*, the *ergo-1* pathway is likely involved in targeting and suppressing the expression of newly acquired, duplicated genes, possibly of viral origin^23^ and potentially deleterious non-coding regions of the genome^24^. Gene synteny in the *ergo-1* region of the genome is highly conserved between *C. elegans* and *C. briggsae*. In *C. inopinata*, the gene order in the analogous region of the genome has undergone some rearrangements, including the loss of *ergo-1* and hypothetical gene *K10C9.1/ cbg17878*, as well as expansion of several gene families, including mariner-like transposase (TcMar) and RT genes (Fig. 5c), which was confirmed by a long-range PCR targeting this region in *C. inopinata* using primers (ci_R09A1_f: CACATTTCGGTACTTGCCAAT and ci_Y50D4C_r: GGAATTTGATGGCATGGTT) and subsequent Sanger sequencing. Gene rearrangement in the *ergo-1* region could therefore be a consequence of DNA transposase and LTR retrotransposase activity. If the ergo-1 pathway has a role in suppressing transposases, the loss of *ergo-1*, could have further enhanced transposase activity through the reduction of 26G siRNAs in the *C. inopinata* genome.

**Fig. 6:**
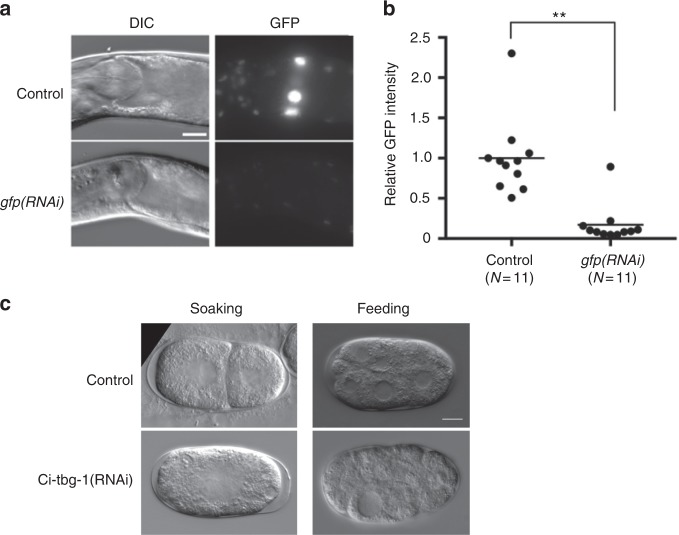
RNAi efficiency in *C. inopinata*. **a** Feeding RNAi in *C. inopinata*. The GFP encoding transgene in a transgenic line from Fig. 3a was targeted by feeding RNAi. The GFP signal was significantly reduced. Scale bar: 20 μm. **b** Efficiency of *gfp(RNAi)*. Relative intensities of the GFP signal in control and RNAi-treated worms was compared. **p < 0.01, Student's *t*-test. **c** Soaking and feeding RNAi of *Ci-tbg-1*. Embryos were arrested with cell division defects (soaking RNAi: 1-cell arrest, feeding RNAi: early embryo with some abnormally large nuclei). Scale bar: 10 μm

We found that *C. inopinata*, like the *C. elegans* N2 strain, was highly susceptible to RNA interference (RNAi) by ingestion of double-stranded RNA in both germline and soma, unlike other *Caenorhabditis* species including *C. briggsae*, *C. remanei*, and *C. brenneri*, and some *C. elegans* natural variants^25^. Feeding RNAi for *C. inopinata unc-22* orthologue resulted in 93.9% of F1 worms showing a twitching phenotype, similar to *C. elegans unc-22* mutants. Feeding RNAi against GFP transgene to a GFP *C. inopinata* transgenic line reduced GFP signal by ~80% (Fig. 6a, b), although neuronal cells appeared less sensitive. Soaking and feeding RNAi for *tbg-1* (γ-tubulin) orthologue of *C. inopinata* resulted in arrested early embryos with cell division defects (Fig. 6c), which is similar to the *C. elegans tbg-1(RNAi)* phenotype. Together, these results demonstrate efficient levels of gene silencing by RNAi methods, implying that the dsRNA uptake pathway, including SID-2, is conserved in *C. inopinata* and *C. elegans*, unlike other *Caenorhabditis* species including *C. briggsae*, *C. remanei*, and *C. brenneri*, which have diverged SID-2 functions^25^. In *C. elegans*, exogenous-RNAi and endogenous-RNAi use a common downstream pathway for target silencing. In *C. elegans*, loss of function of genes involved in the ERGO-1 class 26G RNAs pathway causes an Eri (Enhanced RNAi) phenotype, which shows enhanced sensitivity to exogenous RNAi reviewed in ref. ^26^. Therefore, the loss of key genes involved in ERGO-1 class 26G RNA pathway (*ergo-1*, *eri-6*, and *eri-9/10*) may also account for the high exogenous RNAi efficiency observed in *C. inopinata*.

### Genome structure evolution

The completeness of the *C. inopinata* reference genome allowed us to investigate the chromosome evolution in *Caenorhabditis*, especially revisiting the arm/centre definition^27,28^. Seventy-six percent of the *C. inopinata* genome can be assigned to blocks of collinear genes (synteny) based on the position of the orthologues between the three completed *Caenorhabditis* genomes (Table 1). Gene collinearity is largely conserved despite frequent intra chromosomal rearrangements (Fig. 2b, Supplementary Fig. 7a). The positions of rearrangements on autosomes appear to be independent of species relatedness, as well as chromosome arm/centre definition (Supplementary Fig. 7a). Consistent with the gene family analysis, regions without synteny (breaks) in the *C. inopinata* genome are likely due to expansion of gene families enriched in transposon-related GO terms (Supplementary Table 21). Conversely, synteny breaks in *C. elegans* are mainly due to an expansion of GPCRs gene family especially on chromosome V (Supplementary Table 21).

We observed significantly higher synonymous substitution rates (dS) in autosomal arm regions than in the centre regions for *C. elegans* and *C. inopinata* one-to-one orthologues. No difference was found between the arm and centre regions of chromosome X, and overall dS was lower in chromosome X than autosomes (Supplementary Fig. 7e). These observations suggest that intra-chromosomal translocation of *Caenorhabditis* genes may have little fitness cost, but the evolutionary processes acting upon them, as determined by dS, is dependent on the region of the chromosome and whether the chromosome is an autosome or sex chromosome. This can be demonstrated by the *Caenorhabditis* sex chromosome, which has a reduced effective population size and recombination rate^18,19,29^ (Supplementary Fig. 7d, e).

In conclusion, *C. inopinata*, the sibling species of *C. elegans*, serves as a new satellite model system that will enable deep evolutionary interpretations of biological phenomena through comparative analyses with *C. elegans* and other *Caenorhabditis* spp. Characterisation of the genome, morphology, ecology, and behaviour of *C. inopinata* has revealed robust similarities, as well as substantial differences with *C. elegans*. Notably, *C. inopinata* has highly conserved gene synteny and orthology of essential genes with *C. elegans* and *C. briggsae*, but differs substantially in morphology and ecology. We hypothesise that activation of transposable elements possibly due to de-silencing through an altered small RNA pathway could be a driving force of rapid diversification of *C. inopinata* from other *Caenorhabditis* species. The contiguous assembly of the *C. inopinata* genome and genetic data presented here, in addition to the transferability of genetic and molecular tools available for *C. elegans* to *C. inopinat*a, provides an exciting new platform to perform comparative evolutionary studies.

## Methods

### Nomenclatural acts

This published work and the nomenclatural acts it contains have been registered in ZooBank, the proposed online registration system for the International Code of Zoological Nomenclature (ICZN). The ZooBank LSIDs (Life Science Identifiers) can be resolved and the associated information viewed through any standard web browser by appending the LSID to the prefix ‘‘http://zoobank.org/’’. The LSIDs for this publication are: urn:lsid:zoobank.org:act:70EEB2AC-C773-4AC4-B73B-E59191883567.

### Discovery and observation of *C. inopinata*

About 30 of *Ficus septica* were collected manually from Ishigaki Island, Okinawa, Japan on 6 June, 2013 (GPS: 24°24′38.06″N, 124°11′06.81″E, 71 m a.s.l.). The figs were brought back to the laboratory, cut into half with a sterilised knife, and settled individually on 2.0% agar plates (⌀ = 90 mm). The plates were kept at room temperature (25 °C), and propagation of any nematode species were examined daily. The propagated nematodes were observed under light microscope to determine their food source (bacteria or fungi), and transferred to either NGM seeded with *E. coli* OP50-1 (for bacteriovorus species including *C. inopinata*) or PDA previously inoculated with *Botrytis cinerea* (for fungivorous species). The wild-type strain of *C. inopinata* was coded as NK74SC, and kept as a laboratory strain.

Male and female adults from a 2-week-old culture of the strain were hand-picked from the culture plate. Live worms were observed, drawn and micrographed under light microscope (Eclipse 80i; Nikon) equipped with DIC/Nomarski devices, drawing tube, and a digital camera system (DS-Ri1; Nikon). Permanent slides (type materials) for measurements and morphometrics were prepared from the same culture plate used for morphological observation, where hand-picked nematodes were heat-killed, fixed in TAF, processed into glycerine using modified Seinhorst’s methods and mounted in glycerine according to Maeseneer and d’Herde methods^30^.

### Establishment of an isogenic line

Gravid females from a 1-week-old culture were hand-picked and individually transferred to a small (⌀ = 60 mm) NGM plate previously seeded with *E. coli* OP50-1, and the plates were kept at 25 °C. This procedure was repeated 10 times using a successfully propagated plate in each generation. The last generation was subcultured and kept at the laboratory as the isogenic line of *C. inopinata* with a culture code NKZ35. The isogenic line served for the successive laboratory experiments.

### Genome sequencing and assembly

Illumina reads from multiple paired-end and mate-pair libraries (Supplementary Table 8), generated using DNA extracted from the *C. inopinata* isogenic line, were quality-trimmed using Trimmomatic v0.32 with options (ILLUMINACLIP:2:30:10 LEADING:10 TRAILING:10 SLIDINGWINDOW:4:15 MINLEN:36)^31^ (paired-end reads) or with the –m 50 option^32^ (mate-pair reads), and then assembled using the Platanus assembler v1.2.4^33^ with options (assembly; -k 52, scaffolding and gapclosing; default parameters). Two assemblies consisting with PacBio reads (Supplementary Table 8) were produced using Falcon v.0.3.0 with options (overlap minlen 1000 ; to graph minlen = 8000 ;—max_diff 100 —max_cov 100 —min_cov 10)^34^ and Canu v1.3^35^ with the default parameters. Consensus from both PacBio assemblies was improved using the Quiver module in the SMRT Analysis pipeline (version 2.3.0)^36^ with the default parameters. The two PacBio assemblies and the Illumina assembly were merged using Metassembler v3^37^ with the default parameters. The merged assembly was further improved using HaploMerger2 v20161205^38^ (default parameters on the repeat-masked assembly by RepeatMasker, see below) and SSPACE v3.0^39^ with options (-g 0 -x 0 -m 40 -o 15), and manually curated using gap5 v1.14.8^40^. Smaller scaffolds were defined as residual haplotypes when they were entirely covered by >90% within larger scaffolds and share half of the average coverage. These scaffolds were removed and Illumina paired end reads were remapped to check if the reads were successfully remapped to the larger scaffolds. Base and consensus correction was then performed with 8.8 Gb Illumina pair-end reads using ICORN2 v0.95^41^ with five iterations. High molecular weight genomic DNA prepared from *C. inopinata* using CHEF Genomic DNA Plug Kits (BioRad) was mapped using the IRYS Mapping System (BioNano) and was used to improve the genome assembly to order and orientate sequence scaffolds, to measure the size of sequence gaps, and independently validate the sequence assembly, producing the v7 genome assembly.

### Repeat analysis

Repeats within the assemblies were identified using the combined outputs of RepeatModeler (v1.0.4, http://www.repeatmasker.org/RepeatModeler.html) and TransposonPSI (v08222010, http://transposonpsi.sourceforge.net). UCLUST was used to cluster repeat sequences from RepeatModeler and TransposonPSI that had ≥80% identity, to generate consensus sequences for a non-redundant repeat library. RepeatMasker (v.3.2.8, http://www.repeatmasker.org) was then run (using the slow search option) with a custom repeat library for each species, to calculate the distribution of each repeat and its abundance in the genome. LTR retrotransposable elements were separately identified using a combination of LTR harvest/digest v1.5.8^42,43^ and MGEScan v1.3.1^44^. LTRharvest was used to identify potential candidates of LTRs in the genome (LTR lengths 100–1000bp, total length 1–10kbp, 85% minimum LTR similarity, TSD length 4–20bp, seed 30bp, looking for motif starting ‘tg’ and ending in ‘ca’). LTRdigest was used to subsequently annotate the candidates with protein domains from Pfam and GyDB, as well as putative primer-binding sites and polypurine tracts. MGEscan-LTR was also run on the genome to identify LTRs de novo with default parameters. The two results were processed using custom scripts (available from github.com/syubukun/Sp34) to remove low confidence candidates by only keeping (1) candidates with full LTR retrotransposon domain sets (RT, protease, integrase, RNase H), (2) candidates with at least one protein domain (*e*-value < 1e−20) but not full, and (3) candidates with only LTR regions but has high similarity (>95%) to LTRs from (1).

### Gene findings and annotation

To predict protein-coding genes, Augustus (v. 3.0.1)^45^ was trained for *C. inopinata* based on a training set of 1500 non-overlapping, non-homologous, and manually curated genes. A selection of gene models initially predicted by CEGMA v2.5^46^ and Augustus *C. elegans* parameters, were curated in Artemis^47^ using aligned RNA-seq data and BLAST matches^48^ against the NCBI database. Based on RNA-seq alignments to the genome using Hisat2 v2.0.3^49^ (parameters: —rna-strandness RF—min-intronlen 20 –max-intronlen 10,000), the bam2hints programme (part of the Augustus package) was used to create the intron hints, with minimum length set to 20 bp. The mapped RNA-seq reads were also assembled into transcripts using Cufflinks v2.2.1 with options (—min-intron-length 15, —max-intron-length 300,000). The predicted exons in the resultant set of transcripts were used as the exonpart hints. The trained versions of Augustus were run using all the hints for that species as input. If Augustus predicted multiple, alternatively spliced transcripts for a gene, we only kept the transcript corresponding to the longest predicted protein to have a total of 20,976 gene models (v7.7 gene set). Gene structures in the Augustus predictions were refined by manual curation in Artemis, using RNA-seq mapping results and BLAST result against *C. elegans* proteins. In the manual curation processes, we targeted mainly on erroneously fused gene models and GPCR genes, which we found difficult to predict using a machine prediction. If a gene was manually curated, it replaced the original Augustus gene prediction in our final gene set and this process increased the gene numbers to 21,609 (v7.10 gene set).

### Identification of gene families and gene family evolution analysis

To establish orthology relationships among *Caenorhabditis* species, non-redundant proteomes of eight *Caenorhabditis* species and an outgroup species *Pristionchus pacificus* (Supplementary Table 9) were obtained from WormBase (version WS252). OrthoFinder version 0.2.841^50^ with default options was used to assess orthology with those proteins. We used CAFE (version 3)^51^ to analyse gene family expansion and contraction under maximum likelihood framework. Gene family results from OrthoFinder and ultrametric phylogenetic tree calculated as described below were used as inputs under parameters “−p 0.01, −r 1000”.

### Species tree reconstruction

Amino acid sequences in each single-copy gene family were aligned using MAFFT version v7.221^52^, poorly aligned regions were trimmed using GBlocks v0.91b^53^, and then the 1000 trimmed alignments (all sequences in 16 alignments were removed in the Gblocks trimming) were concatenated. The best-fitting substitution model for each protein alignment was identified using the RAxML option (-m PROTGAMMAAUTO). A maximum-likelihood phylogenetic tree was produced based on the concatenated alignment using RAxML v8.2.7^54^ with 500 bootstrap replicates.

### Divergence estimate

Neutral divergence between *C. inopinata* and *C. elegans* was estimated according to the Cutter’s method^2,19^. To begin with the estimation, lineage-specific rate of synonymous (dS) and non-synonymous (dN) substitution for orthologous groups of genes between each of *Caenorhabditis* species in the phylogeny was calculated using Codeml in PAML (v4.9)^55^ with options (runmode = 0, CodonFreq = 2, model = 1, fix_omega = 0, omega = 0.01). Lineage ages (*T*) were inferred by applying median of synonymous-site divergence values and direct measures of the average per-site mutation rate in *C. elegans* (*μ* = 9.0 × 10^–9^ mutations per generation)^56^ to the equation from the neutral theory of molecular evolution *T* = dS/*μ*. Effective codon usage numbers (Nc) was calculated by DAMBE (v6)^57^ and used to adjust the divergence values upward by transforming least-squares regression of branch-specific dS to the expected dS for the *Nc*. Extreme values (dN > 0.5, dS > 5, and dS < 0.0005) were removed before estimation to avoid biases from saturation of synonymous rate between related species and miss assignment of orthologous groups.

### Functional annotation

Functional annotations were performed on the *C. inopinata* gene models (v7.10) based on multiple pieces of evidence including BLASTP search against UniProt (http://www.uniprot.org/), the latest version Pfam search (ver. 30.0)^58^ with HMMER3, InterProScan^59^, dbCAN^60^ for Carbohydrate-active enzymes (CAZymes) search, and orthology information with *C. elegans* from OrthoFinder results (for details, please see Supplementary note 3). Gene ontology (GO) terms of the three possible types (molecular function, cellular component, and biological process) were assigned to genes by transferring GO terms from the *C. elegans* orthologues. Only manually curated GO annotations were downloaded from the GO Consortium website and transferred to the *C. inopinata*. Additional GO terms were identified using Blast2Go (v2)^61^ with BLAST search against NCBI nr database and the InterProScan^59^ results.

### GPCR analyses

We manually curated 7TM-GPCR genes in the *C. inopinata* genome based on TBLATN search using *C. elegans* serpentines as queries. To see detailed relationships of *Caenorhabditis*, phylogenetic trees of genes in each serpentine family from *C. inopinata*, *C. elegans*, and *C. briggsae* were constructed using Mafft v.7.221^52^ and FastTree 2.1.8^62^.

### Synteny analysis

Using the three highly contiguous species, *C. inopinata*, *C. elegans*, and *C. briggsae* were used for synteny analysis. Synteny was detected by DAGchainer (r02062008)^63^ using the orthology of protein-coding genes assigned as described above. Synteny-linking plots were generated using segments and polygon functions in R (version 3.2.4) (for three species) and Circos (v0.67-7)^64^ (for *C. elegans* and *C. inopinata*). Bedtools (v2.17.0)^65^ was used to compute size of gene, intergenic space per synteny block and to obtain non-synteny regions along each chromosome.

### Transgenesis by microinjection

The germline transformation in *C. inopinata* was performed by microinjection according to the protocol commonly used for *C. elegans*^66^. Worms for microinjection were fed with *E. coli* OP50-1 or HT115(DE3) (which enhances germline proliferation compared with OP50-1). The plasmid mixture was microinjected into the proximal gonads of *C. inopinata* adult females and recovered worms were cultured with adult males. Transgenic progeny was screened under the dissecting microscope. Transgenic marker plasmids commonly used in *C. elegans* were used to construct transgenic *C. inopinata*. The composition of the injected plasmid mixture was the following: 40 ng/µL pRF4 (dominant *rol-6* mutant that causes dominant Rol phenotype)^66^, 35 ng/µL pTG96 (SUR-5::GFP; nuclear GFP in all cells)^67^, 40 ng/µL Podr-1::GFP (GFP in AWC neurons)^68^, 40 ng/µL pCFJ68 (Punc-122::GFP; GFP in coelomocytes, Addgene plasmid #19325, a gift from Erik Jorgensen)^69^, 40 ng/µL Punc-122::RFP (RFP in coelomocytes, Addgene plasmid #8938, a gift from Piali Sengupta)^70^, and 40 ng/µL pCFJ104 (Pmyo-3::mCherry; mCherry in body wall muscles, Addgene plasmid #19328, a gift from Erik Jorgensen)^69^. Multiple stable transgenic lines were obtained, and one of them was used for the following RNAi experiment.

### Immunofluorescence using antibodies against *C. elegans* proteins

*C. inopinata* embryos were immunostained as described for *C. elegans*^71^. Briefly, embryos were collected by cutting gravid female adults, permeabilized by freeze-cracking, and fixed with methanol-acetone. Polyclonal rat anti-Ce-PGL-3 OB0706 (1:10,000, MBL; generated using His-tagged Ce-PGL-3 as the antigen)^15^ or rat polyclonal anti-Ce-TBG-1 (1:100, MBL; generated using His-tagged Ce-TBG-1 as the antigen)^14^ were used as primary antibodies. Goat Alexa Fluor 488 or 568 anti-rat IgG (1:200) (Thermo Fisher Scientific, Waltham, MA, USA) were used as secondary antibodies. DNA was stained with 10 ng/µL DAPI (4′,6-diamidino-2-phenylindole) (Dojindo Laboratories, Kumamoto, Japan).

### Feeding and soaking RNAi

Feeding RNAi in *C. inopinata* was performed according to the protocol established for *C. elegans*^72^. To knock-down GFP in the transgenic lines, the coding region of the GFP transgene was cloned into the feeding plasmid vector L4440 that induces dsRNA expression in *E. coli*. To knock-down the *unc-22* orthologue in *C. inopinata*, genomic fragment of the *C. inopinata unc-22* gene (*Cin-unc-22*) was cloned into L4440. Fifteen adults *C. inopinata* females carrying eggs were placed onto feeding plates seeded with the feeding bacteria (*E. coli* HT115(DE3) transformed with a feeding RNAi vector), and phenotypes in the next generation were analysed on the 5th day. Soaking RNAi in *C. inopinata* was performed according to the protocol established for *C. elegans*^73^. To knock-down the *tbg-1* orthologue in *C. inopinata*, the genomic sequences corresponding to the *C. elegans tbg-1* gene was PCR-amplified using the primers with T7 RNA polymerase primers, and used the amplified fragments as templates for dsRNA synthesis in vitro. As a control, dsRNA for GFP were used. L4 males and females of *C. inopinata* were soaked in the dsRNA solution (~1 mg/mL) for 24 h, and the phenotypes of the progeny of recovered worms were analysed.

### Florescence microscopy

Differential interference contrast (DIC) and fluorescent images were obtained using Zeiss Axioplan 2 imaging microscope (Carl Zeiss, Jena, Germany) with 10×/0.30 NA Plan-NEOFLUAR or ×63/1.20 water Korr UV–VIS–IR objective lenses with Orca-R2 12-bit_16-bit cooled CCD camera (Hamamatsu Photonics, Hamamatsu, Japan), under the control of Metamorph software (Molecular Devices, Sunnyvale, CA, USA). Obtained images were processed by Image J/Fiji software (National Institutes of Health, Bethesda, MD, USA).

### Methods for ascaroside analysis

*Preparation of metabolite extracts*: *C. inopinata* was maintained at 25 °C on NGM agar plates, made with Bacto agar (BD Biosciences) and seeded with *E. coli* OP50 grown overnight. Mixed stage *C. inopinata* worms were rinsed from plates in M9 buffer and allowed to settle to the bottom of a 15 mL conical tube. After settling, the supernatant was removed and worms were resuspended in fresh M9 buffer. This rinsing procedure was repeated for a total of four times. Clean worms were then placed in 100 mL of M9 buffer at a concentration of 1 worm per microliter and incubated on a rotary shaker at 20 °C for 6 h. The cultures were then centrifuged and the resulting supernatant and worm pellet were snap-frozen in liquid nitrogen and stored at −20 °C. The frozen samples were lyophilised and extracted with 95:5 ethanol:water for 12 h. The extract was dried in vacuo, resuspended in methanol and analysed by LC/MS. LC/MS analysis was performed on a Dionex 3000 UPLC coupled to a Thermo Q Exactive high-resolution mass spectrometer. Metabolites were separated using a water–acetonitrile gradient on Agilent Zorbax Eclipse XDB-C18 column, 150 × 2.1 mm, particle size 1.8 µm, maintained at 40 °C. Solvent A: 0.1% formic acid in water, solvent B: 0.1% formic acid in acetonitrile. A/B gradient started at 5% B at 1.5 min after injection and increased linearly to 100% B at 12.5 min. Ascarosides were detected as [M–H]-ions in the negative ionisation mode. Metabolites were identified based on their high-resolution masses (<1 ppm), fragmentation spectra, and comparison of retention times with those of synthetic standards and known metabolites.

*Retention assay protocol*: Standard 5 cm agar plates (made with standard Nematode Growth Medium) were seeded with 100 μL of OP50 *E. coli*. The 16 mm bacterial lawn was grown overnight at 20 °C before being used in trials. Worms were grown at 20 °C on live OP50 *E. coli* on standard NGM agar plates. 9–10-day-old well-fed males were picked from plates containing both males and females onto a new seeded plate the day before experiments (males had no contact with female worms overnight). Using a transparent template to guide spot placement, two 4 mm spots (1 μL) of the relevant solutions were placed on opposite sides of the bacterial lawn and left to dry for several minutes before nematode placement. Recording began immediately upon nematode placement^74^. A drop of the control was placed on one side of the lawn and a drop of the experimental cue was placed on the other side of the lawn. The location of cues was switched throughout the trials, between left/right to minimise bias. A mixture containing the appropriate amounts of the best performing ascarosides (100 fmol Ascr#1, 1 fmol Ascr#3, and 1 pmol Ascr#10) was also tested. 9–10-day-old adult males were isolated the day before being used in trials. All worms were placed at the midpoint between the foci of both scoring regions. Trials were recorded for 20 min and frames were collected at one frame per second. The first 15 min of each video was used for analysis. Results were averaged from 3 to 6 trials. Ten worms were used per assay. We used automated software to compare worm occupancy in each scoring region over time, then adapted the Chemotaxis Index to score preference or avoidance to each ascaroside. Student’s two-tailed *T*-test was used to compare the Chemotaxis Index for each treatment to that of a water control (two spots of water on either side of the lawn). *p < 0.05, **p<0.01, ***p<0.001.

### Data availability

All sequence data from the *C. elegans* sibling genome project have been deposited at DDBJ/ENA/GenBank under BioProject accession PRJDB5687. Taxonomic descriptions were deposited at Zoobank. Nematode specimen and strains are available from T. Kikuchi or N. Kanzaki on request. All relevant data are available from the corresponding authors upon reasonable request.

## Electronic supplementary material

Supplementary Information

Description of Additional Supplementary Files

Supplementary Data 1
