# Supplementary material for: Biology and genome of a newly discovered sibling species of Caenorhabditis elegans
Source: Nat Commun. 2018 Aug 10;9:3216. doi: 10.1038/s41467-018-05712-5 (PMC6086898; doi:10.1038/s41467-018-05712-5)
Supplement: Supplementary file 1 — Supplementary Information [file 41467_2018_5712_MOESM1_ESM.pdf]

Supplementary Materials for

Discovery, biology and genome of *Caenorhabditis inopinata*, a sibling of *C. elegans*

Natsumi Kanzaki, Isheng J. Tsai, Ryusei Tanaka, Vicky L. Hunt, Dang Liu, Kenji Tsuyama, Yasunobu Maeda, Satoshi Namai, Ryohei Kumagai, Alan Tracey, Nancy Holroyd, Stephen R. Doyle, Gavin C. Woodruff, Kazunori Murase, Hiromi Kitazume, Cynthia Chai, Allison Akagi, Oishika Panda, Huei-Mien Ke, Frank C. Schroeder, John Wang, Matthew Berriman, Paul W. Sternberg, Asako Sugimoto, Taisei Kikuchi

correspondence to: asugimoto@m.tohoku.ac.jp, taisei_kikuchi@med.miyazaki-u.ac.jp

This PDF file includes:

Supplementary note 1 - 3

Supplementary Table 1 - 21

Supplementary Figs. S1 to S7

Other Supplementary Materials for this manuscript includes the following:

Supplementary Data 1. Set of reactions used in the metabolic reconstruction of *C. inopinata* (an excel file)

Supplementary notes

Contents

1. Taxonomic description ... 4
2. Biological characteristics of *Caenorhabditis inopinata* ... 8
    - Mating incompatibilities ... 8
    - Growth on NGM agar plate ... 9
    - Sex ratio ... 10
    - Ecological characters ... 10
    - Ascarosides ... 12
3. Genome analysis ... 13
    - Karyotype ... 13
    - Collection of nematode materials for DNA and RNA sequencing ... 13
    - Library preparation and genome sequencing ... 13
    - Optical map ... 14
    - Assembly and manual improvement ... 14
    - Genome re-sequencing of female and male worms. ... 15
    - Repeat analysis ... 16
    - Gene finding ... 18
    - Functional annotation ... 19
        - Assigning protein names and GO terms to predicted proteins ... 19
        - pFAM domain search ... 19
        - CAZymes assignment ... 19
    - Identification of gene families, orthologues and paralogues ... 20
    - Species tree reconstruction ... 20
    - Divergence estimate ... 21
    - Gene family analysis ... 22
    - Conservation of key biological pathways ... 23
    - GPCRs ... 24

Synteny analysis.. 25

Arm/centre dichotomy.. 26

Metabolic pathways 26

Supplementary Refferences 27

Supplementary Figs. ………………………………………………………………………….39

Supplementary Tables ……………………………………………………………………….43

Supplementary note 1. Taxonomic description

Family Rhabditidae Örley

Genus *Caenorhabditis* Osche

Caenorhabditis inopinata n. sp.

Etymology. The biological and phylogenetic characters of this new species were a big surprise for the authors, and thus, the species was named as "*inopinata*" meaning surprising or unexpected in Latin.

Materials examined. Holotype male, four paratype males, and five paratype females were deposited in the USDA Nematode Collection, Beltsville, MD, USA with the accession numbers T-704t (holotype male), T-6923p-6926p (four paratype males) and T-6927p-6931p (five paratype females), and five paratype males and five paratype females are deposited in the Forest Pathology Laboratory Collection of Forestry and Forest Products Research Institute (FFPRI), Tsukuba, Japan with accession numbers, *Caenorhabditis inopinata* MP01-05, FP01-05. Several more unmounted specimens are available upon request from FFPRI (N. Kanzaki). In addition to type materials, temporal water mount materials from cultures were examined for morphological observations. The wild type strain NK74SC is available on request from Miyazaki University (T. Kikuchi) or FFPRI (N. Kanzaki).

Locality. The type specimens of *C. inopinata* n. sp. were collected from the wild type cultured materials (tentative culture code: NK74SC which is the mother strain of inbred line used in the genomic and other analyses in the present study). The culture was established from the figs of a single tree of *Ficus septica* Burm.f. obtained from Ishigaki Island, Okinawa, Japan (GPS: 24º24'38.06" N, 124º11'06.81" E, 71m a.s.l.) on 6 June, 2013. In addition to the type locality, the species has been found from Okinawa, Iriomote, Miyako and Yonaguni Islands, Okinawa, Japan, and Taiwan.

Diagnosis. In addition to generic character, e.g., the structure and arrangement of genital papillae [1], *C. inopinata* n. sp. is characterized by the presence of short stomatal flaps, slender body, short female tail and rounded appendage on anterior cloacal lip and tongue-like postcloacal appendage of males (Figure 1, Supplementary Fig. 1) The combination of these characters has not been reported in the other species in the genus [2-5]. Molecularly, the new species belongs to Elegans group, and it is closest to *C. elegans*, but it is readily distinguished from *C. elegans* by its diagnostic characters listed above. Further, new species can be distinguished from *C. elegans* by its molecular barcode sequences, and clear phylogenetic separation (Supplementary Fig. 2a and 2b).

Remarks. The typological characters of the Elegans group of the genus are mostly identical to each other [2-5]. However, regardless of its phylogenetic closeness to *C. elegans*, *C. inopinata* n. sp. has substantial differences from the other Elegans group species as described above. Characteristic features of *C. inopinata* include a relatively long and slender body, modification of stoma (presence of flap-like extension) and a short female tail. These differences could be because of its adaptation to habitat environment (= inside of fresh fig). Similar differences have been found in other fig-associated nematodes. For example, a long and slender body has been reported in *Teratodiplogaster* and *Bursaphelenchus sycophilus*, i.e., these nematode species are noticeably slenderer than their close relatives, *Allodiplogaster* and *fungivorus* group of *Bursaphelenchus*, respectively [6-9]. Further, characteristic stomatal differences, which are much more prominent than the case of *C. inopinata* n. sp., have been reported in *Teratodiplogaster* which has a large scoop-like extensions of lip sectors, and three *Prisitonchus* species which have five variable morphotypes in each species [9,10]. Although the factor(s) and mechanisms driving the morphological variation are not clarified, these morphological alternations including those of *C. inopinata* n. sp. represent a diverse variation of morphological characteristics in these nematodes.

Description.

Adult. Typological characters are provided as the drawings (Figure 1, Supplementary Fig. 1) and the morphometric values are provided in Supplemental Table 1. Gonochoristic species. Large and slender species compared to the other species in the genus, ca. 1.5-2.5 mm in length, and individuals may reach up 3.0 mm under optimal culturing conditions. Cuticle is moderate to thick because of the large body size with fine transverse annulations and longitudinal striations. Lateral field with four lines. Deirids clearly observed laterally on the lateral field, at the level slightly behind the secretory-excretory pore, but postdeirid was not confirmed by light microscopic observation. Lip region not clearly offset, i.e., not clearly separated from the other parts of body, separated into six lip sectors. Each lip sector has a labial sensillum, and four cephalic sensilla, one on each dorsal and subventral sector. The anterior end of each lip sector very slightly elongated and forming six stomatal flaps. Amphidial apertures small, oval pore-like, visible laterally, slightly dorsally located as in other rhabditid and diplogastrid nematodes, at the level of the margin of cheilo and gymnostom. Tube-like stoma separated into three parts, cheilostom, gymnostom and stegostom from anterior as typical of the rhabditid nematodes. Cheilostom short tube-like occupying ca 1/6-

1/5 of total stoma, posteriorly overlapping with the anterior end of gymnostom. Gymnostom simple tube-like, ca. twice as long as cheilostom, i.e., occupying ca. 1/3 of total stoma or a little more, weakly separated into two subsections, but the separation is difficult to observe in microscopic observation, each subsection is hypothesized to be associated with an arcade synsitium. Stegostom covered by pharyngeal sleeve and separable from gymnostom, separated into four subsections, pro-, meso, meta- and telostegostom. Pro- and mesostegostom not clearly separated, fused to form a simple tube which is almost same as gymnostom in length. Metastegostom forming three flap-like teeth, one on each dorsal, right and left subventral sector, the outer part weakly sclerotized to form a ring surrounding the posterior part of metastegostom. Telostegostom without clear armature, forming small funnel connecting stoma and pharynx (procorpus). Pharynx separated into four sections, procorpus, metacorpus (median bulb), isthmus and basal bulb. Pro- and meta corpus forming muscular anterior pharynx and the other two sections form glandular posterior pharynx. Procorpus muscular tube occupying ca. 60% or a little more of corresponding body diam. Metacorpus forming muscular median bulb without clear valve or glottoid apparatus. Isthmus narrow, not muscular. Basal bulb well-developed with double haustrulum as the glottoid apparatus (grinder-like structure typical to rhabditid nematodes). Pharyngo-intestinal valve (cardia) prominent. Nerve ring around the middle of isthmus. Excretory pore located around the margin of isthmus and basal bulb, perpendicular to body surface and possessing tube-like excretory-secretory duct. A large cell visible on ventral side, at level of, or a little posterior to, cardia, assumed to be a secretory cell associated with secretory-excretory system.

Female. Body straight or slightly ventrally arcuate when killed by heat. Gonadal system didelphic, amphidelphic. Anterior and posterior gonadal system on the right and left of intestine, respectively, and basically symmetric with each other, thus anterior gonad is described from distal part to vulva/vagina. The gonadal system arranged as ovary, oviduct, spermatheca, spermathecal-uterus junction tissue, uterus and vulva/vagina from distal (anterior). Distal part of ovary reflexed dorsally, and often tangled under cultured condition; oocytes arranged in multiple (2-5) rows in the reflexed part, and well-developed oocytes arranged in single row near oviduct. Oviduct short composed with small and rounded cells connecting ovary and spermatheca. Spermatheca composed of large and squared cells forming roundish rectangular-shaped sac. Spermatheca-uterus junction distinctive; a band of long cells with a band of thread-like appearance is surrounded by small and rounded cells. Uterus well-developed often containing many, sometimes more than 20 developing eggs.

Vagina perpendicular to body surface constricted by sphincter muscle at the uterus-vagina junction. Vulva horizontal slit with vulval lips slightly protruding. Tail conical or forming slightly elongated conus with pointed tip. Anus and rectum clearly visible; the intestine-rectum junction constricted with sphincter muscle and surrounded by three (two subventral and one dorsal) rectal glands. Anal opening dome-shaped slit in ventral view. Phasmid forming small pore located laterally at ca. 60% of total tail length from anus.

Male. Testis single-armed on the right subventral of intestine, anteriorly reflexed rightwardly. Spermatocytes arranged in three to four rows in the reflexed part; well-developed spermatocytes in the two to three rows in the middle part; mature spermatids tightly packed in the rest of testis. Vas deferens occupying ca. 1/5 of total gonadal length, composed of large cells, fused with the intestine (rectum) in its posterior end (at the level of spicule) to form a narrow cloacal tube. Tail enveloped by a closed bursa, supported by nine pairs of genital papillae (bursal rays). Anterior cloacal lip with a rounded and sclerotized appendage and bulge-like appendage between rounded appendage and cloacal opening; a small sensilla-like papilla on the bulge-like appendage. Posterior cloacal lip with tongue-like appendage with two cloacal sensilla. Spicules paired, separate, long and slender with evenly slightly ventrally curved blade (calomus-lamina complex) and simply pointed tip. Gubernaculum slender, ventrally arcuate with small squared appendage at the distal end in lateral view; forming spindle shape with outwardly pointed appendage in ventral view. Bursa heart-shaped in ventral view, anteriorly closed with serrated edge; serratae obvious in anterior half and vague in posterior half; terminal notch present but unclear. The nine pairs of genital papillae or bursal rays supporting the bursal velum with an arranged (2/1+1+2+3), i.e., first and second rays (r1 and r2) anterior to cloacal opening, close (stuck) to each other, third ray adcloacal, forth ray slightly posterior to r3, fifth and sixth rays close (stuck) to each other and slightly posterior to r4, seventh to ninth rays (r7-9) grouped at the middle between r6 and tail tip. The r1, r6 and r9 almost reach the edge of velum, others open dorsally or ventrally in the velum. The r1, r5 and r7 opening dorsally forming papilla-form tip, r2, r3, r4, r8 and r9 opening ventrally forming papilla-form tip. The r6 with tube-like dorsal opening, expanded root, and the ray forming bowling pin-shape. Phasmids sensilla-like, around the root of r9.

Supplementary Table 1. Morphometric values of *C. inopinata*

	Caenorhabditis inopinata		
	Holotype male	Paratype males	Paratype females
n	-	9	10
Body length	1358	1348 ± 92 (1221-1448)	1753 ± 132 (1559-2000)
a	24.3	23.9 ± 1.4 (22.3-27.3)	28.3 ± 23.2 (24.8-32.2)
b	4.7	4.9 ± 0.2 (4.6-5.2)	5.7 ± 0.4 (5.3-6.6)
c	21.9	23.3 ± 2.6 (19.2-28.0)	13.7 ± 1.3 (12.6-16.7)
c'	1.7	1.6 ± 0.1 (1.4-1.8)	4.1 ± 0.3 (3.3-4.6)
T or V	71.4	74.5 ± 2.6 (70.8-77.4)	55.4 ± 0.9 (54.0-56.6)
Stomatal opening diam.	4.8	4.8 ± 0.1 (4.7-4.9)	5.0 ± 0.5 (4.1-5.5)
Stomatal depth	22.1	22.4 ± 1.5 (21.4-26.9)	24.7 ± 1.2 (22.1-26.2)
Stomatal depth / diam. ratio	4.6	4.6 ± 0.3 (4.0-5.0)	4.9 ± 0.6 (4.0-5.8)
Anterior pharynx length	148	139 ± 8.0 (125-148)	151 ± 5.5 (143-162)
Posterior pharynx length	122	115 ± 11.5 (94-125)	132 ± 6.4 (120-143)
Anterior / posterior pharynx length ratio	1.2	1.2 ± 0.1 (1.1-1.6)	1.1 ± 0.1 (1.1-1.2)
Mediam bulb diam.	24	24 ± 1.5 (21-27)	25 ± 1.9 (23-29)
Basal bulb diam.	27	29 ± 1.8 (26-32)	32 ± 2.1 (28-35)
Nerve ring from anterior end	195	189 ± 11.5 (163-203)	207 ± 6.6 (195-217)
Nerve ring from median bulb	24	26 ± 6.7 (16-33)	31 ± 5.6 (23-40)
Excreroty pore from anterior end	231	230 ± 9.7 (210-240)	245 ± 11.4 (223-263)
Excreroty pore from mediam bulb	61	68 ± 5.8 (57-75)	68 ± 11.8 (53.9-87.0)
Maximum body diam.	56	57 ± 5.3 (50-65)	63 ± 6.5 (50-71)
Vulval body diam.	-	-	65 ± 6.2 (52-72)
Whole testis length	970	1004 ± 76 (888-1121)	-
Reflexed part of testis	139	169 ± 44 (104-256)	-
Anterior gonad length	-	-	888 ± 77 (793-1069)
Posterior gonad length	-	-	855 ± 78 (776-1034)
Cloacal or anal body diam.	37	37 ± 3.4 (30-41)	31 ± 1.8 (28-34)
Tail length	62	58 ± 5.4 (45-63)	129 ± 10.2 (104-137)
Phasmid position in % of tail length	-	-	45.3 ± 2.5 (41.3-48.3)
Spicule length in chord	46	46 ± 3.7 (39-50)	-
Spicule length curved along median line	48	47 ± 3.8 (40-52)	-
Gubernaculum length in chord	31	31 ± 2.2 (26-33)	-
Gubernaculum length curved along median line	37	36 ± 1.5 (34-38)	-

Supplementary note 2. Biological characteristics of *Caenorhabditis inopinata*

Mating incompatibilities

To confirm that *C. inopinata* and *C. elegans* are reproductively isolated, multiple replicate crosses between the two species were made (Supplementary Table 2). All crosses were performed at 23-25°C using 2 or 5 males and 2 or 3 females (or hermaphrodites); parents were transferred daily. In addition, mating behaviour observations were conducted on limited 2-5 min observation periods for 2-3 times per day until the females died.

For *C. elegans* males crossed to *C. inopinata* females, we observed mating behaviour (scanning for vulva, stereotypical turning behaviour by the males). However, females laid no embryos and received no sperm for all 16 females examined. For the reciprocal cross direction, *C. inopinata* males were tested against three hermaphrodite strains: wild-type N2, *dpy-5*, and *unc-119*. All three crosses produced abundant self progeny broods indicating that *C. inopinata* males do not fertilise *C. elegans*. We also crossed *C. inopinata* males to *fog-2* (*q71*) pseudo-females [11]. Although we observed male mating behaviour, no embryos were

produced and no sperm was transferred (n=21 pseudo-females). In contrast to the lack of cross progeny between the species, control con-specific crosses produced viable F1 and F2 progeny, as expected. Together these results demonstrate reproductive isolation between *C. inopinata* and *C. elegans*.

We also tested 5 other *Caenorhabditis* species (*C. briggsae*, *C. brenneri*, *C. remanei*, *C. guadeloupensis*, and *C.* sp. 50) and found reproductive isolation in all cases (Supplementary Table 2). For four species, mating behaviour was observed or inferred from the presence of mating plugs in at least one cross direction. Of these, the cross between *C. inopinata* males to *C. brenneri* females yielded embryos but none that hatched into larvae.

Growth on NGM agar plate

To find the optimal laboratory conditions for *C. inopinata* culture, we tested *C. inopinata* with the standard *C. elegans* culture method at various temperatures. *C. inopinata* nematodes sterilized with a 1% (v/v) chlorine bleach solution as described before [12], were transferred to nematode growth medium (NGM) plates seeded with *Escherichia coli* strain OP50-1 to establish a pure culture. At 25°C on the standard media, *C. inopinata* grew and multiplied slowly compared to *C. elegans*. *C. inopinata* can multiply at culture temperatures ranging from 15°C to 29°C (Supplementary Fig. 3b), which is much higher than *C. elegans*. This may reflect the subtropical habitats of *C. inopinata.* Dauer-like larvae (Figure 1b) were observed in >14-day old plates incubated at 25°C.

To investigate the food source of *C. inopinata* in nature, we have isolated bacteria from wild nematode bodies as follows: freshly isolated adult nematodes from the syconia were washed 3 times with M9 buffer containing 25 μM tetramisole (Sigma, L9756) followed by washing with an antibiotic solution (50 μg/ml streptomycin, 10 μg/ml chloramphenicol, 100 μg/ml gentamicin in M9 buffer) for 1 hour with vigorous shaking to remove bacteria from the nematode surface [13]. Washed nematodes were rinsed once with M9 buffer and the nematode was crushed by vortex with small gilconia beads (diameter 1 mm) and the solution was spread onto Plate Count Agar (Sigma, #70152) or R-2A Agar (Sigma, #17209) plates. Plates were incubated at 25°C for 48-84 hours and emerged single colonies were randomly isolated using a fine picker. By sequencing nearly-full length 16S ribosomal RNA (rRNA) using primers 27F (5'-AGAGTTTGATCMTGGCTCAG-3') and 1492r (5'-TACCTTGTTACGACTT-3') for 61 colonies, we identified seven different bacterial taxonomic groups (Supplementary Table 4). Seven representative bacterial isolates from four major taxonomic groups were

tested for nematode growth on NGM plate at 25°C. Those isolates except for *Enterobacter* sp. CiN2, showed better nematode growth than *E. coli* OP50-1 (Supplementary Fig. 3a). Worms feeding on *E. coli* strain HT115 showed larger and developed gonads in adults and had larger brood size than those feeding on strain OP50 (Supplementary Table 3), which allowed us to easily perform worm manipulations such as microinjection and microdissection, suggesting strain HT115 might be more suitable for the laboratory culture.

Sex ratio

C. elegans is generally considered a free-living nematode, and mating between males and hermaphrodites produce equal numbers of males and females. In contrast, *C. inopinata* is intimately associated with the fig wasp *Ceratosolen bisulcatus* and colonisation in a fig may be mostly by relatives (please see below, section 2.5). Given this possibility for local mate competition, selection for female biased sex ratios is possible [14]. Thus, we tested if *C. inopinata* might have female biased broods.

To this end, we set up 10 replicate crosses of 2 L4 females and 2 L4 males for *C. inopinata*. We transferred parents to new plates daily, and counted the number of female and male offspring at the adult stage. Summed counts from the 8 successful crosses revealed that *C. inopinata* appears to have a weak female-biased sex ratio (417 females : 359 males; P=0.04 binomial test).

Ecological characters

The fig tree *Ficus septica* occurs throughout southern Japan to Northern Australia [15]. *Caenorhabditis* species were often isolated from rotting fruits, but the nematode *C. inopinata* was found in the fresh fruits (syconia) on the fig stand. We collected fresh syconia in the southern Japanese islands and Taiwan. Figs were cut in half and incubated them on NGM agar plate at 25°C after a brief wash with water. After 3 days of incubation, plates were checked daily and single females were transferred to new NGM plates to establish single-female lines. Nematodes were identified by sequencing partial 18S rRNA gene using primers 988F and 1912R [16]. *C. inopinata* is likely to be widely distributed along *Ficus septica* as we detected the nematode in all the five islands of south Japan (Okinawa, Ishigaki, Miyako, Iriomote and Yonaguni) and Taiwan, where the fig was found (Supplementary Table 5). Regardless of more than 10 years of field surveys of fig-associated nematodes in West Pacific region, i.e., Southern Japan to Northern Australia conducted by the current authors

and other researchers e.g. [9,10,17,18-20], *C. inopinata* has been found exclusively from East Asian *F. septica*, and therefore, the nematode is likely to be associated only with *F. septica* and its mutualistic wasp (see below). However, the detailed host/vector and distribution ranges of *C. inopinata* have not been examined closely, and more systematic field surveys are necessary.

The fig tree *F. septica* has an obligate mutualistic relationship with a small (~2 mm in body length) pollinator wasp *Ceratosolen bisulcatus* (family Agaonidae) (Figure 1c) [21]. Female wasps enter receptive syconia of female trees, where they pollinate female flowers. They also enter syconia of male trees and lay eggs into specialised flowers, where their larvae induce galls and develop. Six to eight weeks later [22], the wasp offspring emerge from their galls into the syconium, just as the male flowers have matured their pollen sacs. The new generation of female wasps leaves the scyonium through holes made by the males and carry pollen to new receptive syconia (Figure 1c) [21]. Because *C. inopinata* was detected only from the fresh syconium interior, we expected that the wasp has a role in moving nematodes from a syconium to a new syconium.

Nematodes were detected with significantly higher frequency in syconia where live wasps or wasp carcasses were found than unfound ones (Fisher's exact test, p= 7.5e-06) (Supplementary Table 6). The numbers of wasp carcasses (i.e. an old generation of wasps) found in a syconium were one or two in most cases. We also detected dauer-like stage *C. inopinata* from the fig wasp that recently escaped from fig syconia. No nematodes were initially visible on the surface of the fig wasp, but after submersion in water nematodes emerged from around the abdomen area. Dauer-like juveniles readily appeared when the insect body was opened. This indicates that some worms were inside the wasp body cavity, which is similar to other fig wasp associated nematodes that are also known to reside in the body cavity, regardless of parasitic (e.g., *Parasitodiplogaster* spp.) or phoretic association (e.g., *Schistonchus* spp.) [23,24]. *C. inopinata* was detected from 89.5% (17/19) of the wasp *C. bisulcatus* escaped from syconia collected in Ishigaki island. The numbers of nematodes on each wasp ranged from 1 to 42 with median 3 (Supplementary Table 7). We also detected *C. inopinata* from the fig-parasitic wasp (*Philotrypesis* sp.), but the frequency was significantly lower than the pollinating wasp (Fisher's exact test, p=4.1e-07) (Supplementary Table 7). In addition, no other insects or animals were found around the fig syconia as frequently as the fig wasps. These results indicate that the wasp *C. bisulcatus* is a main vector for *C. inopinata* to move between syconia. In addition, the small number of nematodes carried by a wasp and

the limited number of wasps entering a syconium (unpublished) suggest *C. inopinata* starts a new colony in a syconium from a small number of nematodes.

In obligatory outbreeding species like *C. brenneri* and *C. remanei*, inbreeding is sometimes accompanied by substantial inbreeding depression [25,26]. However, we did not experience a serious difficulty in establishing an inbred line of *C. inopinata*. This might be because *C. inopinata* natively outcrosses in a wild fig mostly by relatives.

Ascarosides

Caenorhabditis elegans produce a set of secondary metabolites several of which have been shown to serve as social cues including components of the dauer-promoting pheromone and soluble mating attractants [27]. Ascarosides are glycosides of the dideoxy sugar ascarylose and can have various modifications that affect their activity. Since ascarosides are made by many nematodes and each species appears to make a distinct spectrum of ascarosides [28], we tested whether *C. inopinata* makes some key ascarosides, and then tested their attraction to several that they make.

Mixed stage cultures of *C. inopinata* produced the simple ascarosides that are predominant in *C. elegans* cultures including ascr#1, ascr#3, ascr#5, ascr#9, and ascr#10, and low levels of ascarosides modified at the 4' position of the ascarylose sugar core, most notably the indoylated ascaroside icas#9 and the succinylated octopamine derivative osas#9 (Supplementary Fig. 3c). The replicates had similar ratios of these ascarosides.

We tested the response of *C. inopinata* males to these compounds at a range of concentrations from fM to nM and found that they are attracted to ascr#1, ascr#3, and ascr#10 but not ascr#9 (Figure 4c). For ascr#10, *C. inopinata* males exhibit the same concentration preference as *C. elegans* males. For ascr#3, *C. inopinata* males are attracted to fM but *C. elegans* are attracted to pM. *C. elegans* was not tested at the concentrations of ascr#1 that attract *C. inopinata*. We further tested the response of *C. inopinata* males to a mixture of the ascaroside concentrations that produced the three highest CI values. *C. inopinata* showed a preference for the mixture solution comparable in magnitude to its preference for one of the mixture's components, ascr#3 (1 fM). These results provide evidence that the signalling molecules (and perhaps, indirectly, downstream pathways) that facilitate mate recognition are conserved between *C. elegans* and *C. inopinata*.

Supplementary note 3. Genome analysis

Karyotype

Nematodes were fixed with ice-cold methanol and stained with DAPI as previously described [29]. Microscopic observations were carried out using a florescence microscope system (FSX-100, Olympus) and a confocal laser scanning microscope (LSM700, Zeiss). Twelve chromosomes, all similar length, were observed in prophase cells in the two and four-cell stage embryos (Supplementary Fig. 4a), suggesting that the karyotype of *C. inopinata* is 2n = 12. We confirmed in a total of 20 eggs or embryos, all of which had 12 chromosomes, and no specimens showed different compositions of chromosomes.

Collection of nematode materials for DNA and RNA sequencing

The *C. inopinata* reference genome was assembled from genomic DNA obtained from *C. inopinata* inbred line NKZ35 (OMT-10) generated from the original strain NK74SC. Nematodes were cultured on NGM agar plate supplemented with streptomycin (100 µg/ml) and *E. coli* OP50-1 at 25°C for 10 days and harvested using modified Berman funnel [30]. Samples were washed three times with M9 buffer and genomic DNA was extracted using Genomic-tip (Qiagen) following the manufacturer's instructions.

For RNA-seq analyses, age-synchronised worms were grown in S-medium [12] with concentrated *E. coli* OP50-1 at 23°C from eggs collected via bleaching gravid females [12]. Second or third stage larvae (L2/3) were collected from 48 hr culture, and adult females and males were separately obtained from 96 hr culture by hand-picking using a needle. RNA was extracted from ~500 worms (mixed-stage, L2/3, adult female and adult male) using TRI reagent according to the manufacturer's instructions. Total RNA samples were qualified using Experion (BioRad), and only samples with an RNA integrity quality (RIQ) greater than 8.0 were used for library construction.

Library preparation and sequencing

Paired-end sequencing libraries (Supplementary Table 8) were prepared using the TruSeq DNA Sample Prep kit (Illumina) or Nextera DNA Library Prep kit (Illumina) according to the manufacturer's instructions. Multiple mate-paired libraries (3.0, 5.0, 8.0 and 13.0 kb) were constructed using the Nextera Mate-Paired Library Construction kit (Illumina). Libraries were sequenced on the Illumina HiSeq 2000 sequencer using the Illumina TruSeq PE Cluster

kit v3 and TruSeq SBS kit v3 (101 cycles x 2) or the Illumina MiSeq sequencer with the v3 kit (301 cycles x 2) (Supplementary Table 8). The raw sequence data were analyzed using the RTA 1.12.4.2 analysis pipeline and were used for genome assembly after removal of adapter, low quality, and duplicate reads to produce a total of 68 Gb sequence data (Supplementary Table 8).

Ten µg of *C. inopinata* genomic DNA was used to prepare PacBio ~20kb insert size library. A total of 17 SMRT cells were sequenced on a PacBio RSII using P6-C4 chemistry generating 15.2 Gb of data representing 123.6X genome depth of coverage (Supplementary Table 8). Each cell yielded on average 894Mb. The sequence reads all together have a N50 length of 16.5kb and average base quality of 0.83.

One hundred ng of total RNA was used to construct an Illumina sequencing library using the TruSeq RNA-seq Sample Prep kit according to the manufacturer's recommended protocols (Illumina, San Diego, USA). The libraries were sequenced for 101-bp paired-ends on an Illumina HiSeq2000 sequencer using the standard protocol (Illumina).

Optical map

High molecular weight genomic DNA was prepared using CHEF Genomic DNA Plug Kits (BioRad) and then mapped using IRYS Mapping System (BioNano). The fragments were fluorescently stained and visualized to determine fragment sizes. Assembling overlapping fragment patterns of single molecule restriction maps produced an optical map of the genome, which was used to improve the genome assembly of PacBio/Illumina data.

Assembly and manual improvement

Illumina reads from multiple paired-end and mate-pair libraries (Supplementary Table 8) were assembled using the Platanus assembler [31] with the default parameter to produce the v1 assembly.

Two assemblies consisting of PacBio reads were produced separately using Falcon (v.0.3.0) [32] and Canu (v1.3) [33]. Consensus from either assembly was improved using the Quiver module in the SMRT Analysis pipeline (version 2.3.0; http://www.pacbiodevnet.com/SMRT-Analysis/Software/SMRT-Pipe) [34] and merged using Metassembler v3 [35] to produce the v2 assembly.

The two assemblies from the two different technologies (Illumina and PacBio) were merged using Metassembler (v3 assembly). Then, Haplomerger2 [36] was used to remove

haplotypic sequences in the assembly and contigs were further scaffolded using Illumina mate-pair reads using SSPACE [37] and manually curated using gap5 (v4 assembly) [38]. Base correction was performed with 8.8 Gb Illumina pair-end reads using ICORN2 [39] with 5 iterations to produce the v5 assembly.

The v5 genome assembly was further improved by optical mapping (see above). The optical map consisted of 239 contigs, an assembled size of 141.7 Mb and approximately 78x genome coverage of optical data. The optical map data were used to order and orientate sequence scaffolds, to measure the size of sequence gaps, and independently validate the sequence assembly, producing genome assembly v7.

To assess the completeness of the assemblies we used CEGMA v235 [40] and BUSCO [41]. CEGMA and BUSCO report the percentage of highly conserved eukaryotic gene families that are present as full or partial genes in the assembly. For most eukaryotes, 100% (or nearly 100%) of core gene families represented by a full gene in the genome would be expected. Thus, these provide a measure of the completeness of the assembly for a species. The *C. inopinata* assembly (v7) showed high completeness (Table 1, Supplementary Table 9), comparable to *C. elegans* and *C. briggsae.* The average number of CEGs (hits for the 248 single-copy eukaryotic core genes) for *C. inopinata* is 1.16, which is similar to *C. elegans* and *C. briggsae* (Supplementary Table 9), indicating that heterozygosity in the genome assembly is low. Heterozygous SNPs and indels detected by remapping of the Illumina short reads to the reference genome suggest that ~0.5% nucleotide positions of the reference genome still carry residual heterozygosity in the inbred line.

Genome re-sequencing of female and male worms.

DNA was extracted from male and immature (virgin) female worms using QIAamp DNA mini kit, following manufacturer's instructions. Illumina paired-end libraries (400 bp fragment) were prepared and sequenced using MiSeq (Supplementary Table 8) following the protocol above as for genome sequencing. By analysing sequence data from male or female worms, we identified an 18.2 Mbp scaffold with differing coverage between the sexes (Supplementary Fig. 4b). The median depth of coverage of male sequence data for this scaffold was approximately 50% compared with the rest of the genome, while the same scaffold had a coverage of 100% using data from immature females (Supplementary Fig. 4b). We propose that this scaffold represents the X-chromosome. We didn't observe regions that are potentially Y chromosome specific sequences (i.e., highly heterozygous in females).

Repeat analysis

Repeats within the assemblies were identified using the combined outputs of RepeatModeler (http://www.repeatmasker.org/RepeatModeler.html) and TransposonPSI (http://transposonpsi.sourceforge.net). For each species, UCLUST was used to cluster repeat sequences from RepeatModeler and TransposonPSI that had ≥80% identity, to generate consensus sequences for a non-redundant repeat library. RepeatMasker (v.3.2.8, http://www.repeatmasker.org) was then run (using the slow search option) with a custom repeat library for each species, to calculate the distribution of each repeat and its abundance in the genome. About 29.53% of the *C. inopinata* genome was detected as repeats, which is higher than *C. elegans* (17.26%) and *C. briggsae* (27.38%). For most repeat categories, *C. inopinata* exceeded *C. elegans* in number of elements (Supplementary Table 10). In particular, LTR retrotransposons, long interspersed nuclear elements (LINES) and Tc1/mariner transposons are highly expanded in the *C. inopinata* genome compared to *C. elegans* and *C. briggsae* (Figure 5a, Supplementary Table 10).

To identify long terminal repeat (LTR) retrotransposable elements, we used a combination of LTRharvest/LTRdigest [42,43] and MGEScan-LTR [44]. LTRharvest was used to identify potential candidates of LTRs in the genome (LTR lengths 100-1000bp, total length 1-10kbp, 85% minimum LTR similarity, TSD length 4-20bp, seed 30bp, looking for motif starting 'tg' and ending in 'ca'). LTRdigest was used to subsequently annotate the candidates with protein domains from Pfam [45] and GyDB [46] as well as putative primer binding sites and polypurine tracts. MGEscan-LTR was also run on the genome to identify LTRs de novo. The two results were processed using custom scripts to remove low confidence candidates by only keeping (1: Full) candidates with full LTR retrotransposon domain sets (reverse transcriptase, protease, integrase, RNase H), (2: Partial) candidates with at least one protein domain (e-value < 1e-20) but not full, and (3: LTR-only) candidates with only LTR regions but having high similarity (>95%) to LTRs from (1) or (2).

We have identified highly expanded LTR elements in *C. inopinata* compared to *C. elegans* and *C. briggsae* (Figure 5a, Supplementary Table 11). Phylogenetic analyses using RT domains revealed that expansion in *C. inopinata* occurred in two LTR families (Bel/Pao and Ty3/Gypsy) and an unclassified clade (RVT_1). The three clades contained high numbers of *C. inopinata* sequences (113, 66 and 93, respectively) and a small number of *C. elegans* and *C. briggsae* sequences (8, 7 and 20 for *C. elegans* and 3, 9 and 4 for *C. briggsae*,

respectively). Further, within Bel/Pao and Ty3/Gypsy families *C. inopinata* sequences grouped into subclades and all subclades contained a small number of sequences from *C. elegans* and *C. briggsae* with only an exception of a small subclade near the Micropia/Mdg3 containing two sequences only from *C. inopinata*. This suggests that the expansion in *C. inopinata* is likely not a result of new acquisitions of LTR elements from other sources but because of expansions of existing elements possibly due to a function loss to suppress transposon activities or a function gain to accelerate it (see below).

A high number of DNA repeat elements was also found in the *C. inopinata* genome compared to *C. elegans* (Supplementary Table 11). Whereas most *C. elegans* DNA transposons are fossil remnants that are no longer mobile [47], *C. inopinata* seems to possess many active DNA transposons in the genome. We found a total of 702 gene models (see below) that encode intact transposases in the *C. inopinata* genome. All of them seem to be Tc1/mariner type (TcMar) transposases. The expanded TcMar transposase family in *C. inopinata* comprise 8.85 % of the genome, compared with 1.31 and 3.04 % of the *C. elegans* and *C. briggsae* genomes, respectively (Supplementary Table 10). Phylogenetic analyses show that the *C. inopinata* TcMar transposases form five main clusters which are related to *C. elegans* Tc1 and Tc3 transposons, *C. briggsae* Tcb1 and Tcb2 transposons, Mos1 transposons from *Drosophila* and HsMar transposons from humans. One smaller cluster of *C. inopinata* TcMar transposases was distinctly different from the *Caenorhabditis* spp. *D. melanogaster* and human transposases included in the phylogenetic analysis (Supplementary Fig. 5). Other transposon families, such as TcMar-Tc2, hAT and MULE-MuDR were not expanded in *C. inopinata,* when compared to *C. elegans* and *C. inopinata*. An expansion of LINES was also observed in the *C. inopinata* genome; 1.63% of the genome comprise LINES, compared with 0.49 and 0.52 % of the *C. elegans* and *C. briggsae* genomes, respectively. This is predominantly due to an increase in RTE (retrotransposable element) LINES which comprise 1.27 % of the *C. inopinata* genome compared with only 0.02%, or no, RTEs present in *C. elegans* and *C. briggsae* genomes, respectively (Supplementary Table 10).

The distribution of LTR elements and DNA repeat elements has association with genome rearrangements as well as lost genes or unique genes in *C. inopinata*. By looking at synteny between *C. inopinata* and *C. elegans*, we found synteny breaks in the *C. inopinata* genome were enriched in transposon-related genes (see Synteny analysis). In particular, a region of chromosome V analogous to regions in *C. elegans* and *C. briggsae* where the

argonaute protein-coding gene *ergo-1* is located, gene order has undergone rearrangement and gene loss including loss of orthologues of *ergo-1* and *k10c9.1/ cbg17878* which codes for a protein of unknown function. In this region two LTR elements and one TcMar transposase are present (Figure 5c), suggesting high LTR retrotransposon and/or DNA transposase activities could be a driving force of the gene loss in this region. Expansion of DNA transposons and LTR elements in the *C. inopinata* genome could therefore contribute to evolutionary divergence between *C. inopinata* and *C. elegans*.

Gene finding

To predict protein-coding genes, Augustus (v. 3.0.1) [48] was trained for *C. inopinata* based on a training set of 1500 non-overlapping, non-homologous and manually curated genes from the initial gene predictions based on CEGMA core genes [40] and Augustus *C. elegans* parameters. A selection of gene models was curated in Artemis [49] using aligned RNA-seq data and BLAST [50] matches against the NCBI database. RNA-seq reads were mapped to the genomes using Hisat2 [51] (parameters: --rna-strandness RF --min-intronlen 20 –max-intronlen 10000). Based on the Hisat2 alignments, the bam2hints program (part of the Augustus package) was used to create the intron hints, with minimum length set to 20 bp. The mapped RNA-seq reads were also assembled into transcripts using Cufflinks [52] with minimum intron length of 20 bp. The predicted exons in the resultant set of transcripts were used as the exonpart hints.

The trained versions of Augustus were run using all the hints for that species as input. Introns starting with ‘AT’ and ending with ‘AC’ were allowed (--allow_hinted_splicesites=atac). A weight of 10^5 was given to intron and exonpart hints from RNA-seq. The minimum intron length was set to 15 bp. If Augustus predicted multiple, alternatively spliced transcripts for a gene, we only kept the transcript corresponding to the longest predicted protein yielding a total of 20,976 gene models (v7.7 gene set).

Gene structures in the Augustus predictions were refined by manual curation in Artemis, using RNA-seq mapping results and BLASTP [50] results against *C. elegans* proteins. In this manual curation process, we targeted mainly on erroneously fused gene models (v7.9 gene set). We further curated genes coding 7TM-GPCRs intensively using TBLASTN [50] with *C. elegans* GPCRs as queries to get the v7.10 gene set. If a gene was manually curated, it replaced the original Augustus gene prediction in the gene set and we finally obtained 21,609 gene models (Table 1).

Functional annotation

Assigning protein names and GO terms to predicted proteins.

Unique names were assigned to each predicted protein, following UniProt's protein naming guidelines (http://www.uniprot.org/docs/nameprot), where possible. One-to-one or many-to-one (e.g. many-*C. inopinata* to one-*C. elegans*) orthologues were first identified based on phylogenetic trees from the OrthoFinder analyses (see below), together with 7 other *Caenorhabditis* species and UniProt protein names and locus names of the *C. elegans* orthologues were transferred to the *C. inopinata* genes.

Gene ontology (GO) terms were also assigned to genes by transferring GO terms from *C. elegans* orthologues. Manually curated GO annotations were downloaded from the GO Consortium website and transferred to the *C. inopinata* predicted proteins based on orthologue information identified by phylogenetic trees in OrthoFinder groups (see below). GO terms of the three possible types (molecular function, cellular component and biological process) were assigned to predicted proteins in this way. Additional GO terms were identified using Blast2Go [53] with BLAST [50] searches against the NCBI nr database and InterProScan [54] results.

pFAM domain search

The latest version Pfam search (ver. 30.0) [45] with HMMER3 was performed for *C. inopinata* (v7.10), *C. elegans* (wormbase 252) and *C. briggsae* (wormbase 252) gene sets.

The Top 50 highly represented Pfam domains in *C. inopinata*, *C. elegans* and *C. briggsae* are shown in Supplementary Table 12. Highly represented domains in *C. inopinata* included many retrotransposon-related domains (rve, Peptidase_A17, HTHs and RVT_1) as well as DNA transposon-related domains (DDE, Transposase_1). On the other hand, numbers of receptor related domains (7TM_GPCRs, Hormone_recep and Recep_L_domain) were remarkably low in *C. inopinata* compared to *C. elegans*.

CAZymes assignment

Carbohydrate-active enzymes (CAZymes) are involved in the metabolism of glycoconjugates, oligosaccharides and polysaccharides. Identifying and comparing CAZymes (CAZyme size and diversity) can provide information for better understanding of their nutritional strategy or host specificity [55].

We detected CAZymes using the dbCAN database [56] with HMMER3 with e-value thresholds (1e-13 for >80aa, 1e-9 for everything else) from the gene sets of *C. inopinata*, *C. elegans* and *C. briggsae*. A phylogenetic tree of the genes in each CAZyme family was generated and if necessary further categorisation was performed based on the tree.

Almost identical repertories of CAZyme families were found in *C. inopinata* compared to those found in *C. elegans* and *C. briggsae* (Supplementary Table 13). In *C. inopinata,* 23 families of glycoside hydrolases (GH) were identified, but no enzymes that were related to degradation of plant or fungal cell walls were detected. Other enzyme classes including auxiliary activities (AA), carbohydrate esterases (CE) and carbohydrate-binding modules also have very similar repertories among the three species. No pectate lyases were found in all three genomes. These results suggest *C. inopinata* uses similar food sources and carbometabolic pathways as *C. elegans* and *C. briggsae*. A total of 193 glycosyltransferases (GT) which belong to a wide range of GT classes (40 families) were identified in *C. inopinata*. Although *C. elegans* has higher number of glycosyltransferases (285) than *C. inopinata,* GT family repertories were identical to each other.

Identification of gene families, orthologues and paralogues

To establish orthology relationships among *Caenorhabditis* species, non-redundant proteomes (protein sets containing only longest isoforms) of eight *Caenorhabditis* species and an outgroup species were obtained from WormBase (version WS255). These species include *C. elegans, C. briggsae, C. angaria, C. brenneri, C. japonica, C. remanei, C. sinica, C. tropicalis* and *P. pacificus*. We used OrthoFinder (v0.2.8) [57] with default options to assign the orthology.

A total of 264,976 genes from 10 species were assigned into 16,861 orthologue groups with a median size of 10. Number of orthologue groups with all species present, single-copy orthologue groups and species-specific orthologue groups were 5300, 1016 and 541, respectively (Figure 2a).

Species tree reconstruction

From the OrthoFinder results, 1,016 gene families were identified that contain single gene from each species (single-copy orthologues). The proteins in each single-copy family were aligned using MAFFT version v7.221 [58], poorly-aligned regions were trimmed using GBlocks v0.91b [59], and then the 1000 trimmed alignments (all sequences in 16 alignments

were removed in the Gblocks trimming) were concatenated. A maximum likelihood phylogenetic tree was produced based on the concatenated alignment, with each protein alignment an independent partition of these data, applying the best-fitting substitution model identified using the RAxML option (-m PROTGAMMAAUTO). This inference used RAxML v8.2.7 [60] with 10 random addition-sequence replicates and 500 bootstrap replicates, and otherwise default heuristic search settings.

The resulting maximum likelihood tree showed an identical topology as trees inferred by 18S rRNA gene and by 11 conserved genes (genes for SSU and LSU rRNA, orthologues of C. elegans *ama-1* (the largest subunit of RNA polymerase II), *lin-44* (encoding a Wnt signaling factor), *par-6* (encoding a PDZ-domain-containing protein), *pkc-3* (encoding an atypical protein kinase C), ZK686.3 (orthologous to the putative tumor suppressor N33), W02B12.9 (orthologous to the mitochondrial carrier protein MRS3/4), ZK795.3 (orthologous to a U3 small nucleolar ribonucleoprotein component), Y97E10AL.2 (a predicted alpha/beta hydrolase) and Y45G12B.2a (a predicted E3 ubiquitin ligase)) (Supplementary Fig. 2a and 2b), placing *C. inopinata* at the sister position of *C. elegans*.

Divergence estimate

We estimated neutral divergence between *C. inopinata* and *C. elegans* following Cutter's method of divergence time estimation [61,62]. To begin with the estimation, we computed lineage-specific rate of synonymous (dS) and non-synonymous (dN) substitution for orthologous groups of genes (see above) between each of *Caenorhabditis* species in the phylogeny using Codeml in PAML (v4.9) [63] with options (runmode = 0, CodonFreq = 2, model = 1, fix_omega = 0, omega = 0.01). Lineage ages (T) were inferred by applying median of synonymous-site divergence values and direct measures of the average per-site mutation rate in *C. elegans* ($\mu = 9.0 \times 10^{-9}$ mutations per generation) [64] to the equation from the neutral theory of molecular evolution $T = dS/\mu$. Since there would be saturation in dS among species, effective codon usage numbers (Nc) was calculated by DAMBE (v6) [65] and used to adjust the divergence values upward by transforming least-squares regression of branch-specific dS to the expected dS for the Nc. In addition, we removed extreme values (dN > 0.5, dS > 5 and dS < 0.0005) to avoid biases from saturation of synonymous rate between distantly related species and miss assignment of orthologous groups.

For *C. inopinata*, the median selection-adjusted, lineage-specific divergence can be translated to an estimated common ancestry date of 142.73 million generations ago, while it

is 127.76 million generations ago in *C. elegans* (Supplementary Fig. 4c). If following on Cutter's method [61,62] to put a 60-day average generation time (~6 generations per year), it will yield separation times of 23.46 million years ago and 21.00 million years ago for *C. inopinata* and *C. elegans,* respectively, from their most recent common ancestor (TMRCA) (Supplementary Fig. 4c). Considering that the vector wasp of *C. inopinata* has a 6-8 weeks generation time [22] and *C. inopinata* should have at least 2 generations per wasp generation according to our observations of non dauer-like 3rd stage larvae in syconia, the average generation time is likely less than 30 days making the separation time <12 million years.

Gene family analysis

Genes in each *Caenorhabditis* species were categorised based on their relationships in orthologous groups: i) Conserved one-one; one-many; many-many present in all species, ii) *Caenorhabditis* specific, iii) Elegans super-group-specific, iv) *C. elegans* and *C. inopinata* specific, v) members of species-specific gene family, and v) species-specific singletons. Genes that did not fit any of the above categories were categorised as "other". Compared to other species in the Elegans super-group, *C. inopinata* and *C. elegans* seem to have a smaller number of genes (Figure 2a). Genes belonging to *C. elegans* and *C. inopinata* clade-specific orthology occupy only ~0.3% of their total genes (Figure 2a), suggesting most of the genes are either conserved across all species or amongst *Caenorhabditis*.

To estimate branch or lineage specific gain and loss of orthologous gene families, we used CAFÉ (v3) [66] with gene family results from OrthoFinder and ultrametric phylogenetic tree using divergence values calculated above as inputs under parameters "-p 0.01, -r 1000". CAFE identified 198 gene families (with a total of 2349 genes) with significantly higher-than-expected rate of gains/losses in the *C. inopinata* lineage ($P \leq 0.01$, Supplementary Table 14). These significantly-expanded gene families were enriched in GO terms associated with transposons, whereas significantly contracted gene families were enriched in GO terms related to signalling receptions (Supplementary Table 14). The GO term "G-protein coupled olfactory receptor activity" was detected both in expanded and contracted families. Most of the species-specific lineage gene gains are singleton genes (Figure 2a). Interestingly, there is greater gene loss in *C. inopinata* (2,993) compared to that in *C. elegans* (1,403) (Figure 2a). In conclusion, the number of genes is smaller in the *C. inopinata* and *C. elegans* clade, and can be linked to higher levels of gene loss in this clade than the other *Caenorhabditis*

clades. Moreover, partial conserved ('other genes' category) accounts for the major difference in gene number between *C. inopinata* and *C. elegans.*

Conservation of key biological pathways

To see conservation/difference of key biological pathways in *C. inopinata*, *C. elegans* and *C. briggsae*, we looked closely at orthologues involved in well-studied biological pathways of interest, including Ins/IGF-1 signalling, dauer formation, sex determination, and small RNA pathways (Supplementary Table 15 - 19).

Most of those orthologues were identified in *C. inopinata* in a one-to-one manner with *C. elegans* and *C. briggsae*, suggesting conservation of key biological genes in the group, and also supporting the high quality of gene predictions in *C. inopinata*. Some exceptions were, however, found in each pathway. For examples, in the Ins/IGF-1 signalling pathway, we found serine/threonine kinase Akt/PKB genes are duplicated in *C. elegans* (*akt-1* and *akt-2*) and one of two 14-3-3 proteins in *C. elegans* (encoded by *par-5/ftt-1* and *ftt-2*) is missing in *C. inopinata*, whereas two copies of the *daf-18* orthologue, which exist as a single copy in *C. elegans*, were found in *C. inopinata*.

Interestingly we found a loss of *ergo-1*, *eri-6/7* and *eri-9* orthologues in *C. inopinata.* The gene loss was confirmed by orthofamiliy analysis, BLASTp/tBLASTn search against the *C. inopinata* genome and gene models using the *C. elegans* sequences as queries. No similar sequences were found in the transcriptome data either. These three genes all code for proteins involved in the ergo-1 siRNA pathway in *C. elegans.* In comparison, other siRNA pathways, e.g., the ALG3/4 pathway, are highly conserved among the three *Caenorhabditis* species (Supplementary Table 19). ERGO-1 forms part of the ERGO-1 Argonaute complex that binds to 26G siRNAs, particularly in the female germline where 26G RNAs are enriched in oocytes and embryos through to early larval development [67]. ERGO-1 class 26G-RNAs likely involved in targeting and suppressing the expression of newly acquired, duplicated genes, possibly of viral origin [68] and potentially deleterious non-coding regions of the genome [69]. The loss of *ergo-1* in *C. inopinata* may therefore be a factor in the expansion gene families observed in the *C. inopinata* genome, for example the expansion of DNA transposon families (Supplementary Table 11). The ERGO-1 protein is closely related to the piwi-related Argonautes PRG-1 and PRG-2 (piRNA pathway). These Argonaute proteins bind to 21U RNAs which predominantly target transposons and thus have a role in transposon regulation [70]. In common with the piRNA pathway, the ERGO-1 26G-RNA pathway also trigger the

production, and subsequent binding of 22G-RNAs, to WAGO-type Argonaute proteins. The ERGO-1 Argonaute is less well characterised compared to piwi-related Argonautes in nematodes and more research is required to establish if ERGO-1 is also specifically involved in transposon regulation, as well as gene family expansion more generally. Furthermore, in *C. elegans*, *mut-1* is an essential component of the ERGO-1, but not ALG3/4, 26G siRNA pathways. In *mut-1* mutants the expression of ERGO-1 is suppressed and this is coupled with a suppressed desilencing of transposable elements [71]. It is also of interest to note that mariner-like DNA transposon genes can be found in the same region of the *C. inopinata* genome that *ergo-1* was lost, and where LTR elements are also present (Figure 5c). Compared with the *ergo-1* region of the genome which has undergone rearrangement in *C. inopinata*, the region that corresponds to the *eri-9* region shows greater gene synteny with *C. elegans* and *C. briggsae*. In *C. elegans* the *eri-6* and *eri-7* genes are located in the same region of the genome, but in *C. inopinata* this region is located in two distinct regions of chromosome I, more similar to the gene synteny observed for *C. briggsae* (Data not shown).

GPCRs

Chemoreception of environmental stimuli is a major sensory system in small soil nematodes like *C. elegans* [72,73]. Chemoreception is mediated in *C. elegans* by members of the seven-transmembrane G-protein-coupled receptor class (7TM GPCRs). Those receptors in *C. elegans* are specifically called “serpentines” and comprise approximately 1300 apparently intact genes with ~400 pseudogenes [73]. In the Pfam domain analyses, we found domains related to 7TM were significantly contracted in *C. inopinata* compared to *C. elegans* (see above, Supplementary Table 12). We manually curated 7TM-GPCR genes in the *C. inopinata* genome based on TBLATN searches using *C. elegans* serpentines as queries, and identified a full set of serpentine-like genes in *C. inopinata* (430 genes including 48 partial or pseudo genes) (Supplementary Table 20). *C. inopinata* has smaller numbers of genes in all serpentine families (except *odr*) than *C. elegans*. To see the detailed relationships within *Caenorhabditis*, phylogenetic trees of genes in each serpentine family from *C. inopinata*, *C. elegans* and *C. briggsae* were constructed using Mafft v.7.221 [58] and FastTree 2.1.8 [74]. It has been reported that *C. elegans* has highly expanded serpentine families and the gene number differences between *C. elegans* and other *Caenorhabditis* species were the result of gene expansions in *C. elegans* rather than gene losses in other species [72,73]. Our phylogenetic analyses, however, further revealed that massive gene losses clearly occurred

in *C. inopinata,* as we found many one-to-one or one-to-many clusters of *C. elegans* and *C. briggsae* that do not contain *C. inopinata* genes in the serpentine family trees (e.g. srd, srh, sre and str) (Figure 4b). We also found local expansions of *C. inopinata* serpentine genes in those trees, which retain *C. inopinata* serpentine gene number as high as that of *C. briggsae* (Figure 4b, Supplementary Fig. 6). This may reflect the *C. inopinata* life style in which the nematode doesn't require a wide variety of receptors to detect environments like *C. elegans* or other soil nematodes because of its limited habitat area (inside fig fruit) and the characteristic lifecycle in which a fine detection for specific types of chemical stimuli may be required, probably due to the close interaction with the fig and the vector insect (Figure 1c).

Synteny analysis

Gene synteny was inferred between *C. inopinata*, *C. elegans* and *C. briggsae* using DAGchainer [75] based on positions of orthologous genes. Synteny-linking plots were generated using in R and Circos (v0.69-4) [76]. *C. briggsae* scaffolds on Chr.II, Chr.III and Chr.IV were reverse complemented to illustrate the most parsimonious scenario. Gene collinearity is largely conserved and frequently rearranged within chromosomes among the three *Caenorhabditis* genomes (Supplementary Fig. 7a). The frequencies of rearrangements on autosomes appear to be independent of species relatedness as well as arm/center definition (Figure 2b, Supplementary Fig. 7a). Between *C. inopinata* and *C. elegans*, only few inter-chromosomal rearrangements were detected (Figure 2b, Supplementary Fig. 7a). 76% of the *C. inopinata* genome can be assigned to blocks of collinear genes (synteny), while ~75% for *C. elegans* genome (Table 1). Regions without synteny (synteny breaks) are usually genes that are too diverged to establish orthologous relationship, have undergone expansion or loss, rearrangements and species-specific genes. Consistent with gene family analysis, synteny break regions in *C. inopinata* genome are due to expansion of gene family enriched in transposon-related GO terms as well as fucosylation and hermaphrodite germ line sex determination (Supplementary Table 21). Conversely, synteny breaks in *C. elegans* are mainly due to an expansion of G protein-coupled receptors (GPCRs) gene family especially on chromosome V (Supplementary Table 21).

To investigate why *C. inopinata* is 20 Mb larger compared to *C. elegans* despite similar coding content (Table 1, Supplementary Table 9), we looked at the distribution of syntenic block size in both species. We found that longer intergenic spacing is mainly responsible for larger block size on chromosomes of *C. inopinata* (Supplementary Fig. 7b). Intron sizes are

slightly larger in *C. inopinata* except chromosome IV and X (Supplementary Fig. 7b). Chromosome X contains some of the most conserved and largest blocks and fewer rearrangements (Supplementary Fig. 7a, 7d). Conversely, chromosome V contain species-specific gene families which led to synteny breaks (Supplementary Fig. 7a, 7d).

Arm/centre dichotomy

It has been shown that features such as tandem repeats and conserved genes are distributed unequally across the chromosomes in *Caenorhabditis* species due to varying recombination rates between arm and centre regions [77,78]. The completeness of the *C. inopinata* genome allowed us to revisit the differences of the chromosome arm/centre that appear to be a hallmark of *Caenorhabditis* genome evolution.

Center and arm regions were defined according to Ross *et al* [79] in *C. elegans* and the distribution of tandem repeats in *C. inopinata* as tandem repeats have been shown correlated with crossover rate in *C. elegans* genome [78]. We found that these features were condensed in autosomal arm regions, which is consistent with other *Caenorhabditis* genomes (Supplementary Fig. 7c) [77,78]. Synonymous substitution rates (dS) between *C. elegans* and *C. inopinata* orthologues at autosomal arm regions are significantly higher than those from center regions (p=1.15e-13), while no differences were found between both regions of chromosome X (p=0.7662) and are lower than their autosomal counterpart (p=3.368e-11) (Supplementary Fig. 7e). Interestingly, conserved single copy genes are predominantly found on chromosome X (Supplementary Fig. 7d). As a result, the arm/center dichotomy is apparent in the genome of C. *inopinata*, but conserved genes are dispersed across autosomal arms and centers.

Metabolic pathways

An initial set of EC predictions was obtained from several methods: DETECT v2.0 [80] (cutoff ILS ≥0.9, ≥5 positive hits100), PRIAM [81], KAAS [82] and EC number transfer from WormFlux [83] based on Orthologue information from OrthoFinder (See above). From these assignments, a set of high-confidence predictions was derived. Reaction assignments to metabolic pathways and pathway hole filling were performed using Pathway Tools v18.0 [84]. Those predictions with support from either PRIAM [81] or BLASTP [50] were used to augment the high confidence set of predictions. We identified all the known metabolic pathways of *C. elegans* [83] and no extra pathways in *C. inopinata* (Supplementary Data 1).

Supplementary References

1 Sudhaus, W. Phylogenetic systematisation and catalogue of paraphyletic" Rhabditidae"(Secernentea, Nematoda). *Journal of nematode morphology and systematics* **14**, 113-178 (2011).

2 Sudhaus, W. & Kiontke, K. Phylogeny of Rhabditis subgenus Caenorhabditis (Rhabditidae, Nematoda). *Journal of Zoological Systematics and Evolutionary Research* **34**, 217-233 (1996).

3 Kiontke, K. C. *et al.* A phylogeny and molecular barcodes for Caenorhabditis, with numerous new species from rotting fruits. *BMC Evol Biol* **11**, 339, doi:10.1186/1471-2148-11-339 (2011).

4 Huang, R. E., Ren, X., Qiu, Y. & Zhao, Z. Description of Caenorhabditis sinica sp. n. (Nematoda: Rhabditidae), a nematode species used in comparative biology for C. elegans. *PLoS One* **9**, e110957, doi:10.1371/journal.pone.0110957 (2014).

5 Felix, M. A., Braendle, C. & Cutter, A. D. A streamlined system for species diagnosis in Caenorhabditis (Nematoda: Rhabditidae) with name designations for 15 distinct biological species. *PLoS One* **9**, e94723, doi:10.1371/journal.pone.0094723 (2014).

6 Kanzaki, N., Aikawa, T., Maehara, N. & Thu, P. Q. Bursaphelenchus kesiyae n. sp.(Nematoda: Aphelenchoididae), isolated from dead wood of Pinus kesiya Royle ex Gordon (Pinaceae) from Vietnam, with proposal of new subgroups in the B. fungivorus group. *Nematology* **18**, 133-146 (2016).

7 Kanzaki, N., Ragsdale, E. J. & Giblin-Davis, R. M. Revision of the paraphyletic genus Koerneria Meyl, 1960 and resurrection of two other genera of Diplogastridae (Nematoda). *ZooKeys*, 17 (2014).

8 Kanzaki, N., Tanaka, R., Giblin-Davis, R. M. & Davies, K. A. New plant-parasitic nematode from the mostly mycophagous genus Bursaphelenchus discovered inside figs in Japan. *PloS one* **9**, e99241 (2014).

9 Kanzaki, N. *et al.* Teratodiplogaster fignewmani gen. nov., sp. nov.(Nematoda: Diplogastridae) from the syconia of Ficus racemose in Australia. *Zoolog. Sci.* **26**, 569-578 (2009).

10 Susoy, V. *et al.* Large-scale diversification without genetic isolation in nematode symbionts of figs. *Science advances* **2**, e1501031 (2016).

11 Schedl, T. & Kimble, J. fog-2, a germ-line-specific sex determination gene required for hermaphrodite spermatogenesis in Caenorhabditis elegans. *Genetics* **119**, 43-61 (1988).

12 Stiernagle, T. Maintenance of C. elegans. *WormBook*, 1-11, doi:10.1895/wormbook.1.101.1 (2006).

13 Diaz, S. A. & Restif, O. Spread and transmission of bacterial pathogens in experimental populations of the nematode Caenorhabditis elegans. *Appl. Environ. Microbiol.* **80**, 5411-5418, doi:10.1128/aem.01037-14 (2014).

14 Hamilton, W. D. Extraordinary sex ratios. A sex-ratio theory for sex linkage and inbreeding has new implications in cytogenetics and entomology. *Science* **156**, 477-488 (1967).

15 Corner, E. J. H. & Gardens, B. *Check-list of Ficus in Asia and Australasia with keys to identification*. (Botanic Gardens Singapore, 1965).

16 Holterman, M. *et al.* Phylum-wide analysis of SSU rDNA reveals deep phylogenetic relationships among nematodes and accelerated evolution toward crown Clades. *Mol. Biol. Evol.* **23**, 1792-1800, doi:msl044 [pii]
10.1093/molbev/msl044 [doi] (2006).

17 Zeng, Y., Giblin-Davis, R. M. & Ye, W. Two new species of Schistonchus (Nematoda: Aphelenchoididae) associated with Ficus hispida in China. *Nematology* **9**, 169-187 (2007).

18 Sriwati, R., Takeuchi-Kaneko, Y., Jauharlina, J. & Kanzaki, N. Aphelenchoidid nematodes associated with two dominant Ficus species in Aceh, Indonesia. *Nematology* **19**, 323-331 (2017).

19 Kanzaki, N., Woodruff, G. C. & Tanaka, R. Teratodiplogaster variegatae n. sp.(Nematoda: Diplogastridae) isolated from the syconia of Ficus variegata Blume on Ishigaki Island, Okinawa, Japan. *Nematology* **16**, 1153-1166 (2014).

20 Davies, K. A., Bartholomaeus, F., Kanzaki, N., Ye, W. & Giblin-Davis, R. M. Three new species of Schistonchus (Aphelenchoididae) from the Ficus subgenus Sycomorus (Moraceae) in northern Australia. *Nematology* **15**, 347-362 (2013).

21 Conchou, L., Cabioch, L., Rodriguez, L. J. & Kjellberg, F. Daily rhythm of mutualistic pollinator activity and scent emission in Ficus septica: ecological differentiation between co-occurring pollinators and potential consequences for chemical communication and facilitation of host speciation. *PLoS One* **9**, e103581, doi:10.1371/journal.pone.0103581 (2014).

22 Zavodna, M. *et al.* Pollinating fig waSPS: genetic consequences of island recolonization. *Journal of Evolutionary Biology* **18**, 1234-1243, doi:10.1111/j.1420-9101.2005.00937.x (2005).

23 Hunt, D. J. *Aphelenchida, Longidoridae and Trichodoridae: Their systematics and bionomics*. (CAB International, 1993).

24 Poinar, G. Parasitodiplogaster-Sycophilon Gen-N, Sp-N (Diplogasteridae, Nematoda), A Parasite of Elisabethiella-Stuckenbergi Grandi (Agaonidae, Hymenoptera) in Rhodesia. *Proceedings of the Koninklijke Nederlandse Akademie Van Wetenschappen Series C-Biological and Medical Sciences* **82**, 375-381 (1979).

25 Dolgin, E. S., Charlesworth, B., Baird, S. E. & Cutter, A. D. Inbreeding and outbreeding depression in Caenorhabditis nematodes. *Evolution; international journal of organic evolution* **61**, 1339-1352, doi:10.1111/j.1558-5646.2007.00118.x (2007).

26 Barriere, A. *et al.* Detecting heterozygosity in shotgun genome assemblies: Lessons from obligately outcrossing nematodes. *Genome Res.* **19**, 470-480, doi:10.1101/gr.081851.108 (2009).

27 Ludewig, A. H. & Schroeder, F. C. Ascaroside signaling in C. elegans. *WormBook*, 1-22, doi:10.1895/wormbook.1.155.1 (2013).

28 Choe, A. *et al.* Ascaroside signaling is widely conserved among nematodes. *Curr. Biol.* **22**, 772-780, doi:10.1016/j.cub.2012.03.024 (2012).

29 Hino, A. *et al.* Karyotype and reproduction mode of the rodent parasite Strongyloides venezuelensis. *Parasitology* **141**, 1736-1745, doi:10.1017/s0031182014001036 (2014).

30 Southey, J. F. *Laboratory methods for work with plant and soil nematodes*. (HMSO Books, 1986).

31 Kajitani, R. *et al.* Efficient de novo assembly of highly heterozygous genomes from whole-genome shotgun short reads. *Genome Res.* **24**, 1384-1395, doi:10.1101/gr.170720.113 (2014).

32 Chin, C.-S. *et al.* Phased diploid genome assembly with single-molecule real-time sequencing. *Nat Meth* **13**, 1050-1054, doi:10.1038/nmeth.4035 http://www.nature.com/nmeth/journal/v13/n12/abs/nmeth.4035.html#supplementary-information (2016).

33 Koren, S., Walenz, B. P., Berlin, K., Miller, J. R. & Phillippy, A. M. Canu: scalable and accurate long-read assembly via adaptive k-mer weighting and repeat separation. *bioRxiv*, doi:10.1101/071282 (2016).

34 Chin, C. S. *et al.* Nonhybrid, finished microbial genome assemblies from long-read SMRT sequencing data. *Nat Methods* **10**, 563-569, doi:10.1038/nmeth.2474 (2013).

35 Wences, A. H. & Schatz, M. C. Metassembler: merging and optimizing de novo genome assemblies. *Genome Biol* **16**, 207, doi:10.1186/s13059-015-0764-4 (2015).

36 Huang, S. *et al.* HaploMerger: reconstructing allelic relationships for polymorphic diploid genome assemblies. *Genome Res.* **22**, 1581-1588, doi:10.1101/gr.133652.111 (2012).

37 Boetzer, M. & Pirovano, W. SSPACE-LongRead: scaffolding bacterial draft genomes using long read sequence information. *BMC Bioinformatics* **15**, 211, doi:10.1186/1471-2105-15-211 (2014).

38 Bonfield, J. K. & Whitwham, A. Gap5--editing the billion fragment sequence assembly. *Bioinformatics* **26**, 1699-1703, doi:10.1093/bioinformatics/btq268 (2010).

39 Otto, T. D., Sanders, M., Berriman, M. & Newbold, C. Iterative Correction of Reference Nucleotides (iCORN) using second generation sequencing technology. *Bioinformatics* **26**, 1704-1707, doi:10.1093/bioinformatics/btq269 (2010).

40 Parra, G., Bradnam, K., Ning, Z., Keane, T. & Korf, I. Assessing the gene space in draft genomes. *Nucleic Acids Res.* **37**, 289-297 (2009).

41 Simão, F. A., Waterhouse, R. M., Ioannidis, P., Kriventseva, E. V. & Zdobnov, E. M. BUSCO: assessing genome assembly and annotation completeness with single-copy orthologs. *Bioinformatics* **31**, 3210-3212, doi:10.1093/bioinformatics/btv351 (2015).

42 Steinbiss, S., Willhoeft, U., Gremme, G. & Kurtz, S. Fine-grained annotation and classification of de novo predicted LTR retrotransposons. *Nucleic Acids Res* **37**, 7002-7013, doi:10.1093/nar/gkp759 (2009).

43 Ellinghaus, D., Kurtz, S. & Willhoeft, U. LTRharvest, an efficient and flexible software for de novo detection of LTR retrotransposons. *BMC Bioinformatics* **9**, 18, doi:10.1186/1471-2105-9-18 (2008).

44 Lee, H. *et al.* MGEScan: a Galaxy-based system for identifying retrotransposons in genomes. *Bioinformatics* **32**, 2502-2504, doi:10.1093/bioinformatics/btw157 (2016).

45 Finn, R. D. *et al.* Pfam: the protein families database. *Nucleic Acids Res* **42**, D222-230, doi:10.1093/nar/gkt1223 (2014).

46 Llorens, C. *et al.* The Gypsy Database (GyDB) of mobile genetic elements: release 2.0. *Nucleic Acids Res.* **39**, D70-D74 (2010).

47 Bessereau, J. L. Transposons in C. elegans. *WormBook*, 1-13, doi:10.1895/wormbook.1.70.1 (2006).

48 Stanke, M., Steinkamp, R., Waack, S. & Morgenstern, B. AUGUSTUS: a web server for gene finding in eukaryotes. *Nucleic Acids Res* **32**, W309-312, doi:10.1093/nar/gkh379 (2004).

49 Carver, T., Harris, S. R., Berriman, M., Parkhill, J. & McQuillan, J. A. Artemis: an integrated platform for visualization and analysis of high-throughput sequence-based experimental data. *Bioinformatics* **28**, 464-469, doi:btr703 [pii]
10.1093/bioinformatics/btr703 [doi] (2012).

50 Altschul, S. F. *et al.* Gapped BLAST and PSI-BLAST: a new generation of protein database search programs. *Nucleic Acids Res* **25**, 3389-3402 (1997).

51 Kim, D., Langmead, B. & Salzberg, S. L. HISAT: a fast spliced aligner with low memory requirements. *Nat Meth* **12**, 357-360, doi:10.1038/nmeth.3317
http://www.nature.com/nmeth/journal/v12/n4/abs/nmeth.3317.html#supplementary-information
(2015).

52 Trapnell, C. *et al.* Differential gene and transcript expression analysis of RNA-seq experiments with TopHat and Cufflinks. *Nat. Protocols* **7**, 562-578 (2012).

53 Conesa, A. *et al.* Blast2GO: a universal tool for annotation, visualization and analysis in functional genomics research. *Bioinformatics* **21**, 3674-3676 (2005).

54 Quevillon, E. *et al.* InterProScan: protein domains identifier. *Nucleic Acids Res.* **33**, W116-120 (2005).

55 Zhao, Z., Liu, H., Wang, C. & Xu, J.-R. Comparative analysis of fungal genomes reveals different plant cell wall degrading capacity in fungi. *BMC Genomics* **14**, 274, doi:10.1186/1471-2164-14-274 (2013).

56 Lombard, V., Golaconda Ramulu, H., Drula, E., Coutinho, P. M. & Henrissat, B. The carbohydrate-active enzymes database (CAZy) in 2013. *Nucleic Acids Res* **42**, D490-495, doi:10.1093/nar/gkt1178 (2014).

57 Emms, D. M. & Kelly, S. OrthoFinder: solving fundamental biases in whole genome comparisons dramatically improves orthogroup inference accuracy. *Genome Biol* **16**, 157, doi:10.1186/s13059-015-0721-2 (2015).

58 Katoh, K., Misawa, K., Kuma, K. & Miyata, T. MAFFT: a novel method for rapid multiple sequence alignment based on fast Fourier transform. *Nucleic Acids Res* **30**, 3059-3066 (2002).

59 Talavera, G. & Castresana, J. Improvement of phylogenies after removing divergent and ambiguously aligned blocks from protein sequence alignments. *Syst Biol* **56**, 564-577, doi:780704285 [pii]
10.1080/10635150701472164 [doi] (2007).

60 Stamatakis, A. RAxML-VI-HPC: maximum likelihood-based phylogenetic analyses with thousands of taxa and mixed models. *Bioinformatics* **22**, 2688-2690 (2006).

61 Cutter, A. D. Divergence times in Caenorhabditis and Drosophila inferred from direct estimates of the neutral mutation rate. *Mol. Biol. Evol.* **25**, 778-786, doi:10.1093/molbev/msn024 (2008).

62 Thomas, C. G. *et al.* Full-genome evolutionary histories of selfing, splitting, and selection in Caenorhabditis. *Genome Res.* **25**, 667-678, doi:10.1101/gr.187237.114 (2015).

63 Yang, Z. PAML 4: phylogenetic analysis by maximum likelihood. *Mol. Biol. Evol.* **24**, 1586-1591, doi:10.1093/molbev/msm088 (2007).

64 Denver, D. R., Morris, K., Lynch, M. & Thomas, W. K. High mutation rate and predominance of insertions in the Caenorhabditis elegans nuclear genome. *Nature* **430**, 679-682, doi:10.1038/nature02697 (2004).

65 Sun, X., Yang, Q. & Xia, X. An improved implementation of effective number of codons (nc). *Mol. Biol. Evol.* **30**, 191-196, doi:10.1093/molbev/mss201 (2013).

66 Han, M. V., Thomas, G. W., Lugo-Martinez, J. & Hahn, M. W. Estimating gene gain and loss rates in the presence of error in genome assembly and annotation using CAFE 3. *Mol. Biol. Evol.* **30**, 1987-1997, doi:10.1093/molbev/mst100 (2013).

67 Gent, J. I. *et al.* Distinct phases of siRNA synthesis in an endogenous RNAi pathway in C. elegans soma. *Mol. Cell* **37**, 679-689, doi:10.1016/j.molcel.2010.01.012 (2010).

68 Fischer, S. E. J. *et al.* The ERI-6/7 Helicase Acts at the First Stage of an siRNA Amplification Pathway That Targets Recent Gene Duplications. *PLOS Genetics* **7**, e1002369, doi:10.1371/journal.pgen.1002369 (2011).

69 Vasale, J. J. *et al.* Sequential rounds of RNA-dependent RNA transcription drive endogenous small-RNA biogenesis in the ERGO-1/Argonaute pathway. *Proceedings of the National Academy of Sciences* **107**, 3582-3587, doi:10.1073/pnas.0911908107 (2010).

70 Das, P. P. *et al.* Piwi and piRNAs act upstream of an endogenous siRNA pathway to suppress Tc3 transposon mobility in the Caenorhabditis elegans germline. *Mol. Cell* **31**, 79-90, doi:10.1016/j.molcel.2008.06.003 (2008).

71 Zhang, C. *et al.* mut-16 and other mutator class genes modulate 22G and 26G siRNA pathways in Caenorhabditis elegans. *Proc. Natl. Acad. Sci. U. S. A.* **108**, 1201-1208, doi:10.1073/pnas.1018695108 (2011).

72 Robertson, H. M. & Thomas, J. H. The putative chemoreceptor families of C. elegans. *WormBook*, 1-12, doi:10.1895/wormbook.1.66.1 (2006).

73 Thomas, J. H. & Robertson, H. M. The Caenorhabditis chemoreceptor gene families. *BMC biology* **6**, 42 (2008).

74 Price, M. N., Dehal, P. S. & Arkin, A. P. FastTree 2--approximately maximum-likelihood trees for large alignments. *PLoS ONE* **5**, e9490, doi:10.1371/journal.pone.0009490 [doi] (2010).

75 Haas, B. J., Delcher, A. L., Wortman, J. R. & Salzberg, S. L. DAGchainer: a tool for mining segmental genome duplications and synteny. *Bioinformatics* **20**, 3643-3646, doi:10.1093/bioinformatics/bth397 (2004).

76 Krzywinski, M. *et al.* Circos: an information aesthetic for comparative genomics. *Genome Res.* **19**, 1639-1645, doi:10.1101/gr.092759.109 (2009).

77 Stein, L. D. *et al.* The genome sequence of Caenorhabditis briggsae: a platform for comparative genomics. *PLoS Biol.* **1**, E45 (2003).

78 Cutter, A. D., Dey, A. & Murray, R. L. Evolution of the Caenorhabditis elegans genome. *Mol. Biol. Evol.* **26**, 1199-1234, doi:10.1093/molbev/msp048 (2009).

79 Ross, J. A. *et al.* Caenorhabditis briggsae recombinant inbred line genotypes reveal inter-strain incompatibility and the evolution of recombination. *PLoS Genet* **7**, e1002174, doi:10.1371/journal.pgen.1002174 (2011).

80 Hung, S. S., Wasmuth, J., Sanford, C. & Parkinson, J. DETECT—a Density Estimation Tool for Enzyme ClassificaTion and its application to Plasmodium falciparum. *Bioinformatics* **26**, 1690-1698, doi:10.1093/bioinformatics/btq266 (2010).

81 Claudel-Renard, C., Chevalet, C., Faraut, T. & Kahn, D. Enzyme-specific profiles for genome annotation: PRIAM. *Nucleic Acids Res.* **31**, 6633-6639, doi:10.1093/nar/gkg847 (2003).

82 Moriya, Y., Itoh, M., Okuda, S., Yoshizawa, A. C. & Kanehisa, M. KAAS: an automatic genome annotation and pathway reconstruction server. *Nucleic Acids Res* **35**, W182-185, doi:10.1093/nar/gkm321 (2007).

83 Yilmaz, L. S. & Walhout, A. J. A Caenorhabditis elegans Genome-Scale Metabolic Network Model. *Cell systems* **2**, 297-311, doi:10.1016/j.cels.2016.04.012 (2016).

84 Karp, P. D. *et al.* Pathway Tools version 13.0: integrated software for pathway/genome informatics and systems biology. *Brief Bioinform* **11**, 40-79, doi:10.1093/bib/bbp043 (2010).

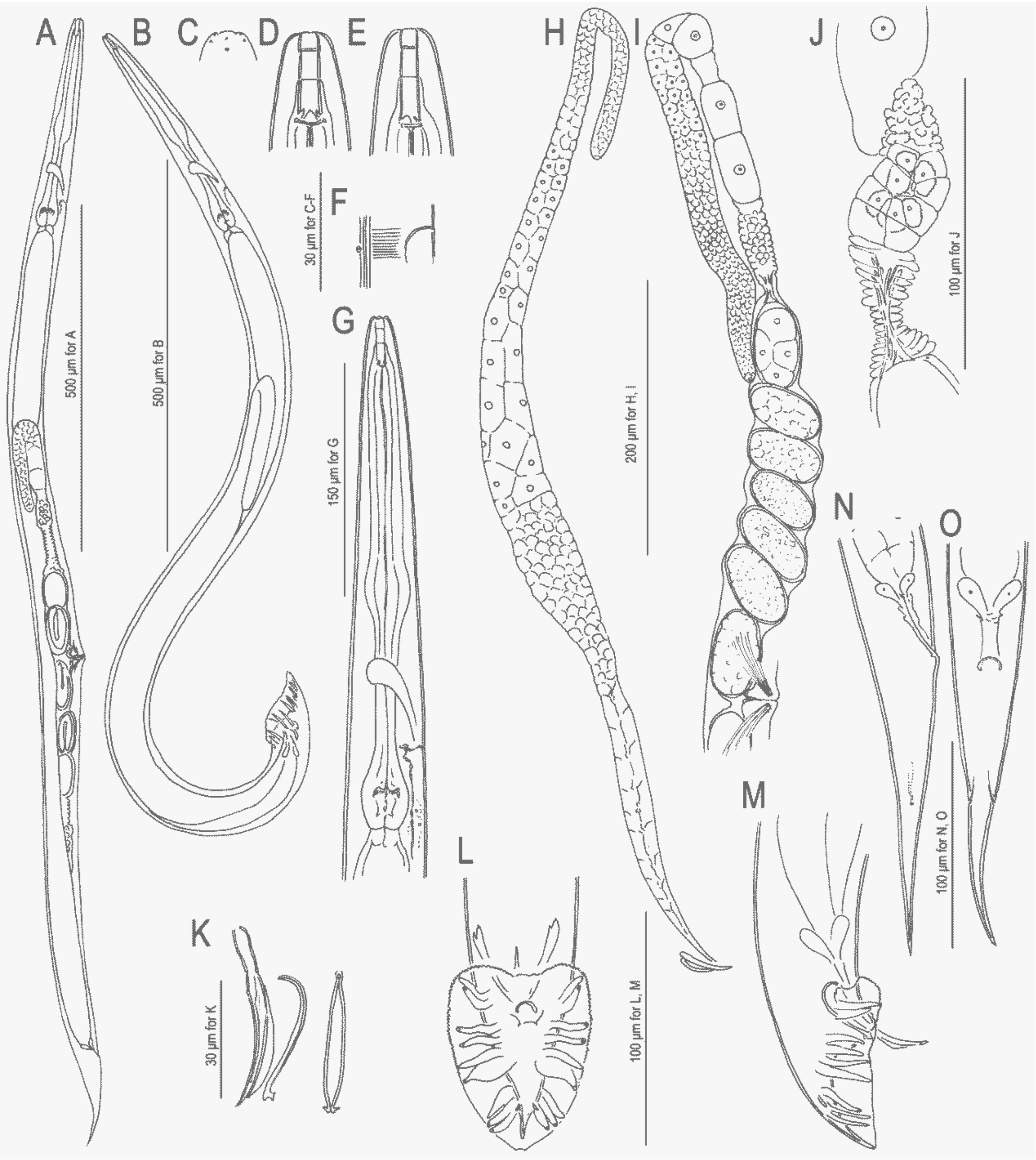

Supplementary Fig. 1. *Caenorhabditis inopinata* n. sp. adult female and male.

A: Female; B: Male; C: Lip surface of male; D, E: Stomatal region in ventral view (D) and left lateral view (E); F: Body surface structure including deirid, lateral field, annulation and relative position of excretory pore; G: Neck region; H: Whole male gonad in right lateral view; I: Anterior female gonad in right lateral view; L: Close-up of uterus-spermatheca junction of anterior gonad in right lateral view; K: Spicule and gubernaculum in left lateral view and ventral view of gubernaculum; L, M: Male tail in ventral view (L) and right lateral view (M); N, O: Female tail in right lateral view (N) and ventral view (O).

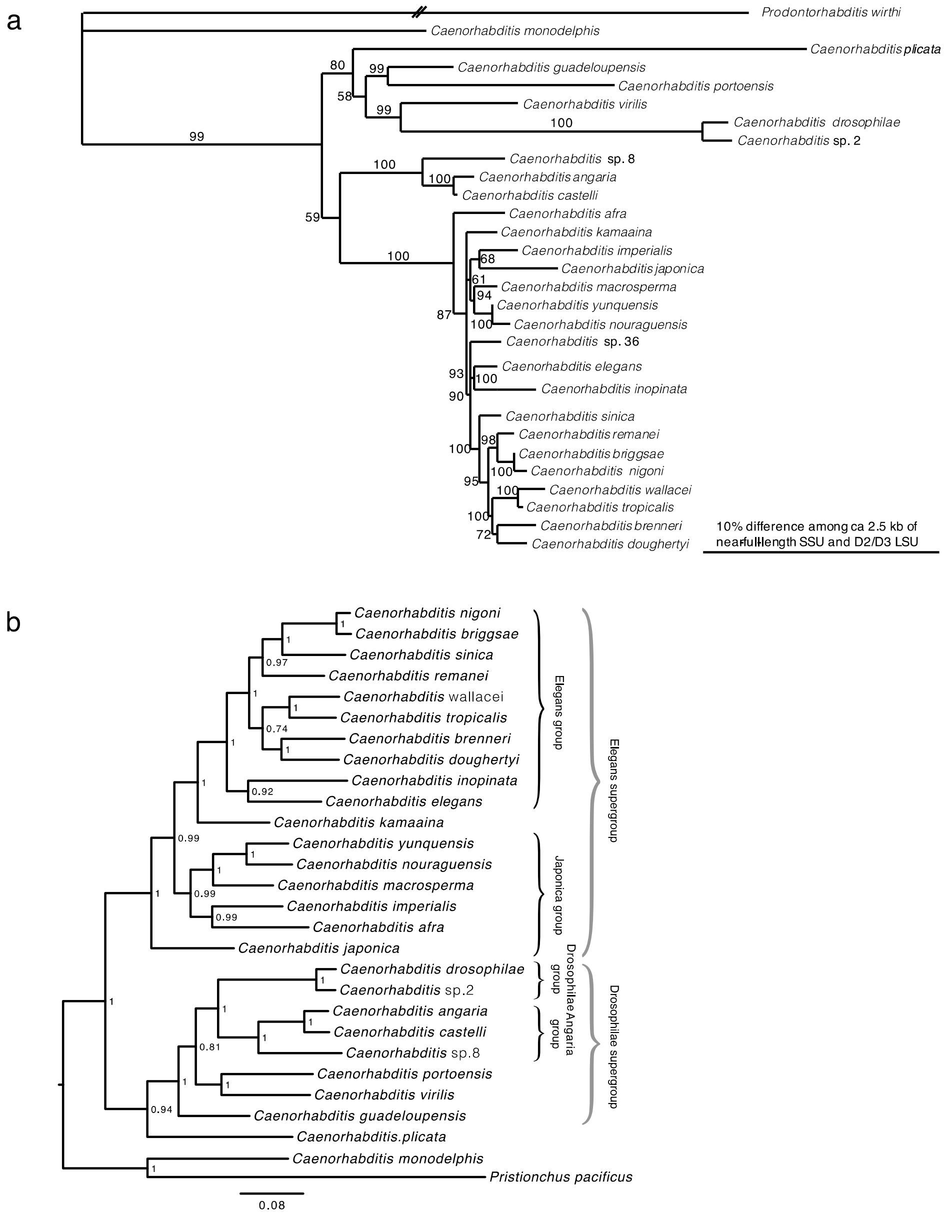

Supplementary Fig. 2. Phylogenetic relationships of *C. inopinata* and the other 26 *Caenorhabditis* species inferred by **a)** near-full-length 18S and D2/D3 region of 28S ribosomal RNA genes with *Prodontorhabditis wirthi* as an outgroup and **b)** 11 conserved genes (genes for SSU and LSU rRNA, orthologues of *C. elegans ama-1*, *lin-44*, *par-6*, *pkc-3*, ZK686.3, W02B12.9, ZK795.3, Y97E10AL.2 and Y45G12B.2a) [3] with *Pristionchus pacificus* as an out group. Nucleotide (a) or amino acid sequences (b) were aligned before concatenation, and maximum likelihood trees were generated with RAxML v7.2.8 using the best-fitting empirical model with 1,000 bootstrap resampling replicates with the percentage support shown on the nodes. The scale bar shows nucleotide (a) or amino acid (b) substitutions per site.

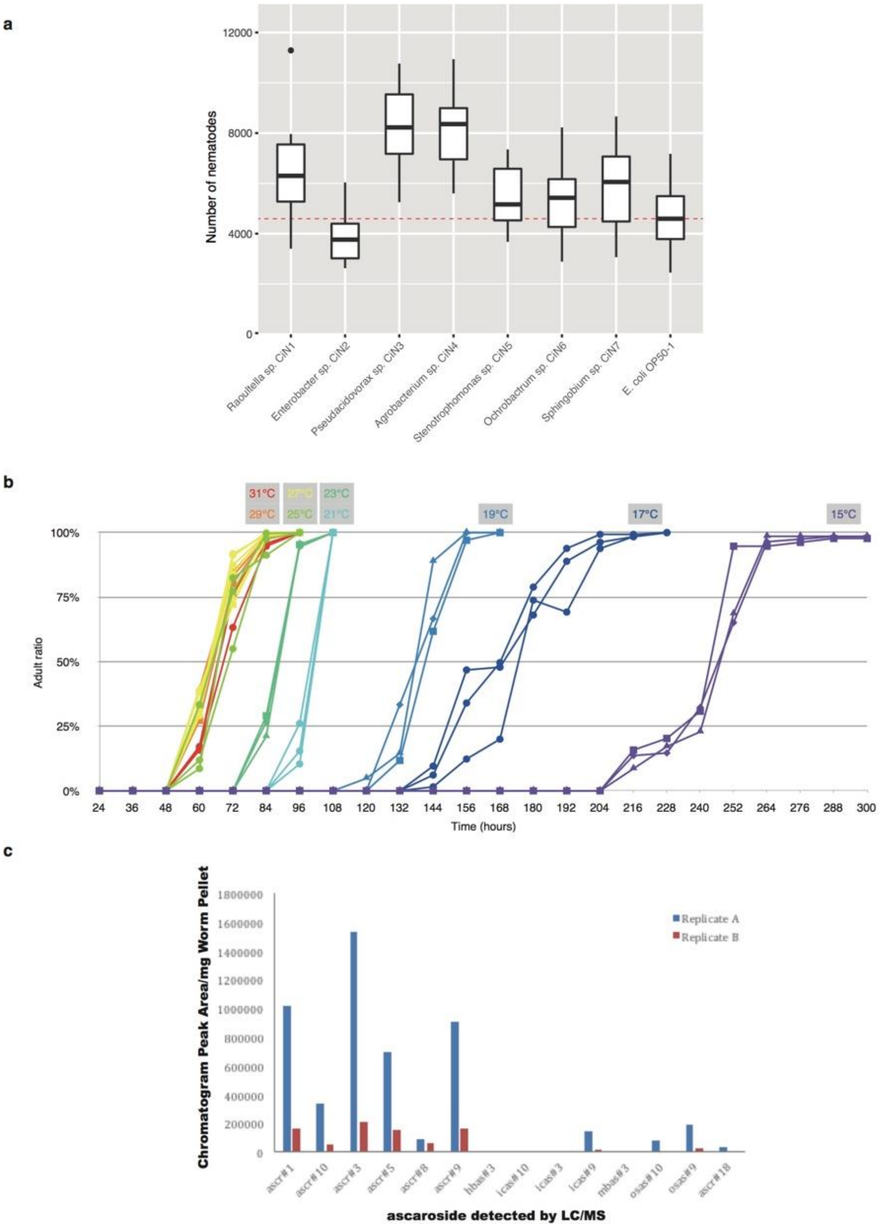

Supplementary Fig. 3. Culture condition of *C. inopinata.* **a)** Nematode population size on individual bacterial isolates. Population size was measured as total offspring of 100 females and 100 males after incubation at 25ºC for 15 days. Results are shown for an *E. coli* control and seven bacterial isolates: *Raoultella* sp. CiN1, *Enterobacter* sp. CiN2, *Pseudacidovorax* sp. CiN3, *Agrobacterium* sp. CiN4, *Stenotrophomonas* sp. CiN5, *Ochrobactrum* sp. CiN6 and *Sphingobium* sp. CiN7. Each box represents the interquartile range of values (the 25th and 75th centiles), with split at the median. Whiskers extend to the furthest points that are within 1.5 times the length of the box. The red broken line represents *E. coli* median. **b)** Effect of cultivation temperature on *C. inopinata* growth. Eggs collected from the adult females were synchronised in M9 buffer, then approximately 200 synchronised L2 were transferred to each NGM plate seeded with *E. coli* OP50-1 and cultivated at different temperatures. Nematode development was observed every 12 hr after transfer to NGM plates. A next generation was

not observed at 31ºC or higher. **c)** Relative amount of ascarosides detected in mixed cultures of *C. inopinata.* Relative peak intensities of major ascarosides for two replicates are shown.

a

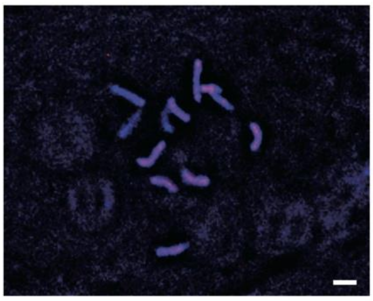

b

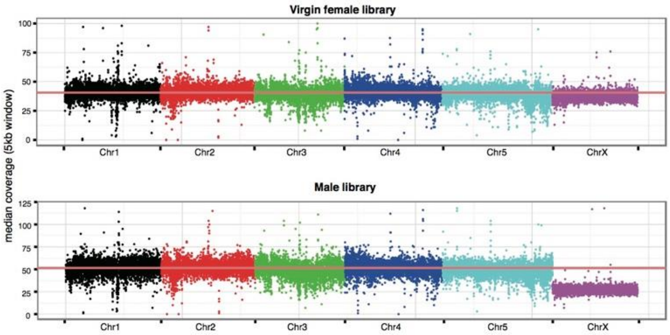

c

Lineage	Genes	Median dS	Median dS' (First-Ninth Decile)	T (Generation * 10^6)	T_{14} days (Year * 10^6)	T_{30} days (Year * 10^6)	T_{60} days (Year * 10^6)
C. angaria	391	1.225	1.667 (0.707-3.384)	185.22	7.10	15.22	30.45
C. jaopnica	724	0.964	1.602 (0.959-2.809)	177.98	6.83	14.63	29.26
C. elegans	892	0.668	1.150 (0.785-1.837)	127.76	4.90	10.50	21.00
C. inopinata	903	0.839	1.285 (0.877-2.094)	142.73	5.47	11.73	23.46
C. tropicalis	637	0.658	1.278 (0.873-2.122)	141.94	5.44	11.67	23.33
C. brenneri	839	0.578	1.156 (0.791-1.953)	128.40	4.92	10.55	21.11
C. remanei	906	0.547	1.013 (0.695-1.568)	112.51	4.32	9.25	18.49
C. sinica	634	0.521	1.093 (0.778-1.855)	121.42	4.66	9.98	19.96
C. briggsae	827	0.543	0.970 (0.663-1.543)	107.72	4.13	8.85	17.71

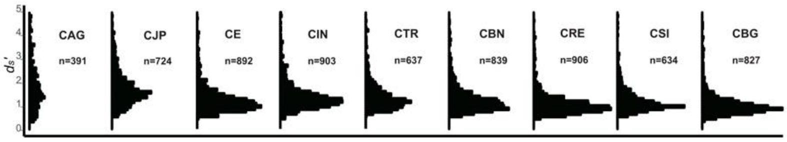

Supplementary Fig. 4. Karyotypes and divergence estimate. **a)** DAPI stained chromosome spreads in *C. inopinata* mitotic metaphases. *C. inopinata* chromosome number was observed to be 2n=12. Scale bar=2 µm. **b)** Whole-genome read coverage depth for female and male along the chromosomes. Median coverage by 5 kb window was shown by dots and the median coverage of all chromosomes were shown by red lines. **c)** Divergence estimates of *Caenorhabditis* species. The table shows number of orthologues genes used for calculation in each species, synonymous change rate (dS), adjusted dS, estimated separated generation time based on the *C. elegans* mutation rate (μ = 9.0 × 10-9 mutations per generation) and separation times calculated with 14, 30 and 60-day average generation time. The plots show distribution of adjusted synonymous change rate in each species. CAG: *C. angaria*, CJP: *C. japonica*, CE: *C. elegans*, CIN: *C. inopinata*, CTR: *C. tropicalis*, CBN: *C. brenneri*, CRE: *C. remanei*, CSI: *C. sinica*, CBG: *C. briggsae*.

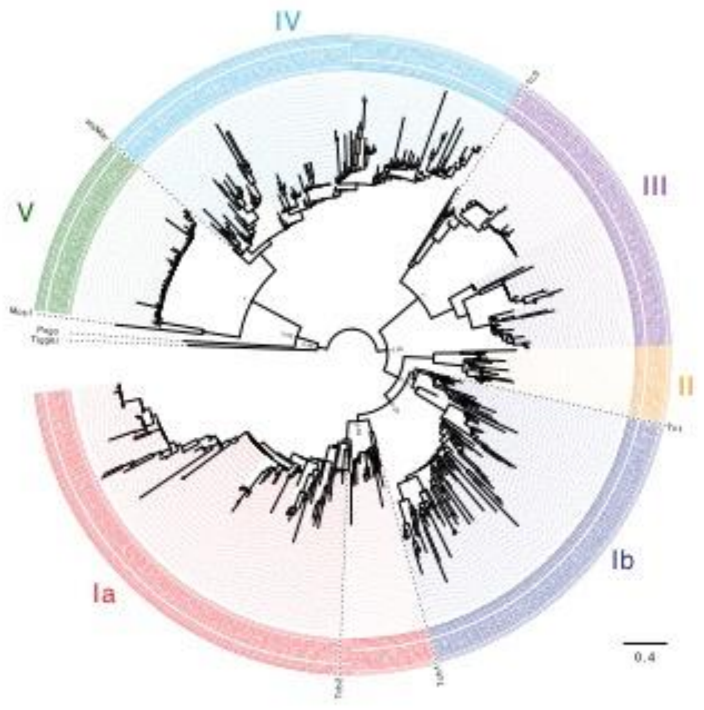

Supplementary Fig. 5. Phylogenetic analysis of Tc1/Mariner-like transposases found in *C. inopinata*. The 702 Tc1-like transposase sequences predicted from the *C. inopinata* genome formed five main clusters. The largest (I) could be further divided in to two subclusters which were most closely related to the *C. briggsae* transposase Tcb1 and Tcb2 (Ia) and *C. elegans* Tc1 (Ib). The smallest cluster (II) comprised ~20 transposase sequences and did not cluster with other species sequences. *C. inopinata* transposase sequences in clusters III, IV, and V were mostly closely related to *C. elegans* Tc3 transposase, Human HsMar and the *Drosophila melanogaster* Mos1, respectively. The scale bar represents amino acid substitutions per site.

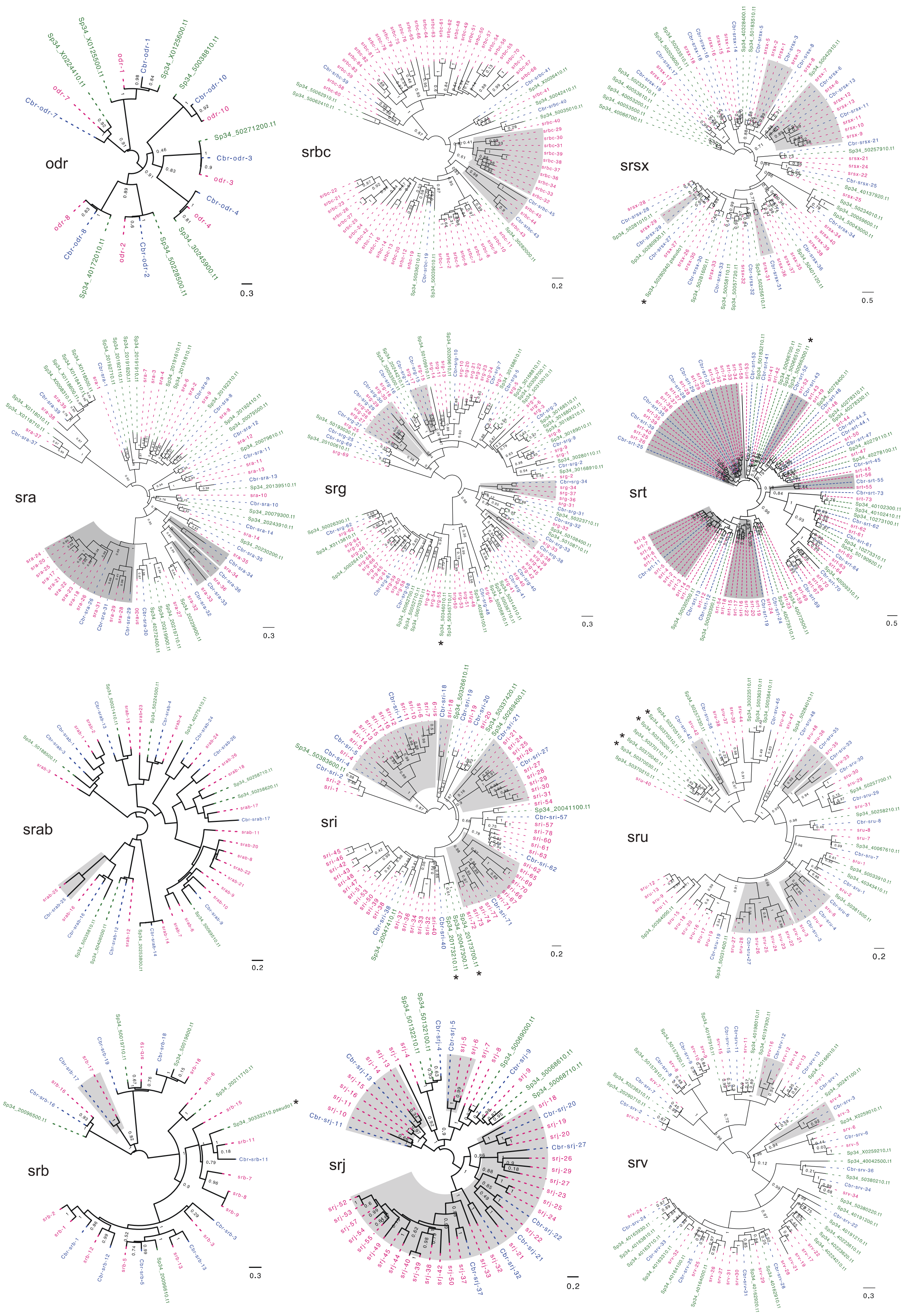
odr
srbc
srsx
sra
srg
srt
srab
sri
sru
srb
srj
srv

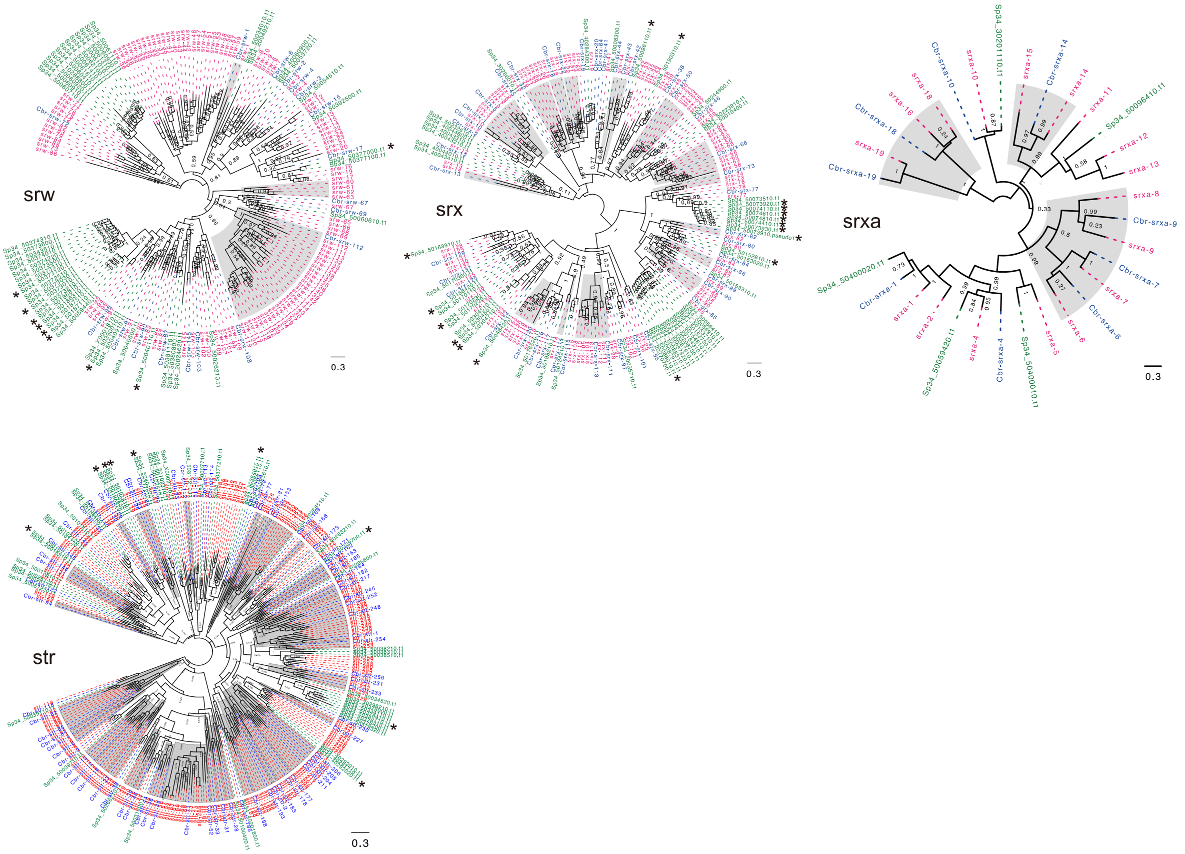

Supplementary Fig. 6. Maximum-likelihood trees of gene families of serpentine receptors in *C. inopinata*, *C. elegans* and *C. briggsae*. Amino acid sequences of each serpentine family from the three species were aligned using Mafft v7.221 [58] and maximum-likelihood trees were constructed using RAxML v7.2.8 [60] under the best-fitting empirical model of amino acid substitution with 1,000 bootstrap resampling. Scale bars show amino acid substitutions per site. Trees for *srd*, *srh* and *sre* are shown in Figure 4b. Green, pink and blue represent genes in *C. inopinata*, *C. elegans* and *C. briggsae,* respectively.

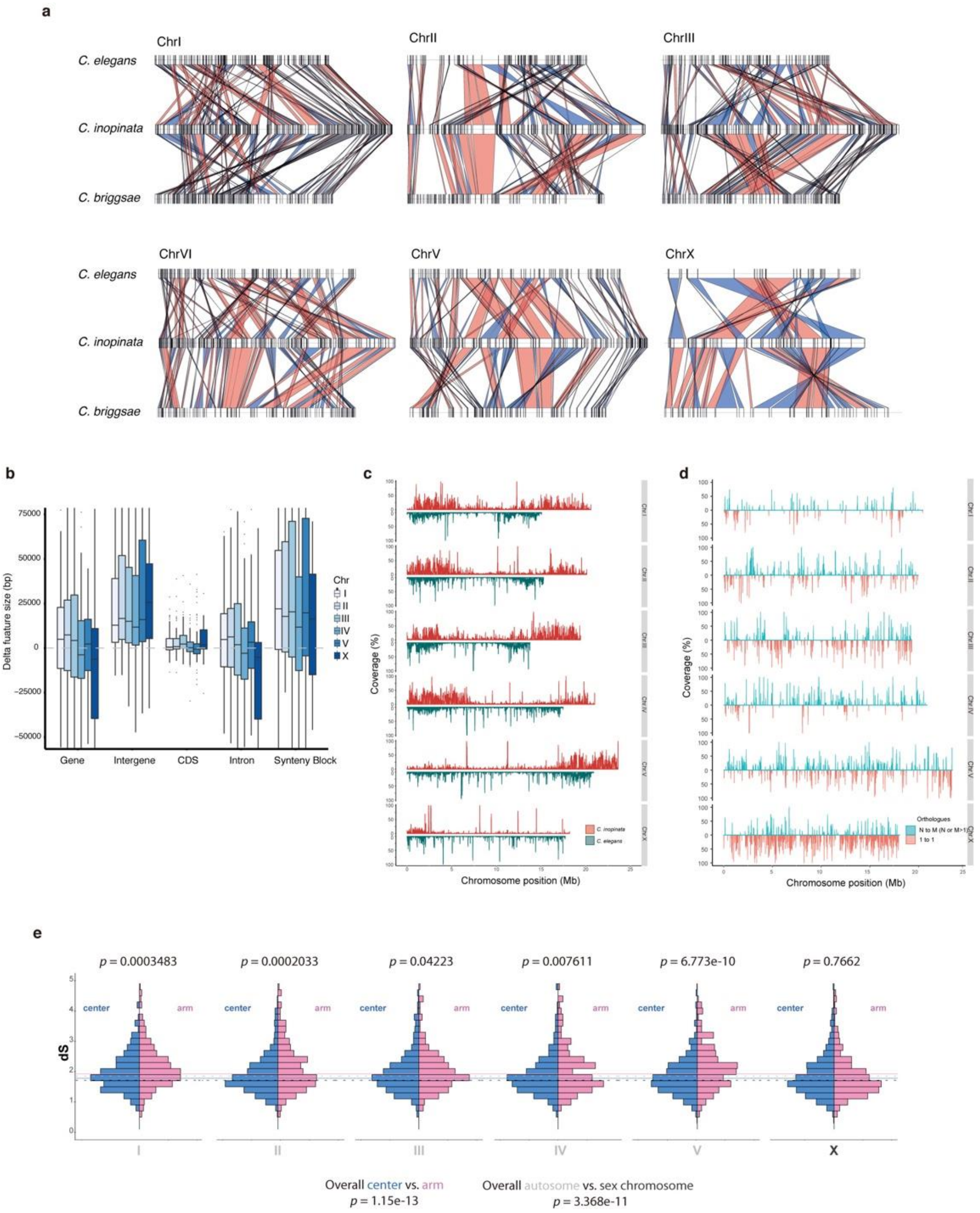

Supplementary Fig. 7. Structural evolution in *C. inopinata* genome. **a)** Overall linking of synteny blocks among *C. elegans*, *C. inopinata* and *C. briggsae*. Horizontal lines show chromosomes of the three species. Vertical black lines on chromosome represent start positions of the first gene in synteny blocks or end positions of the last gene in synteny blocks. **b)** Difference in genomic feature size per synteny block between *C. inopinata* and *C. elegans*. For each synteny block, delta is expressed as the total extent (in bp) occupied by each type of feature in *C. inopinata* minus the total extent of the same feature type in its syntenic relative *C. elegans*. **c)** Distribution of tandem repeats in *C. inopinata* and *C. elegans*. Panels are separated by chromosomes. Y axis is the coverage of repeat content in 10-kb sliding windows, and X axis is the position on chromosome in Mb. Distribution of *C. inopinata* tandem repeats is in red and *C. elegans* in green. **d)** Distribution of one to one orthologues and many to many orthologous families in *C. inopinata*. Panels are separated by chromosomes. Y axis

is the coverage of orthologues content in 10-kb sliding windows, and X axis is the position on chromosome in Mb. one-to-one orthologues are in red and N-to-M (N or M>1) are in green. **e**) Comparison of synonymous change rate in *C. inopinata* vs. *C. elegans*. Distribution of dS within centre regions are denoted in blue, and that of arm regions are in red. P-values of two-sample Wilcoxon test of synonymous rate between centre and arm in each chromosome are labelled on top of each chromosome panel. P-values of two-sample Wilcoxon test of synonymous rate between overall chromosomal centre and arm as well as autosomes (in black) and sex chromosomes (X, in grey) are labelled at bottom.

Supplementary Table 2. Mating incompatibility between *C. inopinata* and *C. elegans* as well as with five additional *Caenorhabditis* species.

	Cross[a]	Result[b,c]	N[d]	Notes
***C. elegans* crosses**	*C. inopinata* NKZ35 conspecific (reference strain)	Viable F1 and F2 progeny	many (>>10)	various including 1 ♀ X 1 ♂
	C. inopinata ♀ X *C. elegans* N2 ♂	Mating behavior observed (N=2), no embryos	5	2 ♀ X 2 ♂
	C. inopinata ♀ X *C. elegans* fog-2 (q71) ♂	Mating behavior observed; no embryos; no sperm transfer observed (n=16)	13	3 ♀ X 5 ♂
	C. elegans fog-2 (q71) pseudo ♀ X *C. inopinata* ♂	Mating behavior observed; no embryos; no sperm transfer observed (n=15)	21	3 pseudo♀ X 5 ♂
	C. elegans N2 ⚥ X *C. inopinata* ♂	No sterilization of hermaphrodites observed	3	3 ⚥ X 5 ♂
	C. elegans dpy-5(e61) ⚥ X *C. inopinata* ♂	No mating behavior observed; no sterilization	5	3 ⚥ X 5 ♂
	C. elegans unc-119(ed9); ttTi5605 BRC189 ⚥ X *C. inopinata* ♂	No mating behavior observed; no sterilization	5	2 ⚥ X 2 ♂
	C. elegans fog-2 (q71) conspecific	Viable F1 and F2 progeny	3	3 pseudo♀ X 5 ♂ initial P0 cross
**Other *Caenorhabditis* crosses**	*C. inopinata* ♀ X *C. briggsae* AF16 ♂	Mating behavior observed (N=1); no embryos	3	2 ♀ X 2 ♂
	C. briggsae dpy(ant4) BRC252 ⚥ X *C. inopinata* ♂	No mating behavior observed; no sterilization	3	2 ♀ X 2 ♂
	C. inopinata ♀ X *C. brenneri* JU1397 ♂	Mating behavior observed (N=1); no embryos	3	2 ♀ X 2 ♂
	C. brenneri JU1397 ♀ X *C. inopinata* ♂	Mating plug (N=2); embryos observed but no larvae (N=1)	3	2 ♀ X 2 ♂
	C. inopinata ♀ X *C. remanei* BRC20108 ♂	Mating behavior observed (N=2); mating plug; no embryos	3	2 ♀ X 2 ♂
	C. remanei BRC20108 ♀ X *C. inopinata* ♂	Mating behavior observed (N=1); mating plug (N=2); no embryos	3	2 ♀ X 2 ♂
	C. inopinata ♀ X *C. guadeloupensis* NIC113 ♂	Mating behavior observed (N=1); mating plug (N=1); no embryos	3	2 ♀ X 2 ♂
	C. guadeloupensis NIC113 ♀ X *C. inopinata* ♂	Males and females touching but no obvious mating behavior (N=2); no embryos	3	2 ♀ X 2 ♂
	C. inopinata ♀ X *C*. sp. 50 BRC20387 ♂	No mating behavior observed; no embryos	3	2 ♀ X 2 ♂
	C. sp. 50 BRC20387 ♀ X *C. inopinata* ♂	No mating behavior observed; no embryos	3	2 ♀ X 2 ♂

a All C. inopinata were strain NKZ35

b Observations were limited to 2-5 min periods 2-3 times per day

c Observations occurred on all independent crosses (plates), unless indicated by N (number of plates)

d N, number of independent crosses (plates); n, number of individual hermaphrodites examined

Supplementary Table 3. Germline development in C. inopinata adult females cultured with E. coli strain OP50-1 and strain HT115.

Gonad morphology	OP50-1 (N=27)				HT115(N=30)			
	Ratio	Wide arm	Yolk in body cavity	Average number of oocytes	Ratio	Wide arm	Yolk in body cavity	Average number of oocytes
U-shape (normal)	11/27 (40.7%)	2/11	3/11	3.7	14/30 (46.7%)	6/14	0/6	4.3
Irregular U-shape	2/27 (7.4%)	1/2	2/2	5.5	11/30 (36.7%)	5/11	1/11	4.6
Irregular shape	13/27 (48.1%)	3/13	8/13	2.5	5/30 (16.7%)	2/5	1/5	3.8
Gonad-less	1/27 (3.7%)	-	1/1	-	0/30 (0%)	-	-	-

Wide arm: widths of gonadal arms are nearly half of the body width. Yolk in body cavity: yolk secreted from intestine remains in the body cavity, an indication of underdeveloped gonads.

Supplementary Table 4. Bacterial strains isolated from *C. inopinata*

	Isolation information				Taxonomic assignment by SINA (RDP/GREENGENES/SILVA)			BLASTn search information				
Isolates	Sample type	Location	Date	Taxonomic group**	Taxonomy (Family/Genus)	Identity (%)	Score	Identified	Identity (%)	E-value	Bit score (Max)	Accession
CiN001*	Nematodes (*C.inopinata*)	Ishigaki Island, JP	20/5/2016	1	*Enterobacteriaceae/unclassified*	98.18	99	*Raoultella ornithinolytica* strain ATCC 31898	98.18	0	2396	NR_114502.1
CiN002*	Nematodes (*C.inopinata*)	Ishigaki Island, JP	20/5/2016	1	*Enterobacteriaceae/Enterobacter*	99.78	99	*Enterobacter xiangfangensis* strain 10-17	99.85	0	2475	NR_126208.1
CiN003*	Nematodes (*C.inopinata*)	Ishigaki Island, JP	20/5/2016	3	*Comamonadaceae/Pseudacidovorax*	96.73	99	*Pseudacidovorax intermedius* strain CC-21	96.79	0	2231	NR_044241.1
CiN004*	Nematodes (*C.inopinata*)	Ishigaki Island, JP	20/5/2016	4	*Rhizobiaceae/Agrobacterium*	99.92	99	*Agrobacterium tumefaciens* strain IAM 12048	99.92	0	2246	NR_116306.1
CiN005*	Nematodes (*C.inopinata*)	Ishigaki Island, JP	20/5/2016	2	*Xanthomonadaceae/Stenotrophomonas*	97.52	98	*Stenotrophomonas panacihumi* strain MK06	97.54	0	2357	NR_117406.1
CiN006*	Nematodes (*C.inopinata*)	Ishigaki Island, JP	20/5/2016	4	*Brucellaceae/Ochrobactrum*	97.47	97	*Ochrobactrum pseudintermedium* strain ADV31	97.34	0	2233	NR_043756.1
CiN007*	Nematodes (*C.inopinata*)	Ishigaki Island, JP	20/5/2016	5	*Sphingomonadaceae/Sphingobium*	97.62	99	*Sphingobium rhizovicinum* strain CC-FH12-1	99.01	0	2351	NR_044226.1
CiN008	Nematodes (*C.inopinata*)	Ishigaki Island, JP	20/5/2016	2	*Xanthomonadaceae/Stenotrophomonas*	99.48	99	*Stenotrophomonas pavanii strain LMG 25348*	98.96	0	1031	NR_118008.1
CiN009	Nematodes (*C.inopinata*)	Ishigaki Island, JP	20/5/2016	7	*Enterobacteriaceae/unclassified*	99.46	99	*Enterobacter aerogenes strain KCTC 2190*	99.19	0	667	NR_102493.1
CiN010	Nematodes (*C.inopinata*)	Ishigaki Island, JP	20/5/2016	1	*Enterobacteriaceae/Enterobacter*	98.85	97	*Enterobacter oryziphilus strain REICA_142*	98.8	0	1037	NR_125587.1
CiN011	Nematodes (*C.inopinata*)	Ishigaki Island, JP	20/5/2016	1	*Enterobacteriaceae/unclassified*	97.76	97	*Raoultella terrigena strain NBRC 14941*	98.31	7.00E-178	621	NR_113703.1
CiN012	Nematodes (*C.inopinata*)	Ishigaki Island, JP	20/5/2016	2	*Xanthomonadaceae/Stenotrophomonas*	99.80	99	*Stenotrophomonas maltophilia strain ATCC 19861*	98.99	0	891	NR_040804.1
CiN013	Nematodes (*C.inopinata*)	Ishigaki Island, JP	20/5/2016	1	*Enterobacteriaceae/Enterobacter*	98.29	98	*Citrobacter murliniae strain CDC 2970-59*	98.7	0	1092	NR_028688.1
CiN014	Nematodes (*C.inopinata*)	Ishigaki Island, JP	20/5/2016	1	*Enterobacteriaceae/unclassified*	99.84	99	*Raoultella terrigena strain ATCC 33257*	99.47	0	2422	NR_114503.1
CiN015	Nematodes (*C.inopinata*)	Ishigaki Island, JP	20/5/2016	1	*Enterobacteriaceae/-*	98.22	97	*Klebsiella oxytoca strain JCM1665*	98.21	0	2340	NR_112010.1
CiN016	Nematodes (*C.inopinata*)	Ishigaki Island, JP	20/5/2016	1	*Enterobacteriaceae/unclassified*	97.40	95	*Citrobacter murliniae strain CDC 2970-59*	100	0	833	NR_028688.1
CiN017	Nematodes (*C.inopinata*)	Ishigaki Island, JP	20/5/2016	1	*Enterobacteriaceae/Enterobacter*	99.82	99	*Enterobacter cancerogenus strain LMG 2693*	98.87	0	1419	NR_116756.1
CiN018	Nematodes (*C.inopinata*)	Ishigaki Island, JP	20/5/2016	1	*Enterobacteriaceae/Enterobacter*	99.12	97	*Klebsiella oxytoca strain ATCC 13182*	99.36	0	1131	NR_041749.1
CiN019	Nematodes (*C.inopinata*)	Ishigaki Island, JP	20/5/2016	1	*Enterobacteriaceae/Enterobacter*	99.46	98	*Enterobacter cancerogenus strain LMG 2693*	99.33	0	1341	NR_116756.1
CiN020	Nematodes (*C.inopinata*)	Ishigaki Island, JP	20/5/2016	1	*Enterobacteriaceae/unclassified*	99.61	99	*Raoultella terrigena strain ATCC 33257*	99.61	0	1397	NR_114503.1
CiN021	Nematodes (*C.inopinata*)	Ishigaki Island, JP	20/5/2016	1	*Enterobacteriaceae/-*	99.08	99	*Klebsiella oxytoca strain JCM1665*	98.7	0	2316	NR_112010.1
CiN022	Nematodes (*C.inopinata*)	Ishigaki Island, JP	20/5/2016	1	*Enterobacteriaceae/unclassified*	99.48	98	*Citrobacter freundii strain NBRC 12681*	98.94	4.00E-93	339	NR_113596.1
CiN023	Nematodes (*C.inopinata*)	Ishigaki Island, JP	20/5/2016	1	*Enterobacteriaceae/unclassified*	98.58	98	*Escherichia vulneris strain NBRC 102420*	98.79	0	880	NR_114080.1
CiN024	Nematodes (*C.inopinata*)	Ishigaki Island, JP	20/5/2016	1	*Enterobacteriaceae/Enterobacter*	99.44	98	*Enterobacter asburiae strain JCM6051*	99.25	0	966	NR_024640.1
CiN025	Nematodes (*C.inopinata*)	Ishigaki Island, JP	20/5/2016	1	*Enterobacteriaceae/Enterobacter*	98.91	98	*Enterobacter asburiae strain JCM6051*	99.18	0	658	NR_024640.1
CiN026	Nematodes (*C.inopinata*)	Ishigaki Island, JP	20/5/2016	1	*Enterobacteriaceae/Enterobacter*	99.43	98	*Enterobacter cancerogenus strain LMG 2693*	99.5	0	1450	NR_116756.1
CiN027	Nematodes (*C.inopinata*)	Ishigaki Island, JP	20/5/2016	1	*Enterobacteriaceae/Enterobacter*	99.87	99	*Enterobacter cancerogenus strain LMG 2693*	99.74	0	1426	NR_116756.1
CiN028	Nematodes (*C.inopinata*)	Ishigaki Island, JP	20/5/2016	7	*Enterobacteriaceae/-*	94.55	89	*Cronobacter zurichensis strain LMG 23730*	94.39	0	965	NR_104924.1
CiN029	Nematodes (*C.inopinata*)	Okinawa, JP	22/5/2016	1	*Enterobacteriaceae/-*	98.35	99	*Raoultella ornithinolytica strain ATCC 31898*	98.35	0	2230	NR_114502.1
CiN030	Nematodes (*C.inopinata*)	Okinawa, JP	22/5/2016	1	*Enterobacteriaceae/Enterobacter*	99.46	98	*Enterobacter ludwigii strain EN-119*	99.22	0	2316	NR_042349.1
CiN031	Nematodes (*C.inopinata*)	Okinawa, JP	22/5/2016	8	*Bacillaceae/Aeribacillus*	100.00	99	*Aeribacillus pallidus strain DSM 3670*	99.8	0	920	NR_026515.1
CiN032	Nematodes (*C.inopinata*)	Okinawa, JP	22/5/2016	1	*Enterobacteriaceae/Enterobacter*	99.84	99	*Enterobacter xiangfangensis strain 10-17*	99.84	0	2335	NR_126208.1
CiN033	Nematodes (*C.inopinata*)	Okinawa, JP	22/5/2016	1	*Enterobacteriaceae/Enterobacter*	98.80	97	*Enterobacter ludwigii strain EN-119*	98.64	0	2200	NR_042349.1
CiN034	Nematodes (*C.inopinata*)	Okinawa, JP	22/5/2016	1	*Enterobacteriaceae/Enterobacter*	99.84	99	*Enterobacter xiangfangensis strain 10-17*	98.71	0	1369	NR_126208.1
CiN035	Nematodes (*C.inopinata*)	Okinawa, JP	22/5/2016	1	*Enterobacteriaceae/Enterobacter*	99.51	98	*Enterobacter cancerogenus strain LMG 2693*	99.38	0	1465	NR_116756.1
CiN036	Nematodes (*C.inopinata*)	Okinawa, JP	22/5/2016	8	*Bacillaceae/Aeribacillus*	98.08	97	*Aeribacillus pallidus strain DSM 3670*	97.5	0	891	NR_026515.1
CiN037	Nematodes (*C.inopinata*)	Okinawa, JP	22/5/2016	1	*Enterobacteriaceae/Pantoea*	98.10	97	*Pantoea cypripedii strain LMG 2657*	97.24	0	2156	NR_118394.1
CiN038	Nematodes (*C.inopinata*)	Okinawa, JP	22/5/2016	1	*Enterobacteriaceae/Enterobacter*	99.52	98	*Enterobacter ludwigii strain EN-119*	99.29	0	2272	NR_042349.1
CiN039	Nematodes (*C.inopinata*)	Okinawa, JP	22/5/2016	1	*Enterobacteriaceae/Enterobacter*	99.28	97	*Enterobacter cancerogenus strain LMG 2693*	99.14	0	1247	NR_116756.1
CiN040	Nematodes (*C.inopinata*)	Okinawa, JP	22/5/2016	4	*Brucellaceae/Ochrobactrum*	98.21	98	*Ochrobactrum haematophilum strain CCUG 38531*	98.37	0	1945	NR_042588.1
CiN041	Nematodes (*C.inopinata*)	Okinawa, JP	22/5/2016	3	*Comamonadaceae/Comamonas*	97.67	98	*Pseudacidovorax intermedius strain CC-21*	97.5	0	2050	NR_044241.1
CiN042	Nematodes (*C.inopinata*)	Okinawa, JP	22/5/2016	3	*Comamonadaceae/Pseudacidovorax*	96.74	98	*Pseudacidovorax intermedius strain CC-21*	96.97	0	2106	NR_044241.1
CiN043	Nematodes (*C.inopinata*)	Okinawa, JP	22/5/2016	2	*Xanthomonadaceae/Xylella*	97.57	98	*Stenotrophomonas panacihumi strain MK06*	98.04	0	2217	NR_117406.1
CiN044	Nematodes (*C.inopinata*)	Okinawa, JP	22/5/2016	6	*Flavobacteriaceae/Chryseobacterium*	97.40	98	*Chryseobacterium rigui strain CJ16*	98.46	0	1485	NR_109537.1
CiN045	Nematodes (*C.inopinata*)	Okinawa, JP	22/5/2016	3	*Comamonadaceae/Pseudacidovorax*	99.67	99	*Pseudacidovorax intermedius strain CC-21*	99.83	0	2217	NR_044241.1
CiN046	Nematodes (*C.inopinata*)	Okinawa, JP	22/5/2016	2	*Xanthomonadaceae/-*	97.61	98	*Stenotrophomonas panacihumi strain MK06*	98.02	0	2108	NR_117406.1
CiN047	Nematodes (*C.inopinata*)	Okinawa, JP	22/5/2016	1	*Enterobacteriaceae/unclassified*	97.82	98	*Enterobacter oryziphilus strain REICA_142*	99.59	0	1339	NR_125587.1
CiN048	Nematodes (*C.inopinata*)	Okinawa, JP	22/5/2016	2	*Xanthomonadaceae/Pseudoxanthomonas*	97.10	98	*Pseudoxanthomonas spadix strain IMMIB AFH-5*	98.02	0	2100	NR_042580.1
CiN049	Nematodes (*C.inopinata*)	Okinawa, JP	22/5/2016	4	*Xanthomonadaceae/Pseudoxanthomonas*	93.06	97	*Pseudoxanthobacter soli strain CC4*	99.83	0	2152	NR_044225.1
CiN050	Nematodes (*C.inopinata*)	Okinawa, JP	22/5/2016	6	*Flavobacteriaceae/Chryseobacterium*	97.37	99	*Chryseobacterium hagamense strain RHA2-9*	98.68	0	1616	NR_115852.1
CiN051	Nematodes (*C.inopinata*)	Okinawa, JP	22/5/2016	5	*Sphingomonadaceae/Sphingobium*	97.97	99	*Sphingobium rhizovicinum strain CC-FH12-1*	98.98	0	2115	NR_044226.1
CiN052	Nematodes (*C.inopinata*)	Okinawa, JP	22/5/2016	6	*Flavobacteriaceae/Chryseobacterium*	96.92	99	*Chryseobacterium hagamense strain RHA2-9*	98.35	0	1589	NR_115852.1
CiN053	Nematodes (*C.inopinata*)	Okinawa, JP	22/5/2016	3	*Comamonadaceae/Comamonas*	97.54	99	*Pseudacidovorax intermedius strain CC-21*	97.54	0	2019	NR_044241.1
CiN054	Nematodes (*C.inopinata*)	Okinawa, JP	22/5/2016	3	*Comamonadaceae/Acidovorax*	98.31	99	*Acidovorax wautersii strain NF 1078*	98.56	0	2087	NR_109656.1
CiN055	Nematodes (*C.inopinata*)	Okinawa, JP	22/5/2016	3	*Comamonadaceae/Acidovorax*	97.75	99	*Acidovorax wautersii strain NF 1078*	98.37	0	1718	NR_109656.1
CiN056	Nematodes (*C.inopinata*)	Okinawa, JP	22/5/2016	4	*Xanthobacteraceae/Azorhizobium*	99.02	99	*Azorhizobium oxalatiphilum strain NS12*	100	0	2073	NR_108517.1
CiN057	Nematodes (*C.inopinata*)	Okinawa, JP	22/5/2016	3	*Comamonadaceae/Pseudacidovorax*	96.51	98	*Pseudacidovorax intermedius strain CC-21*	96.75	0	2047	NR_044241.1
CiN058	Nematodes (*C.inopinata*)	Okinawa, JP	22/5/2016	3	*Comamonadaceae/Acidovorax*	98.79	99	*Acidovorax wautersii strain NF 1078*	98.96	0	1033	NR_109656.1
CiN059	Nematodes (*C.inopinata*)	Okinawa, JP	22/5/2016	4	*Xanthobacteraceae/Azorhizobium*	98.94	99	*Azorhizobium oxalatiphilum strain NS12*	99.91	0	2087	NR_108517.1
CiN060	Nematodes (*C.inopinata*)	Okinawa, JP	22/5/2016	3	*Comamonadaceae/Comamonas*	97.30	98	*Pseudacidovorax intermedius strain CC-21*	97.3	0	2006	NR_044241.1
CiN061	Nematodes (*C.inopinata*)	Okinawa, JP	22/5/2016	3	*Comamonadaceae/Comamonas*	97.53	99	*Pseudacidovorax intermedius strain CC-21*	97.53	0	2004	NR_044241.1

Supplementary Table 5. *C. inopinata* cultures esterblished from *Ficus septica* syconia

Isolate name	Isolation source	Isolation location	Host Tree ID	Host Tree Sex	Isolation date	18S rRNA match
OKI25-1	Fig syconium	Okinaw a island, Okinaw a	OKI25	Male	15/5/2016	1547/1548
OKI27-1	Fig syconium	Okinaw a island, Okinaw a	OKI27	Female	15/5/2016	1547/1548
OKI7-1	Fig syconium	Okinaw a island, Okinaw a	OKI7	Male	13/5/2015	1547/1548
OKI10-1	Fig syconium	Okinaw a island, Okinaw a	OKI10	Female	13/5/2015	1547/1548
IS165-1	Fig syconium	Ishigaki island, Okinaw a	IS165	Unknow n	13/5/2015	1548/1548
IS174-1	Fig syconium	Ishigaki island, Okinaw a	IS174	Unknow n	13/5/2015	1548/1548
IR43-1	Fig syconium	Iriomote island, Okinaw a	IR43	Male	18/5/2016	1548/1548
IR48-1	Fig syconium	Iriomote island, Okinaw a	IR48	Male	18/5/2016	1548/1548
IR1-1	Fig syconium	Iriomote island, Okinaw a	IR1	Female	15/5/2015	1548/1548
IR2-1	Fig syconium	Iriomote island, Okinaw a	IR2	Male	15/5/2015	1548/1548
MY62-1	Fig syconium	Miyako island, Okinaw a	MY62	Unknow n	21/5/2016	1547/1548
MY70-1	Fig syconium	Miyako island, Okinaw a	MY70	Unknow n	21/5/2016	1547/1548
YON30-1	Fig syconium	Yonaguni island, Okinaw a	YON30	Unknow n	17/5/2016	1548/1548
BRC20404	Fig syconium	Taiw an island, Taiw an	TA2	Unknow n	10/7/2015	1548/1548
BRC20403	Fig syconium	Taiw an island, Taiw an	TA8	Unknow n	10/19/2015	1548/1548
BRC20395	Fig syconium	Taiw an island, Taiw an	TA11	Unknow n	10/19/2015	1548/1548

Supplementary Table 6. Coexistence of nematodes and wasps in *Ficus septica* syconia

		Nematode		
		Positive (n)	Negative (n)	Total
Wasp	Positive	209	93	302
	Negative	4	17	21
Total		213	110	323

Supplementary Table 7. *C. inopinata* numbers detected from two fig wasp species

	C. bisulcatus	*Philotrypesis* sp.
Number of nematodes		
0	2	30
1-3	9	6
4-6	6	1
7-9	1	0
>=10	1	0
Total	19	37

Supplementary Table 8. DNA and RNA sequencing libraries and data

Species	Library name	Library type	Sample type	Accession number	Sequencing platform	Maximum read length (bp)	Number of reads	Sequenced bases	Estimated insert size (bp)
**genomic DNA libraries used in assembly**									
C. inopinata	lg3119_350b	pair-end	mixed-stage	DRR093016	HiSeq 2500	101	87,386,594	8826 M	350
C. inopinata	lg3119_3k	mate-pair	mixed-stage	DRR093017	HiSeq 2500	101	86,887,604	8776 M	3200
C. inopinata	lg3119_8k	mate-pair	mixed-stage	DRR093018	HiSeq 2500	101	79,864,078	8066 M	8000
C. inopinata	pcrfree-sp34	pair-end	mixed-stage	DRR093019	MiSeq	301	44,675,294	13289 M	588
C. inopinata	SP34-3k_S1	mate-pair	mixed-stage	DRR093020, DRR093030	MiSeq	301	24,386,650	7202 M	3200
C. inopinata	SP34-5k_S2	mate-pair	mixed-stage	DRR093021, DRR093031	MiSeq	301	18,613,116	5376 M	5100
C. inopinata	SP34-8k_S3	mate-pair	mixed-stage	DRR093022, DRR093032	MiSeq	301	26,320,818	7160 M	7900
C. inopinata	SP34-12k_S4	mate-pair	mixed-stage	DRR093023, DRR093033	MiSeq	301	32,054,714	9195 M	12000
C. inopinata	sp34Pac	SMRT	mixed-stage	DRR093029	PacBio	20k	1,807,941	15200 M	N50 length = 11.0K
**genomic DNA libraries used for remapping**									
C. inopinata	sp34-fa_S1	pair-end	virgin adult female	DRR093024	MiSeq	301	13,387,066	3153 M	248
C. inopinata	sp34-fa_S2	pair-end	virgin adult female	DRR093025	MiSeq	301	6,794,992	1428 M	149
C. inopinata	sp34-ma_S3	pair-end	adult male	DRR093026	MiSeq	301	9,058,964	2177 M	259
C. inopinata	sp34-ma_S4	pair-end	adult male	DRR093027	MiSeq	301	16,801,452	3938 M	246
C. inopinata	sp34_mix_S5	pair-end	mixed-stage	DRR093028	MiSeq	301	6,710,620	1804 M	306
**RNAseq libraries**									
C. inopinata	sp34.M1	polyA-stranded	adult male	DRR093034	HiSeq 2500	101	43,175,684	4361 M	~200
C. inopinata	sp34.M2	polyA-stranded	adult male	DRR093035	HiSeq 2500	101	46,559,244	4702 M	~200
C. inopinata	sp34.F1	polyA-stranded	virgin adult female	DRR093036	HiSeq 2500	101	46,063,462	4653 M	~200
C. inopinata	sp34.F2	polyA-stranded	virgin adult female	DRR093037	HiSeq 2500	101	45,551,712	4561 M	~200
C. inopinata	sp34.L1	polyA-stranded	2nd-3rd stage larvae	DRR093038	HiSeq 2500	101	45,040,328	4549 M	~200
C. inopinata	sp34.L2	polyA-stranded	2nd-3rd stage larvae	DRR093039	HiSeq 2500	101	44,529,780	4497 M	~200
C. inopinata	lr1267	polyA-stranded	mixed-stage	DRR093040	HiSeq 2500	101	40,206,416	4061 M	~200

Supplementary Table 9. Statistics of genome assemblies and annotations of 9 *Caenorhabditis* species and *P. pacificus*

	C. inopinata	***C. elegans***	***C. briggsae***	***C. tropicalis***	***C. brenneri***	***C. remanei***	***C. sinica***	***C. japonica***	***C. angaria***	***P. pacificus***
Assembly size (Mb)	123.0	100.3	108.4	79.3	190.4	145.4	131.8	166.3	106.0	172.5
Num. scaffolds	6+1	6+1	367	665	3,305	3,670	15,261	18,817	34,621	18,083
Average (kb)	17,573	14,327	295	119	58	40	9	9	3	10
Largest scaff (kb)	23,638	20,924	21,541	33,335	4,147	4,501	384	1,087	868	5,268
N50 (kb)	20,595	17,494	17,485	20,922	382	436	25	94	80	1,245
N50 (n)	3	3	3	2	120	70	1,291	429	354	39
N90 (kb)	18,191	13,784	14,579	82	27	12	5	2	1	86
N90 (n)	6	6	6	100	823	938	5,841	5,561	5,877	174
Valid nuc (bp)	122,594,527	100,286,401	105,416,539	76,497,192	170,093,638	138,406,203	130,391,159	154,057,934	94,555,035	153,192,245
Gaps (bp)	413,509	0	2,967,626	2,824,241	20,276,083	7,036,533	1,406,227	12,198,257	11,442,593	19,302,620
Num. coding genes	21,608	20,247	21,814	22,326	30,660	31,437	34,696	29,931	27,970	24,216
Coding gene coverage	53.7%	63.1%	59.7%	50.9%	37.2%	47.9%	51.4%	40.1%	43.8%	54.6%
Coding gene size (median; bp)	1,992.0	1,972.0	1,964.0	1,250.0	1,548.0	1,465.0	1,445.0	1,059.0	798.5	2,561.0
Coding gene size (mean; bp)	3,054.5	3,124.2	2,967.5	1,808.7	2,311.4	2,214.9	1,952.2	2,227.7	1,658.5	3,891.6
Coding gene size (maximum; bp)	64,222	102,626	89,932	35,749	166,937	59,124	30,321	48,334	43,526	113,978
Total coding genes (Mb)	66.0	63.3	64.7	40.4	70.9	69.6	67.7	66.7	46.4	94.2
Intergenic space (median; bp)	1,431.5	925.0	1,183.0	847.0	1,269.0	927.0	721.0	1,611.5	1,293.0	1,565.0
Intergenic space (mean; bp)	2,771.8	2,209.5	2,394.6	1,716.2	3,249.9	2,046.3	1,368.4	3,047.4	2,602.4	3,386.5
Intergenic space (maximum; bp)	88,632	70,672	39,123	61,106	102,000	59,888	31,806	53,894	62,690	167,971
Total intergenic space (Mb)	57.0	37.0	43.6	38.9	119.5	75.8	64.1	99.6	59.6	78.3
Num. CDS	122,297	123,707	121,319	109,781	159,252	157,399	175,648	107,451	88,287	249,850
CDS coverage	21.7%	25.0%	24.1%	30.6%	19.3%	26.0%	29.9%	15.6%	21.5%	14.0%
CDS size (median; bp)	144.0	146.0	151.0	155.0	156.0	158.0	154.0	160.0	186.0	85.0
CDS size (mean; bp)	217.7	202.4	215.6	221.0	230.4	240.4	224.4	241.1	257.7	96.7
CDS size (maximum; bp)	11,954	14,975	17,127	12,092	15,713	16,340	14,282	14,600	8,254	12,279
Total CDS (Mb)	26.6	25.0	26.2	24.3	36.7	37.8	39.4	25.9	22.7	24.1
CDS sum per gene (median; bp)	918.0	993.0	933.0	843.0	942.0	972.0	924.0	577.5	564.0	723.0
CDS sum per gene (mean; bp)	1,232.2	1,236.4	1,199.0	1,086.7	1,196.8	1,203.6	1,135.8	865.4	813.4	997.3
CDS sum per gene (maximum; bp)	51,234	55,689	40,743	22,458	67,152	41,766	23,808	28,116	22,665	24,240
Num. introns	100,688	103,460	99,505	87,455	128,592	125,962	140,952	77,520	60,317	225,634
Intron size (median; bp)	72.0	63.0	55.0	48.0	54.0	52.0	53.0	76.0	96.0	141.0
Intron size (mean; bp)	391.2	309.1	337.4	182.6	257.6	247.3	199.2	512.5	388.9	309.3
Intron size (maximum; bp)	20,056	100,912	82,249	14,053	32,133	29,124	6,631	28,426	14,582	105,802
Intron sum per gene (median; bp)	859.0	704.0	673.0	287.0	448.0	361.0	419.0	289.0	99.0	1,708.0
Intron sum per gene (mean; bp)	1,822.7	1,579.2	1,539.0	715.4	1,080.4	990.7	809.1	1,327.5	838.7	2,881.6
Intron sum per gene (maximum; bp)	57,004	101,564	87,061	33,190	49,900	49,923	22,043	44,365	39,477	112,985
Protein similarity (vs. *C. elegans*)	81.3%	NA	80.4%	80.1%	79.5%	79.7%	80.3%	81.3%	79.7%	83.3%
Num. protein alignments for similarity calculation	16,140	NA	16,211	15,959	10,499	14,361	16,433	14,534	10,729	5,511
Synteny coverage (vs. *C. elegans*)	76.3%	NA	68.7%	NA	NA	NA	NA	NA	NA	NA
Complete BUSCOs (protein)	98.1%	99.6%	98.4%	90.3%	95.9%	97.4%	91.5%	89.6%	63.5%	72.2%
Cegma completeness: complete/partial (%)	98.8/100	98.4/100	99.2/99.6	97.6/99.6	99.2/100	96.0/99.2	96.8/100	80.7/96.8	79.4/95.2	88.3/94.8
Average CEG number: complete/partial	1,10/1.16	1.11/1.20	1.12/1.20	1.16/1.22	1.71/1.85	1.19/1.29	1.44/1.59	1.24/1.56	1.25/1.56	1.22/1.34

Supplementary Table 10. Statistics of repeats in the genomes

	C. inipinata		*C. elegans*		*C. briggsae*	
	num element	% in bp	num element	% in bp	num element	% in bp
SINEs:	79	0.01	717	0.15	0	0
LINEs:	7475	1.63	1819	0.49	2219	0.52
LINE/CR1	1317	0.22	888	0.32	1952	0.46
LINE/RTE-RTE	5499	1.27	31	0.02	0	0
LTR element:*	8133	2.10	928	0.24	714	0.41
LTR/Pao	4617	1.20	785	0.20	232	0.11
LTR/Gypsy	2656	0.70	89	0.03	320	0.19
DNA element:*	83488	16.42	50026	9.30	97524	19.36
DNA/TcMar-Tc1	47213	8.85	7013	1.31	14091	3.04
DNA/TcMar-Tc2	1266	0.22	9	0.006	31617	5.55
DNA/hAT	11816	2.14	6083	0.81	5350	1.12
DNA/MULE-MuDR	334	0.13	1639	0.39	5775	1.10
Small RNA:	18	0.003	0	0	0	0
Satellites:	104	0.04	2080	0.50	5819	1.28
Simple repeat:	26865	1.21	22177	1.15	22109	1.89
Low complexity:	5651	0.24	6027	0.28	5935	0.28
Unclassified:	52759	9.34	18297	3.98	14623	2.85
TOTAL	36210727 bp (29.53%)		17306776 bp (17.26%)		28866290 bp (27.38 %)	

*LTR elements and DNA transposases were further refined by LTR harvest/digest and MGE scan. The results were shown in Supplementary Table 3.4.

Supplementary Table 11. LTR retrotransposon elements and DNA transposases detected in genomes

		C. inopinata	*C. elegans*	*C. briggsae*
LTR element:		641	62	128
	Full	104	10	10
	Partial	315	44	36
	LTR only	222	8	82
DNA transposase:		702	74*	NA**

* Number was obtained from "Bessereau JL. Transposons in *C. elegans.* WormBook. 2006;18:1-3."
**Not analysed in this study

Supplementary Table 12. Highly represented pfam domains in *C. inopinata* , *C. elegans* and *C. briggsae*

	C. inopinata	*C. elegans*	*C. briggsae*
DDE_3	505	1	8
RVT_1	440	2	26
HTH_Tnp_Tc3_2	338	0	5
WD40	321	318	323
Collagen	321	262	259
Transposase_1	282	0	2
Pkinase	276	290	328
I-set	213	255	257
DUF1759	196	0	8
RRM_1	190	157	165
Col_cuticle_N	185	162	152
rve	181	0	26
Peptidase_A17	163	1	4
zf-C2H2	159	34	33
ShK	153	287	238
Kunitz_BPTI	146	138	132
Lustrin_cystein	136	140	136
zf-C4	134	270	248
Hormone_recep	128	265	247
7tm_1	124	128	126
WSN	118	53	27
fn3	118	137	131
Ank_2	118	123	121
HTH_29	111	0	4
Y_phosphatase	108	103	90
F-box	105	253	124
DUF1174	105	72	80
FTH	103	250	124
Pkinase_Tyr	102	118	117
HTH_Tnp_Tc3_1	100	0	7
Mito_carr	97	107	103
T-box	95	22	39
Ldl_recept_a	95	107	102
Homeobox	95	97	139
MFS_1	94	109	110
ABC_tran	94	88	103
Ion_trans_2	93	97	95
Laminin_EGF	89	90	83
LRR_8	88	94	92
Neur_chan_LBD	87	102	105
Lectin_C	85	222	145
Helicase_C	84	82	76
DUF1758	84	0	4
zf-CCHC	82	33	29
EGF_CA	82	77	83
TSP_1	80	89	80
Motile_Sperm	80	114	68
Spectrin	77	76	76
Phlebovirus_G2	76	1	6
TTR-52	75	59	58
Neur_chan_memb	75	78	83
7TM_GPCR_Srw	75	158	151
CUB	68	85	82
Cadherin	66	69	66
EB	65	79	70
7TM_GPCR_Srx	61	109	124
7TM_GPCR_Str	58	237	244
ABC_membrane	54	56	68
BTB	50	108	132
Recep_L_domain	49	121	120
UDPGT	47	76	95
p450	41	78	86
7TM_GPCR_Srh	40	245	174
Glyco_transf_92	38	64	66
DUF19	20	97	81
FBA_2	17	180	86
7TM_GPCR_Srbc	14	87	48
MATH	6	158	64
7TM_GPCR_Srz	0	79	51

Top 50 highly represented pfam domains in the genomes of *C. inopinata* , *C. elegans* and *C. briggsae* were shown.

Pfam domains highly or lowely representing in *C. inopinata* compared to *C. elegans* (> 2-fold and FDR < 0.01) were marked in green and orange, respectively.

Supplementary Table 13. CAZymes detected in the genomes

Glycoside Hydrolase Family

	C. inopinata	*C. elegans*	*C. briggsae*
GH1	1	2	1
GH2	2	2	2
GH13	4	4	4
GH15	0	1	1
GH18	30	35	31
GH19	2	4	4
GH20	5	5	5
GH25	6	4	3
GH27	1	1	1
GH29	1	1	1
GH30	3	4	5
GH31	5	4	5
GH35	2	2	2
GH37	6	5	5
GH38	3	3	3
GH47	7	7	7
GH56	2	1	1
GH59	1	1	0
GH63	1	1	1
GH84	1	1	1
GH85	1	1	1
GH89	1	1	1
GH116	2	2	2
GH133	1	1	1
	88	93	88

GlycosylTransferase family

	C. inopinata	*C. elegans*	*C. briggsae*
GT1	43	70	68
GT2	3	3	3
GT3	1	1	1
GT4	4	4	4
GT7	4	4	4
GT8	4	4	4
GT10	3	5	5
GT11	12	23	8
GT13	3	4	4
GT14	15	20	15
GT16	1	1	1
GT18	2	1	1
GT20	2	2	2
GT21	2	3	2
GT22	3	3	4
GT23	1	1	1
GT24	2	2	2
GT25	1	1	1
GT27	12	9	10
GT28	1	1	1

GT31	14	27	14
GT33	1	1	1
GT35	1	1	1
GT41	1	1	1
GT43	2	7	6
GT47	1	1	1
GT49	3	9	5
GT50	1	1	1
GT57	2	2	2
GT58	1	1	1
GT59	1	2	2
GT61	1	1	1
GT64	1	1	2
GT65	1	1	1
GT66	1	1	1
GT68	2	1	1
GT75	7	8	1
GT76	1	1	1
GT77	1	1	1
GT92	31	55	49
	193	285	234

Carbohydrate-Binding Module family

	C. inopinata	*C. elegans*	*C. briggsae*
CBM13	2	2	2
CBM14	6	8	13
CBM20	1	1	0
CBM21	2	2	2
CBM57	1	1	1
	12	14	18

Auxiliary Activities family

	C. inopinata	*C. elegans*	*C. briggsae*
AA1	0	0	1
AA3	1	1	1
AA4	2	2	2
subtotal	3	3	4

Carbohydrate Esterase family

	C. inopinata	*C. elegans*	*C. briggsae*
CE1	4	2	3
CE9	1	1	1
CE10	36	51	50
CE14	1	1	1
	42	55	55

Supplementary Table 14. GO enrichment of significantly expanded/contracted gene families in *C. inopinata*

C. inopinata expanded gene families

GO Term	**Description**	**GO class**	**P-value**	**FDR**
GO:0008270	zinc ion binding	MF	2.00E-52	3.40E-49
GO:0004190	aspartic-type endopeptidase activity	MF	6.40E-30	5.40E-27
GO:0003700	sequence-specific DNA binding transcription factor activity	MF	7.80E-18	4.80E-15
GO:0046983	protein dimerization activity	MF	1.20E-13	6.10E-11
GO:0008528	G-protein coupled peptide receptor activity	BP	1.60E-11	7.10E-09
GO:0015074	DNA integration	MF	7.10E-11	3.00E-08
GO:0004652	polynucleotide adenylyltransferase activity	MF	2.40E-09	9.00E-07

C. inopinata contracted gene families

GO Term	**Description**	**GO class**	**P-value**	**FDR**
GO:0038022	G-protein coupled olfactory receptor activity	MF	1.10E-25	2.30E-22
GO:0042048	olfactory behavior	BP	1.70E-25	2.30E-22
GO:0033692	cellular polysaccharide biosynthetic process	BP	5.55E-15	1.93E-12
GO:0050911	detection of chemical stimulus involved in sensory perception of	BP	8.30E-10	2.50E-07
GO:0005887	integral to plasma membrane	CC	5.40E-09	1.40E-06

MF: molecular function; BP: biological process; CC: cellular component

Supplementary Table 15. Orthologues in the Insulin/insulin-like growth factor signaling pathway

Gene	OrthoGroup ID	*C. inopinata*	*C. elegans*	*C. briggsae*
age-1	OG0003277	Sp34_20327500.t1	B0334.8a	CBG02868
ist-1	OG0006659	Sp34_X0200110.t1	C54D1.3	CBG10979
akt-1	OG0001569	Sp34_50284700.t1	C12D8.10b, F28H6.1a	CBG09565
akt-2	OG0001569	Sp34_50284700.t1	C12D8.10b, F28H6.1a	CBG09565
ddl-1	OG0003047	Sp34_20112400.t1	F59E12.10	CBG02459
pdk-1	OG0005502	Sp34_X0009000.t1	H42K12.1b	CBG08059
hsb-1	OG0007854	Sp34_40031400.t1	K08E7.2	CBG13509
par-5 (ftt-1)	OG0012491		M117.2a	CBG06174
ftt-2	OG0009297	Sp34_X0091400.t1	F52D10.3a	CBG17289
daf-16	OG0007511	Sp34_10345800.t1	R13H8.1h	CBG12825
daf-18	OG0001713	Sp34_40020320.t1, Sp34_50398500.t1	T07A9.6	CBG13540
skn-1	OG0001893	Sp34_40226700.t1	T19E7.2a, W02H5.7a	CBG19887
pptr-1	OG0011386	Sp34_50380400.t1	W08G11.4	CBG22810
sgk-1	OG0008973	Sp34_X0041300.t1	W10G6.2b	CBG16386
aap-1	OG0006577	Sp34_10257700.t1	Y110A7A.10	CBG10815
ddl-2	OG0010686	Sp34_20069300.t1	Y48E1B.1	CBG20761
hsf-1	OG0009877	Sp34_10030000.t1	Y53C10A.12	CBG18699
daf-2	OG0008758	Sp34_30289300.t1	Y55D5A.5c	CBG15732

Supplementary Table 16. Orthologues in the dauer formation pathways

Gene	Orthogroup ID	*C. inopinata*	*C. elegans*	*C. briggsae*
age-1	OG0003266	Sp34_20327500.t1	B0334.8a	CBG02868
akt-1	OG0001575	Sp34_50284700.t1	C12D8.10b, F28H6.1a	CBG09565
bra-1	OG0005231	Sp34_X0124400.t1	F54B11.6	CBG07410
cam-1	OG0010371	Sp34_20301200.t1	C01G6.8a	CBG20223
daf-10	OG0010938	Sp34_40353100.t1	F23B2.4	CBG21783
daf-11	OG0011470	Sp34_50149900.t1	B0240.3a	CBG23280
daf-12	OG0004149	Sp34_X0074510.t1	F11A1.3a	CBG04512
daf-14	OG0004108	Sp34_40089710.t1	F01G10.8b	CBG04415
daf-15	OG0010875	Sp34_40346100.t1	C10C5.6c	CBG21644
daf-16	OG0007450	Sp34_10345800.t1	R13H8.1h	CBG12825
daf-18	OG0001721	Sp34_40020320.t1, Sp34_50398500.t1	T07A9.6	CBG13540
daf-19	OG0002409	Sp34_20211010.t1	F33H1.1b	CBG00885
daf-1	OG0002646	Sp34_40119100.t1	F29C4.1a	CBG01651
daf-21	OG0004168	Sp34_50349700.t1	C47E8.5	CBG04560
daf-28	OG0000212	Sp34_20122500.t1, Sp34_X0004000.t1	Y116F11B.1, ZK75.1, ZK84.6	CBG02554
daf-2	OG0008682	Sp34_30289300.t1	Y55D5A.5c	CBG15732
daf-36	OG0006122	Sp34_50283420.t1	C12D8.5	CBG09553
daf-3	OG0005500	Sp34_X0016200.t1	F25E2.5d	CBG08108
daf-4	OG0005820	Sp34_30131600.t1	C05D2.1a	CBG08963
daf-5	OG0010613	Sp34_20065100.t1	W01G7.1	CBG20832
daf-6	OG0005293	Sp34_X0140500.t1	F31F6.5	CBG07607
daf-7	OG0011879	Sp34_30330900.t1	B0412.2	CBG24910
daf-8	OG0007289	Sp34_10314800.t1	R05D11.1	CBG12513
daf-9	OG0001603	Sp34_X0228700.t1	T13C5.1a	CBG10440, CBG14860
eak-4	OG0000566	Sp34_40025700.t1, Sp34_50268500.t1	F14H8.2, F14H8.4, F14H8.5, F53B2.3	CBG13526
eak-6	OG0012132	Sp34_10058500.t1	F10G8.4b, Y44A6D.4	
egl-4	OG0005637	Sp34_40042420.t1	F55A8.2h	CBG08401
ftt-2	OG0000956	Sp34_X0091400.t1	F52D10.3a, M117.2a	CBG06174, CBG17289
hil-1	OG0006868	Sp34_50345510.t1	C30G7.1	CBG11603
hsp-12.6	OG0000836	Sp34_40344800.t1, Sp34_40344900.t1	F38E11.1, F38E11.2	CBG21659, CBG21660
ins-1	OG0005619	Sp34_40077810.t1	F13B12.5	CBG08370
ins-4	OG0000658	Sp34_20122500.t1, Sp34_X0004000.t1	Y116F11B.1, ZK75.1, ZK84.6	CBG02554
ins-6	OG0000658	Sp34_20122500.t1, Sp34_X0004000.t1	Y116F11B.1, ZK75.1, ZK84.6	CBG02554
ins-7			ZK1251.2b	
ins-9			C06E2.8	
ins-18	OG0006935	Sp34_10100500.t1	T28B8.2	CBG11840
ins-19			T10D4.13	
ins-22	OG0000850	Sp34_30206400.t1	M04D8.1, M04D8.2, M04D8.3	CBG25894, CBG25895
ins-31			T10D4.4	
let-363	OG0007458	Sp34_10277510.t1	B0261.2	CBG12850
mrp-1	OG0000147	Sp34_50408800.t1, Sp34_X0019810.t1, Sp34_X0019900.t1, Sp34_X0038300.t1, Sp34_X0061510.t1	E03G2.2, F21G4.2, F57C12.4, F57C12.5b, Y43F8C.12	CBG01916, CBG08145, CBG08146, CBG08354, CBG15993, CBG23578
nap-1	OG0010783	Sp34_40064700.t1	D2096.8	CBG21270
ncr-1	OG0008134	Sp34_X0255400.t1	F02E8.6	CBG14473
ncr-2	OG0009029	Sp34_30233000.t1	F09G8.4	CBG16653
par-5	OG0000956	Sp34_X0091400.t1	F52D10.3a, M117.2a	CBG06174, CBG17289
pdk-1	OG0005462	Sp34_X0009000.t1	H42K12.1b	CBG08059
rle-1	OG0009649	Sp34_30109300.t1	M142.6a	CBG18273
sdf-9	OG0012132	Sp34_10058500.t1	F10G8.4b, Y44A6D.4	
sod-3	OG0001685	Sp34_10337800.t1	C08A9.1, F10D11.1	CBG12726
tax-2	OG0007225	Sp34_10117200.t1	F36F2.5	CBG12422
tax-4	OG0005054	Sp34_30262300.t1	ZC84.2	CBG06949
tbx-2	OG0010792	Sp34_30025300.t1	F21H11.3	CBG21316
unc-31	OG0011151	Sp34_40152500.t1	ZK897.1j	CBG22310

Supplementary Table 17. Orthologues in the sex determination pathway

Gene	OrthoGroup ID	*C. inopinata*	*C. elegans*	*C. briggsae*
fem-3	OG0000053	Sp34_40176300.t1, Sp34_40178000.t1, Sp34_40178500.t1, Sp34_40276700.t1, Sp34_40276800.t1, Sp34_40276900.t1, Sp34_40320600.t1, Sp34_40320700.t1, Sp34_40320900.t1, Sp34_40321100.t1, Sp34_40321200.t1, Sp34_40321400.t1, Sp34_40321600.t1, Sp34_40331100.t1, Sp34_40331200.t1, Sp34_40331300.t1, Sp34_40331400.t1, Sp34_40331500.t1, Sp34_40352600.t1, Sp34_40352900.t1	C01F6.4a, C01F6.5, F23B2.6	CBG21774, CBG21775, CBG21776
fbf-1	OG0000259	Sp34_20124000.t1, Sp34_20228000.t1	F21H12.5, H12I13.4, Y45F10A.2, Y73B6BL.38	CBG01774, CBG02701, CBG02702, CBG09894, CBG13460
fbf-2	OG0000259	Sp34_20124000.t1, Sp34_20228000.t1	F21H12.5, H12I13.4, Y45F10A.2, Y73B6BL.38	CBG01774, CBG02701, CBG02702, CBG09894, CBG13460
fem-1	OG0010265	Sp34_40329500.t1	F35D6.1a	CBG19924
fem-2	OG0008534	Sp34_30327800.t1	T19C3.8	CBG15267
fog-1	OG0001759	Sp34_10173000.t1	Y54E10A.4b	CBG14960, CBG22257
fog-2	OG0000005	Sp34_30287000.t1, Sp34_40271920.t1, Sp34_50399100.t1	B0391.11a, B0391.5, B0391.6, B0391.9, B0511.3, C02H6.2, C06H5.1, C06H5.2, C08E3.10a, C08E3.12, C08E3.6, C08E3.7, C08E3.8, C08E3.9, C08F11.5a, C17B7.11, C17C3.5, C17C3.6, C18B12.5, C25D7.4a, C29F9.10, C29F9.11, C31C9.3, C31C9.4, C33E10.2, C36C9.3, C38D9.1, C38D9.6, C38D9.7, C38D9.9, C39B5.3, C39B5.4, C39B5.7, C39B5.8, C39B5.9, F07G6.6, F07G6.7, F09C6.15, F09C6.2, F09C6.6, F10A3.17, F10A3.2, F10A3.3, F14D2.13b, F14H3.7, F28F8.4, F28F8.8a, F31E9.3, F31F4.15a, F35E12.3, F36G9.14, F42G2.8, F44E7.6, F44G3.12, F44G3.14, F44G3.8a, F45C12.8, F47H4.4, F47H4.8, F47H4.9, F52D2.1, F52D2.10, F52D2.8b, F54B8.3, F54D10.2, F56C3.2, F57G4.10, F57G4.8, F59A1.7, H03G16.4, K03D7.7, M162.11, M162.8, T05H4.2, T06C12.4, T06E6.13, T06E6.15, T08E11.7, T09F5.11, T12B5.1, T12B5.10, T12B5.11, T12B5.13, T12B5.2, T12B5.3, T12B5.4, T12B5.5, T12B5.6b, T12B5.7, T12B5.8, T13F3.5, T20H9.1, T20H9.2, T20H9.3, T20H9.4, T24C2.4, T25E12.12, T28A11.21, T28C12.3, W04E12.1, Y102A5C.1, Y102A5C.13, Y102A5C.14, Y102A5C.19, Y102A5C.9, Y113G7B.1b, Y113G7B.3, Y113G7B.4, Y113G7B.5b, Y113G7B.6, Y113G7B.7, Y119D3A.1, Y119D3A.2, Y119D3A.3, Y119D3A.4, Y119D3B.18, Y119D3B.19, Y119D3B.20, Y119D3B.22, Y119D3B.4, Y119D3B.6, Y119D3B.7, Y119D3B.8, Y119D3B.9, Y22D7AR.11, Y22D7AR.2, Y22D7AR.9, Y36E3A.1, Y37H2A.12a, Y37H2A.18, Y37H2A.4, Y37H2A.5b, Y37H2A.6, Y37H2A.7, Y37H2C.3, Y38A10A.4, Y45F10C.3, Y47D3A.2, Y47H9C.10, Y47H9C.12, Y54F10BL.1, Y54F10BM.10, Y54F10BM.11, Y54F10BM.15, Y54F10BM.20, Y54F10BM.4, Y54F10BM.5, Y54F10BM.7, Y57G11C.499, Y57G11C.7, Y59A8B.11b, Y59E1A.1, Y61B8A.4, Y67A10A.4, Y67A10A.5, Y73B3A.15, Y73B3A.22, Y75B8A.21, Y82E9BL.10, Y82E9BL.11, Y82E9BL.13, Y82E9BL.14, Y82E9BL.15, Y82E9BL.16, Y82E9BL.17, Y82E9BL.18, Y82E9BL.19, Y82E9BL.4, Y82E9BL.7, Y82E9BL.8, Y82E9BR.12, Y9C9A.12, Y9C9A.8, ZC47.13a, ZC47.14, ZC47.3, ZC47.4, ZC47.5, ZC47.6, ZC47.7, ZK1290.9, cTel54X.1	CBG26930, CBG27190, CBG27308
fog-3	OG0007200	Sp34_10122100.t1	C03C11.2	CBG12385
fox-1	OG0002883	Sp34_X0101100.t1	T07D1.4	CBG02108
gld-1	OG0002174	Sp34_10106600.t1	T23G11.3	CBG00303
her-1	OG0012852	Sp34_50253300.t1 "LTR in the middle"	ZK287.8a	CBG19345
laf-1	OG0006264	Sp34_30210610.t1	Y71H2AM.19b	CBG09816
mab-3	OG0001273	Sp34_20184610.t1	Y53C12B.5a	CBG01044, CBG01045
mag-1	OG0009785	Sp34_10030700.t1	R09B3.5	CBG18690
mog-1	OG0006326	Sp34_30205000.t1	K03H1.2	CBG09937
mog-4	OG0010629	Sp34_20059500.t1	C04H5.6a	CBG20862
mog-5	OG0003011	Sp34_20102110.t1	EEED8.5	CBG02387
nos-3	OG0002489	Sp34_20184300.t1	Y53C12B.3b	CBG01040
sdc-1	OG0011689	Sp34_10227600.t1	F52E10.1	CBG23946
sdc-2	OG0009226	Sp34_X0090300.t1	C35C5.1	CBG17307
sdc-3	OG0006853	Sp34_50268010.t1	C25D7.3	CBG11582
sex-1	OG0001123	Sp34_X0067600.t1	F44A6.2	CBG17178, CBG19535, CBG19536
tra-1	OG0007621	Sp34_30096710.t1	Y47D3A.6a	CBG13188b
tra-2	OG0006711	Sp34_20266100.t1	C15F1.3a	CBG11194
tra-3	OG0010866	Sp34_40187810.t1	LLC1.1a	CBG21580
xol-1	OG0001118	Sp34_10015000.t1, Sp34_X0215200.t1, Sp34_X0215500.t1	C18A11.5c	CBG16879

Supplementary Table 18. Orthologues of selected nuclear receptors

Gene	OrthoGroup ID	*C. inopinata*	*C. elegans*	*C. briggsae*
mut-16	OG0001367	Sp34_10181000.t1, Sp34_10211800.t1	CBG03869	B0379.3a
mes-6	OG0001777	Sp34_40068900.t1	CBG15499, CBG15631	C09G4.5
rde-2	OG0011074	Sp34_10091400.t1	CBG21903	F21C3.4a
ekl-1	OG0010552	Sp34_10082500.t1	CBG20475	F22D6.6
mes-3	OG0006554	Sp34_10255000.t1	CBG10773	F54C1.3a
zfp-1	OG0005058	Sp34_30276600.t1	CBG06894	F54F2.2a
mut-2	OG0001669	Sp34_10324700.t1	CBG12597, CBG19180	K04F10.6a
cid-1	OG0010901	Sp34_30026400.t1	CBG21327	K10D2.3
gfl-1	OG0004791	Sp34_40327100.t1	CBG06140	M04B2.3
mes-2	OG0006704	Sp34_20165300.t1	CBG11099	R06A4.7
rha-1	OG0003489	Sp34_20308100.t1	CBG03202	T07D4.3
ekl-6	OG0006356	Sp34_30207500.t1	CBG09896	T16G12.5
ekl-4	OG0005818	Sp34_10005700.t1	CBG08755	Y105E8A.17b
ekl-5	OG0000719	Sp34_X0117500.t1	CBG07347, CBG24290, CBG24291, CBG28097	Y26E6A.1
mut-7	OG0001019	Sp34_30194200.t1	CBG10058, CBG10060	ZK1098.3, ZK1098.8

Supplementary Table 19. Orthologues in the siRNA/miRNA/piRNA related pathways

##RNAi inhibitor related

Gene	Orthogroup	*C. inopinata*	*C. elegans*	*C. briggsae*
adr-1	OG0001883	Sp34_10101200.t1	D2005.1, H15N14.1c	CBG19440
somi-1	OG0004989	Sp34_50156810.t1	M04G12.4a	CBG06725
eri-1	OG0007873	Sp34_40020310.t1	T07A9.5b	CBG13539
adr-2	OG0011836	Sp34_30285210.t1	T20H4.4	CBG24155
eri-3	OG0011032	Sp34_20012700.t1	W09B6.3a	CBG21769
eri-5	OG0009182	Sp34_40037000.t1	Y38F2AR.1a	CBG16814
xrn-1	OG0010717	Sp34_20064300.t1	Y39G8C.1	CBG20837
xrn-2	OG0010742	Sp34_20056900.t1	Y48B6A.3	CBG20900

##Biosyntheis and Nuclear export of small RNA

Gene	Orthogroup	*C. inopinata*	*C. elegans*	*C. briggsae*
xpo-3	OG0009479	Sp34_40103100.t1	C49H3.10b	CBG17706
drh-3	OG0005620	Sp34_10076110.t1	D2005.5	CBG08270
drh-1	OG0004426	Sp34_40172510.t1	F15B10.2a	CBG05400
drsh-1	OG0003792	Sp34_10057200.t1	F26E4.10a	CBG03798
dcr-1	OG0011418	Sp34_30052800.t1	K12H4.8	CBG22974
rde-4	OG0006301	Sp34_30210020.t1	T20G5.11	CBG09811
pash-1	OG0011873	Sp34_10280400.t1	T22A3.5a	CBG24339
xpo-2	OG0011136	Sp34_10249800.t1	Y48G1A.5a	CBG22063
xpo-1	OG0010602	Sp34_50086510.t1	ZK742.1a	CBG20576

##Secondary amplification, uptake and intercellular spread of short interfering (si)RNA

Gene	Orthogroup	*C. inopinata*	*C. elegans*	*C. briggsae*
sid-1	OG0001542	Sp34_50189900.t1, Sp34_50190110.t1	C04F5.1	CBG08682
rsd-3	OG0002162	Sp34_X0180000.t1	C34E11.1	CBG00274
rrf-3	OG0002371	Sp34_20154200.t1	F10B5.7	CBG00730
rsd-6	OG0001374	Sp34_10132700.t1	F16D3.2	CBG04074, CBG07263
ego-1	OG0000481	Sp34_10108100.t1, Sp34_10108210.t1	F26A3.3, F26A3.8a, M01G12.12	CBG00321, CBG19448
rrf-1	OG0000481	Sp34_10108100.t1, Sp34_10108210.t1	F26A3.3, F26A3.8a, M01G12.12	CBG00321, CBG19448
rsd-2	OG0000499	Sp34_20166700.t1	F52G2.2a, F52G2.3, K04G11.3	CBG01755, CBG13172, CBG17112
smg-5	OG0003937	Sp34_10297700.t1	W02D3.8	CBG04046
smg-2	OG0005708	Sp34_10267400.t1	Y48G8AL.6	CBG08494
smg-6	OG0008721	Sp34_30305500.t1	Y54F10AL.2a	CBG15668
sid-2	OG0009741	Sp34_30108500.t1	ZK520.2	CBG18280

##RNA-induced Silencing Complex (RISC)

Gene	Orthogroup	*C. inopinata*	*C. elegans*	*C. briggsae*
ain-2	OG0011214	Sp34_10308300.t1	B0041.2d	CBG22231
prg-2	OG0001646	Sp34_10098600.t1	C01G5.2a, D2030.6	CBG11957
prg-1	OG0001646	Sp34_10098600.t1	C01G5.2a, D2030.6	CBG11957
C04F12.1	OG0001359	Sp34_10182700.t1, Sp34_10183000.t1	C04F12.1	CBG03703
ain-1	OG0008956	Sp34_X0044100.t1	C06G1.4	CBG16346
nrde-3	OG0000406	Sp34_50015000.t1	C14B1.7a, C16C10.3, R04A9.2, T22H9.3, Y49F6A.1	CBG01380, CBG21364
C16C10.3	OG0000406	Sp34_50015000.t1	C14B1.7a, C16C10.3, R04A9.2, T22H9.3, Y49F6A.1	CBG01380, CBG21364
Y49F6A.1	OG0000406	Sp34_50015000.t1	C14B1.7a, C16C10.3, R04A9.2, T22H9.3, Y49F6A.1	CBG01380, CBG21364
T22H9.3	OG0000406	Sp34_50015000.t1	C14B1.7a, C16C10.3, R04A9.2, T22H9.3, Y49F6A.1	CBG01380, CBG21364
ppw-1	OG0000768	Sp34_10261700.t1, Sp34_10263500.t1	C18E3.7a, F56A6.1a, K12B6.1	CBG12094
sago-1	OG0000768	Sp34_10261700.t1, Sp34_10263500.t1	C18E3.7a, F56A6.1a, K12B6.1	CBG12094
sago-2	OG0000768	Sp34_10261700.t1, Sp34_10263500.t1	C18E3.7a, F56A6.1a, K12B6.1	CBG12094
tsn-1	OG0004084	Sp34_20224000.t1	F10G7.2	CBG04292
csr-1	OG0009489	Sp34_40104600.t1	F20D12.1a	CBG17720
alg-1	OG0000969	Sp34_X0117100.t1	F48F7.1b, T07D3.7a	CBG07340, CBG24316
alg-2	OG0000969	Sp34_X0117100.t1	F48F7.1b, T07D3.7a	CBG07340, CBG24316
F55A12.1	OG0000304	Sp34_10244410.t1, Sp34_10259300.t1	F55A12.1, F58G1.1, R06C7.1, Y110A7A.18, ZK1248.7	CBG09416, CBG12321, CBG23746
vig-1	OG0005190	Sp34_20015000.t1	F56D12.5a	CBG07107
F58G1.1	OG0000304	Sp34_10244410.t1, Sp34_10259300.t1	F55A12.1, F58G1.1, R06C7.1, Y110A7A.18, ZK1248.7	CBG09416, CBG12321, CBG23746
rde-1	OG0010178	Sp34_50262400.t1	K08H10.7	CBG19426
R06C7.1	OG0000304	Sp34_10244410.t1, Sp34_10259300.t1	F55A12.1, F58G1.1, R06C7.1, Y110A7A.18, ZK1248.7	CBG09416, CBG12321, CBG23746
ergo-1	OG0000621		R09A1.1a	CBG00904, CBG16098, CBG16129, CBG17887, CBG21165
alg-3/alg-4	OG0001584	Sp34_30204400.t1	T22B3.2b, ZK757.3a	CBG09950
T23D8.7	OG0003780	Sp34_10058800.t1	T23D8.7	CBG03777
ppw-2	OG0000304	Sp34_10244410.t1, Sp34_10259300.t1	F55A12.1, F58G1.1, R06C7.1, Y110A7A.18, ZK1248.7	CBG09416, CBG12321, CBG23746
ZK1248.7	OG0000304	Sp34_10244410.t1, Sp34_10259300.t1	F55A12.1, F58G1.1, R06C7.1, Y110A7A.18, ZK1248.7	CBG09416, CBG12321, CBG23746
rde-2	OG0011074	Sp34_10091400.t1	F21C3.4a	CBG21903
rde-3/mut-2	OG0001669	Sp34_10324700.t1	K04F10.6a	CBG12597, CBG19180
mut-7	OG0001019	Sp34_30194200.t1	ZK1098.3, ZK1098.8	CBG10058, CBG10060
mut-15/rde-5	OG0006910	Sp34_50269400.t1	T01C3.8a	CBG11587
mut-14	OG0001003	Sp34_50206500.t1	C14C11.6, Y38A10A.6, ZC317.1	CBG09254
mut-16	OG0001367	Sp34_10181000.t1, Sp34_10211800.t1	B0379.3a	CBG03869

##26G RNA pathway factors

Gene	Orthogroup	*C. inopinata*	*C. elegans*	*C. briggsae*
alg-3/alg-4	OG0001584	Sp34_30204400.t1	T22B3.2b, ZK757.3a	CBG09950
dcr-1	OG0011418	Sp34_30052800.t1	K12H4.8	CBG22974
drh-3	OG0005620	Sp34_10076110.t1	D2005.5	CBG08270
ergo-1	OG0000621		R09A1.1a	CBG00904, CBG16098, CBG16129, CBG17887, CBG21165
eri-1	OG0007873	Sp34_40020310.t1	T07A9.5b	CBG13539
eri-3	OG0011032	Sp34_20012700.t1	W09B6.3a	CBG21769
eri-5	OG0009182	Sp34_40037000.t1	Y38F2AR.1a	CBG16814
eri-6	OG0001212		C34C12.1, C41D11.1e, F40G12.7, Y47H9C.9, Y61A9LA.12	
eri-7	OG0003909		C41D11.7	CBG03999, CBG16740
eri-9	OG0012891		C26E6.7c	CBG18194
henn-1	OG0009107	Sp34_30231500.t1	C02F5.6b	CBG16658
mut-16	OG0001367	Sp34_10181000.t1, Sp34_10211800.t1	B0379.3a	CBG03869
rde-4	OG0006301	Sp34_30210020.t1	T20G5.11	CBG09811
rrf-3	OG0002371	Sp34_20154200.t1	F10B5.7	CBG00730

##22G RNA pathway factors

Gene	Orthogroup	*C. inopinata*	*C. elegans*	*C. briggsae*
cde-1/cid-1	OG0010901	Sp34_30026400.t1	K10D2.3	CBG21327
csr-1	OG0009489	Sp34_40104600.t1	F20D12.1a	CBG17720
drh-3	OG0005620	Sp34_10076110.t1	D2005.5	CBG08270
ego-1	OG0000481	Sp34_10108100.t1, Sp34_10108210.t1	F26A3.3, F26A3.8a, M01G12.12	CBG00321, CBG19448
ekl-1	OG0010552	Sp34_10082500.t1	F22D6.6	CBG20475
haf-6	OG0005709	Sp34_10267300.t1	Y48G8AL.11a	CBG08495
hpl-2	OG0000807	Sp34_30112400.t1, Sp34_30112500.t1	H04D03.6b, K01G5.2c, W05F2.8a	CBG18315
mes-3	OG0006554	Sp34_10255000.t1	F54C1.3a	CBG10773
mes-4	OG0004986	Sp34_50158400.t1	Y2H9A.1	CBG06706
mut-14	OG0001003	Sp34_50206500.t1	C14C11.6, Y38A10A.6, ZC317.1	CBG09254
mut-15	OG0006910	Sp34_50269400.t1	T01C3.8a	CBG11587
mut-16	OG0001367	Sp34_10181000.t1, Sp34_10211800.t1	B0379.3a	CBG03869
mut-2	OG0001669	Sp34_10324700.t1	K04F10.6a	CBG12597, CBG19180
mut-7	OG0001019	Sp34_30194200.t1	ZK1098.3, ZK1098.8	CBG10058, CBG10060
nrde-1	OG0000742	Sp34_30225000.t1, Sp34_40031700.t1	C14B1.6	CBG09209, CBG14218, CBG24218
nrde-2	OG0010464	Sp34_20217000.t1	T01E8.5	CBG20217
nrde-4	OG0000947	Sp34_40305300.t1	F45E4.10b, F45E4.9	CBG05930, CBG05931
rde-10	OG0008450	Sp34_10169100.t1	Y47G6A.4	CBG14935
rde-11	OG0012239	Sp34_40144920.t1	B0564.11, Y75B8A.10	
mut-8/rde-2	OG0011074	Sp34_10091400.t1	F21C3.4a	CBG21903
rrf-1	OG0000481	Sp34_10108100.t1, Sp34_10108210.t1	F26A3.3, F26A3.8a, M01G12.12	CBG00321, CBG19448
rsd-2	OG0000499	Sp34_20166700.t1	F52G2.2a, F52G2.3, K04G11.3	CBG01755, CBG13172, CBG17112
rsd-6	OG0001374	Sp34_10132700.t1	F16D3.2	CBG04074, CBG07263
set-25	OG0000412	Sp34_30015210.t1	Y43F4B.3	CBG03532, CBG03572, CBG10227, CBG14449, CBG16272, CBG24719
set-32	OG0000293	Sp34_40130100.t1, Sp34_50153210.t1	C41G7.4, C49F5.2, R11E3.4, Y108F1.3, Y24D9A.2	CBG01676, CBG01750, CBG09666
wago-10	OG0000406	Sp34_50015000.t1	C14B1.7a, C16C10.3, R04A9.2, T22H9.3, Y49F6A.1	CBG01380, CBG21364
wago-11	OG0000406	Sp34_50015000.t1	C14B1.7a, C16C10.3, R04A9.2, T22H9.3, Y49F6A.1	CBG01380, CBG21364
nrde-3/wago-12	OG0000406	Sp34_50015000.t1	C14B1.7a, C16C10.3, R04A9.2, T22H9.3, Y49F6A.1	CBG01380, CBG21364
wago-1	OG0000120	Sp34_10010600.t1, Sp34_10087300.t1, Sp34_3002[illegible]	F11C3.3, F45G2.2a, F58G4.1, K12F2.1, R06C7.10, T18D3.4	CBG00120, CBG11357, CBG19730, CBG21212, CBG21911, CBG23416
wago-2	OG0000304	Sp34_10244410.t1, Sp34_10259300.t1	F55A12.1, F58G1.1, R06C7.1, Y110A7A.18, ZK1248.7	CBG09416, CBG12321, CBG23746
ppw-2/wago-3	OG0000304	Sp34_10244410.t1, Sp34_10259300.t1	F55A12.1, F58G1.1, R06C7.1, Y110A7A.18, ZK1248.7	CBG09416, CBG12321, CBG23746
wago-4	OG0000304	Sp34_10244410.t1, Sp34_10259300.t1	F55A12.1, F58G1.1, R06C7.1, Y110A7A.18, ZK1248.7	CBG09416, CBG12321, CBG23746
wago-5	OG0000304	Sp34_10244410.t1, Sp34_10259300.t1	F55A12.1, F58G1.1, R06C7.1, Y110A7A.18, ZK1248.7	CBG09416, CBG12321, CBG23746
sago-2/wago-6	OG0000768	Sp34_10261700.t1, Sp34_10263500.t1	C18E3.7a, F56A6.1a, K12B6.1	CBG12094
ppw-1/wago-7	OG0000768	Sp34_10261700.t1, Sp34_10263500.t1	C18E3.7a, F56A6.1a, K12B6.1	CBG12094
sago-1/wago-8	OG0000768	Sp34_10261700.t1, Sp34_10263500.t1	C18E3.7a, F56A6.1a, K12B6.1	CBG12094
hrde-1/wago-9	OG0000406	Sp34_50015000.t1	C14B1.7a, C16C10.3, R04A9.2, T22H9.3, Y49F6A.1	CBG01380, CBG21364

##21U RNA pathway factors

Gene	Orthogroup	*C. inopinata*	*C. elegans*	*C. briggsae*
fkh-3/4/5	OG0001887		C29F7.4a, C29F7.5, F26A1.2	CBG19636
unc-130	OG0009107	Sp34_30231500.t1	C02F5.6b	CBG16658
prg-1	OG0001646	Sp34_10098600.t1	C01G5.2a, D2030.6	CBG11957
henn-1	OG0003317	Sp34_20332900.t1	C47G2.2	CBG02936

Supplementary Table 20. Gene numbers of GPCR families in the three Caenorhabditis species

	*C. inopinata**	*C. elegans*	*C. briggsae*	Genes not found in *C. inopinata*
odr	8	7	7	
sra	24	35	19	*sra-17, sra-18, sra-20, sra-21, sra-22, sra-23, sra-24, sra-25, sra-26, sra-28, sra-29, sra-31, sra-33, sra-34, sra-36*
srab	10	23	12	*srab-25*
srb	5 (1)	16	10	*srb-17*
srbc	8	70	6	*srbc-29, srbc-30, srbc-31, srbc-32, srbc-33, srbc-34, srbc-36, srbc-37, srbc-38, srbc-39, srbc-43, srbc-44, srbc-45*
srd	35 (3)	67	35	*srd-1, srd-10, srd-15, srd-16, srd-2, srd-21, srd-23, srd-25, srd-26, srd-27, srd-28, srd-29, srd-3, srd-30, srd-31, srd-34, srd-35, srd-36, srd-38, srd-4, srd-40, srd-45, srd-46, srd-47, srd-48, srd-5, srd-50, srd-61, srd-63, srd-64, srd-65, srd-66, srd-67, srd-68, srd-69, srd-7, srd-70, srd-71, srd-72, srd-74, srd-75, srd-8*
sre	14	53	32	*sre-1, sre-14, sre-15, sre-16, sre-18, sre-19, sre-2, sre-20, sre-21, sre-24, sre-26, sre-29, sre-30, sre-31, sre-32, sre-33, sre-34, sre-35, sre-36, sre-38, sre-4, sre-43, sre-44, sre-45, sre-46, sre-47, sre-48, sre-49, sre-5, sre-50, sre-51, sre-52, sre-53, sre-54, sre-55, sre-56*
srg	30 (1)	61	25	*srg-15, srg-17, srg-26, srg-27, srg-28, srg-29, srg-30, srg-34, srg-36, srg-37, srg-38*
srh	33	224	42	*srh-1, srh-10, srh-100, srh-102, srh-104, srh-105, srh-109, srh-11, srh-111, srh-112, srh-113, srh-115, srh-116, srh-118, srh-119, srh-120, srh-122, srh-123, srh-128, srh-129, srh-130, srh-131, srh-132, srh-133, srh-134, srh-135, srh-146, srh-147, srh-148, srh-149, srh-15, srh-154, srh-159, srh-16, srh-165, srh-166, srh-167, srh-169, srh-17, srh-170, srh-171, srh-172, srh-173, srh-174, srh-177, srh-178, srh-179, srh-18, srh-180, srh-181, srh-182, srh-183, srh-184, srh-185, srh-19, srh-20, srh-21, srh-212, srh-213, srh-214, srh-215, srh-216, srh-217, srh-218, srh-219, srh-22, srh-220, srh-222, srh-223, srh-227, srh-228, srh-23, srh-231, srh-233, srh-234, srh-235, srh-236, srh-237, srh-255, srh-257, srh-258, srh-261, srh-264, srh-265, srh-266, srh-268, srh-276, srh-277, srh-278, srh-279, srh-281, srh-282, srh-283, srh-284, srh-286, srh-287, srh-288, srh-289, srh-290, srh-291, srh-292, srh-293, srh-295, srh-296, srh-297, srh-298, srh-299, srh-300, srh-301, srh-303, srh-304, srh-308, srh-40, srh-41, srh-42, srh-45, srh-48, srh-49, srh-50, srh-51, srh-76, srh-78, srh-79, srh-80, srh-82, srh-83, srh-85, srh-87, srh-88, srh-89, srh-92, srh-95, srh-97, srh-98, srh-99*
sri	7 (2)	61	14	*sri-10, sri-11, sri-12, sri-13, sri-14, sri-15, sri-16, sri-17, sri-18, sri-21, sri-24, sri-25, sri-26, sri-27, sri-28, sri-29, sri-30, sri-31, sri-4, sri-5, sri-6, sri-65, sri-66, sri-67, sri-69, sri-7, sri-71, sri-72, sri-73, sri-74, sri-77, sri-9, srj-10*
srj	5	40	11	*srj-11, srj-13, srj-14, srj-15, srj-16, srj-18, srj-19, srj-20, srj-21, srj-22, srj-23, srj-24, srj-25, srj-26, srj-27, srj-29, srj-32, srj-33, srj-37, srj-38, srj-39, srj-40, srj-42, srj-44, srj-45, srj-49, srj-5, srj-50, srj-52, srj-53, srj-54, srj-55, srj-57, srj-6*
srsx	22 (1)	37	22	*srsx-1, srsx-10, srsx-11, srsx-12, srsx-13, srsx-2, srsx-29, srsx-3, srsx-31, srsx-6, srsx-7, srsx-9*
srt	18 (1)	65	27	*srt-1, srt-11, srt-15, srt-16, srt-17, srt-18, srt-19, srt-2, srt-20, srt-22, srt-25, srt-26, srt-27, srt-28, srt-29, srt-3, srt-30, srt-31, srt-32, srt-33, srt-34, srt-35, srt-36, srt-37, srt-38, srt-39, srt-4, srt-40, srt-43, srt-5, srt-52, srt-55, srt-56, srt-58, srt-6, srt-7, srt-74, srt-8, srt-9*
sru	16 (4)	40	16	*sru-21, sru-22, sru-23, sru-24, sru-25, sru-26, sru-27, sru-28, sru-3, sru-33, sru-35, sru-36, sru-4, sru-42, sru-6*
srv	27	30	19	*srv-12, srv-16, srv-3, srv-4*
srw	38 (9)	115	18	*srw-1, srw-111, srw-112, srw-113, srw-115, srw-117, srw-118, srw-119, srw-120, srw-121, srw-122, srw-123, srw-124, srw-127, srw-129, srw-130, srw-132, srw-133, srw-134, srw-135, srw-136, srw-137, srw-138, srw-139, srw-140, srw-141, srw-142, srw-143, srw-144, srw-60, srw-61, srw-62, srw-63*
srx	35 (17)	105	44	*srx-1, srx-10, srx-104, srx-105, srx-108, srx-110, srx-111, srx-112, srx-113, srx-114, srx-118, srx-119, srx-120, srx-121, srx-122, srx-14, srx-16, srx-17, srx-18, srx-21, srx-22, srx-23, srx-24, srx-26, srx-28, srx-29, srx-3, srx-31, srx-32, srx-4, srx-43, srx-44, srx-48, srx-5, srx-50, srx-51, srx-53, srx-54, srx-55, srx-56, srx-58, srx-6, srx-60, srx-63, srx-65, srx-66, srx-67, srx-68, srx-7, srx-73, srx-74, srx-8, srx-82, srx-86, srx-87, srx-88, srx-9, srx-90, srx-92, srx-93*
srxa	5	17	9	*srxa-14, srxa-15, srxa-16, srxa-18, srxa-19, srxa-6, srxa-7, srxa-8, srxa-9*
srz	0	67	7	*srz-1, srz-10, srz-100, srz-101, srz-102, srz-103, srz-104, srz-105, srz-11, srz-12, srz-13, srz-14, srz-15, srz-16, srz-18, srz-19, srz-20, srz-23, srz-24, srz-25, srz-28, srz-29, srz-3, srz-31, srz-32, srz-37, srz-38, srz-4, srz-42, srz-43, srz-44, srz-47, srz-48, srz-5, srz-53, srz-54, srz-56, srz-58, srz-59, srz-6, srz-60, srz-61, srz-62, srz-63, srz-64, srz-66, srz-67, srz-69, srz-70, srz-71, srz-72, srz-74, srz-75, srz-76, srz-78, srz-79, srz-8, srz-82, srz-83, srz-85, srz-9, srz-90, srz-91, srz-94, srz-95, srz-96, srz-97, srz-99,*
str	42 (9)	196	77	*str-1, str-114, str-162, str-177, str-187, str-188, str-190, str-217, str-231, str-232, str-233, str-27, str-612, str-613, str-77, str-78, str-81, str-82, str-83, str-88, str-92, str-93*
Total	382 (48)	1329	452	

*Numbers in parentheses represents numbers of psudogene or trancated genes

Supplementary Table 21. Top enriched GO terms in non-synteny regions between *C. inopinata* and *C. elegans*

in *C. elegans* against *C. inopinata*

GO.ID	Term	Annotated	Significant	Expected	*p*-values
GO:0050907	detection of chemical stimulus involved in sensory perception	580	417	116.41	< 1e-30
GO:0007186	G-protein coupled receptor signaling pathway	766	365	153.75	< 1e-30
GO:0050911	detection of chemical stimulus involved in sensory perception of smell	280	191	56.2	< 1e-30
GO:0042048	olfactory behavior	293	186	58.81	< 1e-30
GO:0043401	steroid hormone mediated signaling pathway	281	125	56.4	5.80E-21
GO:0045087	innate immune response	296	114	59.41	1.00E-13
GO:0005975	carbohydrate metabolic process	278	101	55.8	1.60E-13
GO:0033692	cellular polysaccharide biosynthetic process	55	33	11.04	9.50E-11
GO:0006032	chitin catabolic process	40	27	8.03	9.90E-11
GO:0006516	glycoprotein catabolic process	21	16	4.21	4.80E-08
GO:0006355	regulation of transcription, DNA-templated	929	197	186.46	7.80E-08
GO:0051260	protein homooligomerization	56	29	11.24	1.20E-07
GO:0007606	sensory perception of chemical stimulus	772	475	154.95	2.90E-06

in *C. inopinata* against *C. elegans*

GO.ID	Term	Annotated	Significant	Expected	*p*-values
GO:0015074	DNA integration	2984	1384	765.04	< 1e-30
GO:0006508	proteolysis	2229	780	571.47	< 1e-30
GO:0006313	transposition, DNA-mediated	1880	769	482	< 1e-30
GO:0090305	nucleic acid phosphodiester bond hydrolysis	1883	678	482.77	< 1e-30
GO:0006278	RNA-dependent DNA biosynthetic process	849	363	217.67	2.80E-30
GO:0036065	fucosylation	102	74	26.15	3.60E-23
GO:0040021	hermaphrodite germ-line sex determination	61	49	15.64	5.10E-19
GO:0007186	G-protein coupled receptor signaling pathway	1110	350	284.58	4.10E-15
GO:0097676	histone H3-K36 dimethylation	418	167	107.17	6.70E-11
GO:0000729	DNA double-strand break processing	429	170	109.99	9.70E-11
GO:0051568	histone H3-K4 methylation	469	180	120.24	1.70E-10
GO:0005975	carbohydrate metabolic process	607	210	155.62	2.00E-10
GO:0050911	detection of chemical stimulus involved in sensory perception	120	63	30.77	2.80E-10
GO:0035335	peptidyl-tyrosine dephosphorylation	246	107	63.07	7.30E-10
GO:0017148	negative regulation of translation	151	61	38.71	1.10E-09
GO:0006303	double-strand break repair via nonhomologous end joining	450	171	115.37	3.60E-09
GO:0006032	chitin catabolic process	47	31	12.05	7.10E-09
GO:0042048	olfactory behavior	144	63	36.92	1.30E-07
GO:0050907	detection of chemical stimulus involved in sensory perception	226	114	57.94	4.10E-07
GO:0006468	protein phosphorylation	1411	376	361.75	7.40E-07
GO:0070121	Kupffer's vesicle development	29	20	7.44	1.20E-06
GO:0032012	regulation of ARF protein signal transduction	27	19	6.92	1.40E-06
GO:0006352	DNA-templated transcription, initiation	268	69	68.71	3.40E-06
GO:0032502	developmental process	4454	1020	1141.92	8.50E-06
GO:0045087	innate immune response	383	127	98.19	8.50E-06
GO:0031123	RNA 3'-end processing	146	40	37.43	9.00E-06
