# Supplementary material for: Biology and genome of a newly discovered sibling species of Caenorhabditis elegans
Source: Nat Commun. 2018 Aug 10;9:3216. doi: 10.1038/s41467-018-05712-5 (PMC6086898; doi:10.1038/s41467-018-05712-5)
Supplement: Supplementary file 2 — Description of Additional Supplementary Files [file 41467_2018_5712_MOESM2_ESM.pdf]

Description of Additional Supplementary Files

File Name: Supplementary Data 1
Description: Set of reactions used in the metabolic reconstruction of C. inopinata
